# Correction: Evidence of Online Performance Deterioration in User Sessions on Reddit

**DOI:** 10.1371/journal.pone.0165852

**Published:** 2016-10-27

**Authors:** The PLOS ONE Staff

The following information is missing from the Funding section: This work was partly funded by the Army Research Office contract W911NF-15-1-0142. The publisher apologizes for the error.
