# Supplementary material for: Evidence of Online Performance Deterioration in User Sessions on Reddit
Source: PLoS One. 2016 Aug 25;11(8):e0161636. doi: 10.1371/journal.pone.0161636 (PMC4999233; doi:10.1371/journal.pone.0161636)
Supplement: S1 Notebook — (HTML) [file pone.0161636.s001.html]

S1\_Notebook


In [ ]:

```
library(lme4)
options(rgl.useNULL=TRUE)
library(LMERConvenienceFunctions)

library(ggplot2)
options(jupyter.plot_mimetypes = 'image/png')
library(repr)
options(repr.plot.width=6, repr.plot.height=6)

library(car)
library(MASS)

library(vcd)
```

# Body length¶

In this notebook, we document our various steps taken to study the effect of the overall length of a session on the body length (number of characters) of the very first comment. Note that we only do this on a sample of 1 mio. data points here.

In [51]:

```
#no zeros in the outcome variable
data = read.csv("/home/psinger/Reddit-depletion/data/sample.csv", header=TRUE)
```

In [52]:

```
nrow(data)
```

Out[52]:

1000000

## Data inspection¶

We start by getting a better feeling of our data. Note that body\_length represents count data.

We limit the data to only consider the first comment of each session and limit for session lengths as stated in the paper.

In [53]:

```
idx = data$session_index==1 & data$session_comments<11
```

In [54]:

```
data_first = data[idx,]
```

In [55]:

```
nrow(data_first)
```

Out[55]:

494483

In [56]:

```
hist(data_first$body_length)
```

In [57]:

```
hist(data_first$body_length[data_first$body_length<1000])
```

## Regression model¶

We use mixed-effects models where in the most basic form (that we analyze in this notebook), we are interested in studying body\_length ~ 1 + session\_comments + (1|author) where body\_length is the text length of the first comment, session\_comments refers to the session length and author represents the redditor username.

### Linear mixed-effects regression¶

Even though we cannot expect a simple linear mixed-effects regression to be an appropriate model for our count data, we start with it.

In [58]:

```
m_lmer = lmer(body_length~1+session_comments+(1|author), data=data_first, REML=FALSE)
```

In [59]:

```
summary(m_lmer)
```

Out[59]:

```
Linear mixed model fit by maximum likelihood  ['lmerMod']
Formula: body_length ~ 1 + session_comments + (1 | author)
   Data: data_first

     AIC      BIC   logLik deviance df.resid 
 7034924  7034969 -3517458  7034916   494479 

Scaled residuals: 
   Min     1Q Median     3Q    Max 
-5.398 -0.390 -0.250  0.051 31.974 

Random effects:
 Groups   Name        Variance Std.Dev.
 author   (Intercept) 14825    121.8   
 Residual             74335    272.6   
Number of obs: 494483, groups:  author, 368403

Fixed effects:
                 Estimate Std. Error t value
(Intercept)      157.7048     0.6702  235.31
session_comments   6.6851     0.2974   22.48

Correlation of Fixed Effects:
            (Intr)
sssn_cmmnts -0.747
```

In [60]:

```
mcp.fnc(m_lmer)
```

What we can see in above plot is, that the residuals do not appear to be normally distributed and we can see clear heteroskedasticity. Thus, as expected, a linear model does not provide a good fit here.

### Linear mixed-effects regression (log-transform)¶

Next, let us take the log of the response variable and repeat a linear regression.

In [61]:

```
m_lmer_log = lmer(log(body_length)~1+session_comments+(1|author), data=data_first, REML=FALSE)
```

In [62]:

```
summary(m_lmer_log)
```

Out[62]:

```
Linear mixed model fit by maximum likelihood  ['lmerMod']
Formula: log(body_length) ~ 1 + session_comments + (1 | author)
   Data: data_first

      AIC       BIC    logLik  deviance  df.resid 
1614797.1 1614841.6 -807394.6 1614789.1    494479 

Scaled residuals: 
    Min      1Q  Median      3Q     Max 
-4.3482 -0.5529  0.0237  0.5891  3.7208 

Random effects:
 Groups   Name        Variance Std.Dev.
 author   (Intercept) 0.3446   0.587   
 Residual             1.2141   1.102   
Number of obs: 494483, groups:  author, 368403

Fixed effects:
                 Estimate Std. Error t value
(Intercept)      4.326976   0.002800  1545.1
session_comments 0.038680   0.001236    31.3

Correlation of Fixed Effects:
            (Intr)
sssn_cmmnts -0.740
```

In [63]:

```
mcp.fnc(m_lmer_log)
```

That looks pretty good already. For completeness, we proceed by looking at generalized linear mixed-effects models though as they usually capture count data quite well.

### Poisson GLMER¶

Let us start with a Poisson GLMER, which is a main choice when working with count data.

In [64]:

```
m_poisson = glmer(body_length~1+session_comments+(1|author),data=data_first,family=poisson())
```

```
Warning message:
In checkConv(attr(opt, "derivs"), opt$par, ctrl = control$checkConv, : Model is nearly unidentifiable: very large eigenvalue
 - Rescale variables?
```

In [65]:

```
summary(m_poisson)
```

Out[65]:

```
Generalized linear mixed model fit by maximum likelihood (Laplace
  Approximation) [glmerMod]
 Family: poisson  ( log )
Formula: body_length ~ 1 + session_comments + (1 | author)
   Data: data_first

      AIC       BIC    logLik  deviance  df.resid 
 24056344  24056378 -12028169  24056338    494480 

Scaled residuals: 
    Min      1Q  Median      3Q     Max 
-70.376  -0.441  -0.012   0.056 182.644 

Random effects:
 Groups Name        Variance Std.Dev.
 author (Intercept) 1.344    1.159   
Number of obs: 494483, groups:  author, 368403

Fixed effects:
                 Estimate Std. Error z value Pr(>|z|)    
(Intercept)      4.430014   0.001936  2287.9   <2e-16 ***
session_comments 0.021631   0.000131   165.1   <2e-16 ***
---
Signif. codes:  0 ‘***’ 0.001 ‘**’ 0.01 ‘*’ 0.05 ‘.’ 0.1 ‘ ’ 1

Correlation of Fixed Effects:
            (Intr)
sssn_cmmnts -0.110
convergence code: 0
Model is nearly unidentifiable: very large eigenvalue
 - Rescale variables?
```

In [66]:

```
plot(fitted(m_poisson),resid(m_poisson))
```

The convergence message shows low magnitude and for such large data this might be a false positive as emphasized in the lme4 docu.

The residual plots for generalized linear models are generally quite difficult to interpret as the residuals do not necessarily need to follow a normal distribution any longer. However, the fitted vs. residual plots shows a typical pattern for Poisson regression with curvilinear traces. What we can do though as a first step here, is to look at overdispersion (see http://glmm.wikidot.com/faq).

In [67]:

```
overdisp_fun <- function(model) {
  ## number of variance parameters in 
  ##   an n-by-n variance-covariance matrix
  vpars <- function(m) {
    nrow(m)*(nrow(m)+1)/2
  }
  model.df <- sum(sapply(VarCorr(model),vpars))+length(fixef(model))
  rdf <- nrow(model.frame(model))-model.df
  rp <- residuals(model,type="pearson")
  Pearson.chisq <- sum(rp^2)
  prat <- Pearson.chisq/rdf
  pval <- pchisq(Pearson.chisq, df=rdf, lower.tail=FALSE)
  c(chisq=Pearson.chisq,ratio=prat,rdf=rdf,p=pval)
}
```

In [68]:

```
overdisp_fun(m_poisson)
```

Out[68]:

chisq
:   17812536.1110739

ratio
:   36.0227635315359

rdf
:   494480

p
:   0

As expected, we have to deal with overdispersion. There are generally some ways of doing so: (i) adding an individual-level random effect or (ii) using a negative binomial model or (iii) using quasi likelihood. Let us start with the first option.

In [69]:

```
data_first$obs<-factor(seq.int(nrow(data_first)))
m_poisson2 = glmer(body_length~1+session_comments+(1|author)+(1|obs),data=data_first,family=poisson())
```

```
Warning message:
In checkConv(attr(opt, "derivs"), opt$par, ctrl = control$checkConv, : Model failed to converge with max|grad| = 0.00135948 (tol = 0.001, component 1)Warning message:
In checkConv(attr(opt, "derivs"), opt$par, ctrl = control$checkConv, : Model is nearly unidentifiable: very large eigenvalue
 - Rescale variables?
```

In [70]:

```
summary(m_poisson2)
```

Out[70]:

```
Generalized linear mixed model fit by maximum likelihood (Laplace
  Approximation) [glmerMod]
 Family: poisson  ( log )
Formula: body_length ~ 1 + session_comments + (1 | author) + (1 | obs)
   Data: data_first

     AIC      BIC   logLik deviance df.resid 
 5957084  5957129 -2978538  5957076   494479 

Scaled residuals: 
     Min       1Q   Median       3Q      Max 
-1.56051 -0.08605  0.00096  0.04079  0.13937 

Random effects:
 Groups Name        Variance Std.Dev.
 obs    (Intercept) 1.1214   1.0589  
 author (Intercept) 0.3357   0.5794  
Number of obs: 494483, groups:  obs, 494483; author, 368403

Fixed effects:
                 Estimate Std. Error z value Pr(>|z|)    
(Intercept)      4.345711   0.002727  1593.8   <2e-16 ***
session_comments 0.037943   0.001202    31.6   <2e-16 ***
---
Signif. codes:  0 ‘***’ 0.001 ‘**’ 0.01 ‘*’ 0.05 ‘.’ 0.1 ‘ ’ 1

Correlation of Fixed Effects:
            (Intr)
sssn_cmmnts -0.739
convergence code: 0
Model failed to converge with max|grad| = 0.00135948 (tol = 0.001, component 1)
Model is nearly unidentifiable: very large eigenvalue
 - Rescale variables?
```

In [71]:

```
overdisp_fun(m_poisson2)
```

Out[71]:

chisq
:   18516.8131744273

ratio
:   0.0374471174193995

rdf
:   494479

p
:   1

Great, the overdispersion problem is gone. The results (coefficients) are very similar to the log-normal model.

For completeness, we also want to utilize a negative binomial family though by first using the nb functionality provided in lme4.

In [ ]:

```
m_nb = glmer.nb(body_length~1+session_comments+(1|author),data=data_first)
```

In [91]:

```
summary(m_nb)
```

Out[91]:

```
Generalized linear mixed model fit by maximum likelihood (Laplace
  Approximation) [glmerMod]
 Family: Negative Binomial(1.2852)  ( log )
Formula: body_length ~ 1 + session_comments + (1 | author)
   Data: data_first

     AIC      BIC   logLik deviance df.resid 
 5980245  5980289 -2990118  5980237   494479 

Scaled residuals: 
    Min      1Q  Median      3Q     Max 
-1.1321 -0.6247 -0.1996  0.4147  8.6345 

Random effects:
 Groups Name        Variance Std.Dev.
 author (Intercept) 0.484    0.6957  
Number of obs: 494483, groups:  author, 368403

Fixed effects:
                  Estimate Std. Error z value Pr(>|z|)    
(Intercept)      4.6365497  0.0002340   19815   <2e-16 ***
session_comments 0.0407576  0.0002263     180   <2e-16 ***
---
Signif. codes:  0 ‘***’ 0.001 ‘**’ 0.01 ‘*’ 0.05 ‘.’ 0.1 ‘ ’ 1

Correlation of Fixed Effects:
            (Intr)
sssn_cmmnts -0.026
convergence code: 0
Model failed to converge with max|grad| = 0.247221 (tol = 0.001, component 1)
Model is nearly unidentifiable: very large eigenvalue
 - Rescale variables?
```

We end up with multiple convergence warnings (not shown); the coefficients are quite similar to our previous observations though. Note that this model also takes a very long time to fit for this sample of 1 mio. data points; it does not really scale for larger data which in our case consists of more than 50 mio. data points.

As a consequence, let us next also fit a negative binomial regression, but this time we use quasi likelihood.

In [ ]:

```
#just for initialization
init = glm.nb(body_length~1+session_comments,data=data_first)
```

In [92]:

```
init$theta
```

Out[92]:

0.81254007468633

In [ ]:

```
m_pql_nb = glmmPQL(body_length~1+session_comments, random = list(~1 | author), data = data_first, family = negative.binomial(theta=init$theta))
```

In [93]:

```
summary(m_pql_nb)
```

Out[93]:

```
Linear mixed-effects model fit by maximum likelihood
 Data: data_first 
  AIC BIC logLik
   NA  NA     NA

Random effects:
 Formula: ~1 | author
        (Intercept) Residual
StdDev:   0.6060416  0.80645

Variance function:
 Structure: fixed weights
 Formula: ~invwt 
Fixed effects: body_length ~ 1 + session_comments 
                    Value   Std.Error     DF   t-value p-value
(Intercept)      4.657142 0.002418961 368402 1925.2654       0
session_comments 0.041190 0.001054120 126079   39.0749       0
 Correlation: 
                 (Intr)
session_comments -0.727

Standardized Within-Group Residuals:
       Min         Q1        Med         Q3        Max 
-2.6866571 -0.6405718 -0.2196655  0.4187363 14.8852558 

Number of Observations: 494483
Number of Groups: 368403
```

Again, very similar results.

## Final model choice¶

Given our extensive analysis regarding the appropriate model for our data, we conclude that regardless of our model choice, the coefficients and subsequent inference are very similar. Nonetheless, we will proceed with a linear model on the log-transformed body\_length due to the appropriate diagnostic plots, the simplicity of the model and the high mean og body\_length.

## Significance of effects¶

Now that we have decided on a model, we want to extend our inference. First, we want to study the significance of the fixed effects as well as potential additional random effects.

There are several options for determining the significance of a fixed effect on the model. For example, a common approach is to use the ratio of the slope to its standard error and then use a t-test for determining its significance. Alternatively, one can also use F-tests for contrasting simpler to more complex models. Both methods require a specification of the degrees of freedom though which is not trivial in mixed models; methods such as the Satterthwaite approximation or Kenward-Roger approximation can be used for that task.
As stated in Baayen 2008, we can also directly use the t-statistics given to derive statistical significance. Because we have a huge dataset, a t-statistic above 2 can be regarded as significant at the 5\% level in a two-tailed test. You can derive this directly from the results above.

However, here, we follow an approach used for comparing models: the Bayesian Information Criterion (alternatively we could also work with AIC or LRT). Basically, the idea is to specify different (in this case nested) models that successively incorporate additional fixed or random effects and then compare their BIC scores. The lower the BIC, the better a model is (above a small threshold).

The baseline model we start with is:

body\_length = 1 + (1|author)

The reason why we incorporate the random effect for author from the beginning is that this is necessary based on the design of the experiment. Also, as pointed out in http://glmm.wikidot.com/faq, you should not compare the fit of a (g)lmer with one from a (g)lm.

Okay, so let us get the BIC for the baseline model.

In [ ]:

```
m1 = lmer(log(body_length)~1+(1|author), data = data_first, REML=FALSE)
```

In [94]:

```
summary(m1)
```

Out[94]:

```
Linear mixed model fit by maximum likelihood  ['lmerMod']
Formula: log(body_length) ~ 1 + (1 | author)
   Data: data_first

      AIC       BIC    logLik  deviance  df.resid 
1615774.2 1615807.5 -807884.1 1615768.2    494480 

Scaled residuals: 
    Min      1Q  Median      3Q     Max 
-4.3869 -0.5512  0.0252  0.5886  3.7049 

Random effects:
 Groups   Name        Variance Std.Dev.
 author   (Intercept) 0.3464   0.5885  
 Residual             1.2156   1.1025  
Number of obs: 494483, groups:  author, 368403

Fixed effects:
            Estimate Std. Error t value
(Intercept) 4.391856   0.001885    2329
```

Next, let us incorporate the session length.

In [ ]:

```
m2 = lmer(log(body_length)~1+session_comments+(1|author), data = data_first, REML=FALSE)
```

In [95]:

```
summary(m2)
```

Out[95]:

```
Linear mixed model fit by maximum likelihood  ['lmerMod']
Formula: log(body_length) ~ 1 + session_comments + (1 | author)
   Data: data_first

      AIC       BIC    logLik  deviance  df.resid 
1614797.1 1614841.6 -807394.6 1614789.1    494479 

Scaled residuals: 
    Min      1Q  Median      3Q     Max 
-4.3482 -0.5529  0.0237  0.5891  3.7208 

Random effects:
 Groups   Name        Variance Std.Dev.
 author   (Intercept) 0.3446   0.587   
 Residual             1.2141   1.102   
Number of obs: 494483, groups:  author, 368403

Fixed effects:
                 Estimate Std. Error t value
(Intercept)      4.326976   0.002800  1545.1
session_comments 0.038680   0.001236    31.3

Correlation of Fixed Effects:
            (Intr)
sssn_cmmnts -0.740
```

Based on the lower BIC we can clearly see that the incorporation of the session length improves our model.

We stop at this point as our scope of interest is covered. However, one can arbitrarily extend the existing model.
For example, one might also argue that it is necessary to incorporate a random effect for the intercept based on different subreddits.

In [ ]:

```
m3 = lmer(log(body_length)~1+session_comments+(1|author)+(1|subreddit), data = data_first, REML=FALSE)
```

In [96]:

```
summary(m3)
```

Out[96]:

```
Linear mixed model fit by maximum likelihood  ['lmerMod']
Formula: log(body_length) ~ 1 + session_comments + (1 | author) + (1 |  
    subreddit)
   Data: data_first

      AIC       BIC    logLik  deviance  df.resid 
1568439.2 1568494.8 -784214.6 1568429.2    494478 

Scaled residuals: 
    Min      1Q  Median      3Q     Max 
-5.2756 -0.5499  0.0344  0.5987  4.3417 

Random effects:
 Groups    Name        Variance Std.Dev.
 author    (Intercept) 0.2218   0.4709  
 subreddit (Intercept) 0.2788   0.5280  
 Residual              1.1523   1.0734  
Number of obs: 494483, groups:  author, 368403; subreddit, 14040

Fixed effects:
                 Estimate Std. Error t value
(Intercept)      4.392131   0.006968   630.3
session_comments 0.036124   0.001181    30.6

Correlation of Fixed Effects:
            (Intr)
sssn_cmmnts -0.263
```

Again, the BIC shows an improvement in the model. Yet, the inference on the fixed effects does not change. For complexity reasons, we neglect the random effect for the subreddit for the complete data.

### Outliers¶

We also want to check for robustness when removing outliers.

In [ ]:

```
data_first_limit <- romr.fnc(m2, data_first, trim = 2.5)$data
```

In [ ]:

```
m2_limit = lmer(log(body_length)~1+session_comments+(1|author),data=data_first_limit, REML=FALSE)
```

In [97]:

```
summary(m2_limit)
```

Out[97]:

```
Linear mixed model fit by maximum likelihood  ['lmerMod']
Formula: log(body_length) ~ 1 + session_comments + (1 | author)
   Data: data_first_limit

      AIC       BIC    logLik  deviance  df.resid 
1497635.2 1497679.6 -748813.6 1497627.2    485063 

Scaled residuals: 
     Min       1Q   Median       3Q      Max 
-2.89682 -0.59508  0.00823  0.59979  2.83525 

Random effects:
 Groups   Name        Variance Std.Dev.
 author   (Intercept) 0.3442   0.5867  
 Residual             0.9680   0.9839  
Number of obs: 485067, groups:  author, 363093

Fixed effects:
                 Estimate Std. Error t value
(Intercept)      4.367310   0.002591  1685.5
session_comments 0.037290   0.001138    32.8

Correlation of Fixed Effects:
            (Intr)
sssn_cmmnts -0.735
```

Not much changed.

## REML¶

Until now, we have fitted the models using ML as this allowed us to compare models with each other. To be sure regarding the consistency of our coefficient estimations, we will now also quickly fit the model with REML.

In [ ]:

```
m2_reml = lmer(log(body_length)~1+session_comments+(1|author), data = data_first, REML=TRUE)
```

In [98]:

```
summary(m2_reml)
```

Out[98]:

```
Linear mixed model fit by REML ['lmerMod']
Formula: log(body_length) ~ 1 + session_comments + (1 | author)
   Data: data_first

REML criterion at convergence: 1614811

Scaled residuals: 
    Min      1Q  Median      3Q     Max 
-4.3482 -0.5529  0.0237  0.5891  3.7208 

Random effects:
 Groups   Name        Variance Std.Dev.
 author   (Intercept) 0.3446   0.587   
 Residual             1.2141   1.102   
Number of obs: 494483, groups:  author, 368403

Fixed effects:
                 Estimate Std. Error t value
(Intercept)      4.326976   0.002800  1545.1
session_comments 0.038680   0.001236    31.3

Correlation of Fixed Effects:
            (Intr)
sssn_cmmnts -0.740
```

The results are exactly the same.

## Convergence problems¶

Convergence warnings (which we partly also see for experiments on the complete data) are most likely false positives and do not effect the results. The gold standard approach to investigate this is to utilize different optimizers and compare the coefficients. If they are similar, our inference is stable which is the case here. The code is based on Ben Bolkers code presented online: http://stackoverflow.com/questions/21344555/convergence-error-for-development-version-of-lme4

In [102]:

```
g0.bobyqa <- lmer(log(body_length)~1+session_comments+(1|author),
      data = data_first,
                   control=lmerControl(optimizer="bobyqa"))
g0.NM <- update(g0.bobyqa,control=lmerControl(optimizer="Nelder_Mead"))
library(optimx)
g0.nlminb <- update(g0.bobyqa,control=lmerControl(optimizer="optimx",
                              optCtrl=list(method="nlminb")))
g0.LBFGSB <- update(g0.bobyqa,control=lmerControl(optimizer="optimx",
                              optCtrl=list(method="L-BFGS-B")))
```

In [106]:

```
getpar <- function(x) c(getME(x,c("theta")),fixef(x))
modList <- list(bobyqa=g0.bobyqa,NM=g0.NM,nlminb=g0.nlminb,
                LBFGSB=g0.LBFGSB)
ctab <- sapply(modList,getpar)
library(reshape2)
mtab <- melt(ctab)
library(ggplot2)
theme_set(theme_bw())
ggplot(mtab,aes(x=Var2,y=value,colour=Var2))+
    geom_point()+facet_wrap(~Var1,scale="free")
```

In [ ]:

```

```
