# Supplementary material for: Evidence of Online Performance Deterioration in User Sessions on Reddit
Source: PLoS One. 2016 Aug 25;11(8):e0161636. doi: 10.1371/journal.pone.0161636 (PMC4999233; doi:10.1371/journal.pone.0161636)
Supplement: S2 Notebook — (HTML) [file pone.0161636.s002.html]

start\_num\_responses


In [42]:

```
library(lme4)
options(rgl.useNULL=TRUE)
library(LMERConvenienceFunctions)

library(ggplot2)
options(jupyter.plot_mimetypes = 'image/png')
library(repr)
options(repr.plot.width=6, repr.plot.height=6)

library(car)
library(MASS)

library(vcd)
```

# Num answers¶

In this notebook, we document our various steps taken to study the effect of the overall length of a session on the number of answers to the very first comment. Note that we only do this on a sample of 1 mio. data points here.

In [43]:

```
data = read.csv("/home/psinger/Reddit-depletion/data/sample.csv", header=TRUE)
```

In [44]:

```
nrow(data)
```

Out[44]:

1000000

## Data inspection¶

We start by getting a better feeling of our data. Note that num\_answers represents count data.

We limit the data to only consider the first comment of each session and limit for session lengths as stated in the paper.

In [45]:

```
idx = data$session_index==1 & data$session_comments<11
```

In [46]:

```
data_first = data[idx,]
```

In [47]:

```
nrow(data_first)
```

Out[47]:

494483

In [48]:

```
hist(data_first$num_answers)
```

In [49]:

```
hist(data_first$num_answers[data_first$num_answers<10])
```

## Regression model¶

We use mixed-effects models where in the most basic form (that we analyze in this notebook), we are interested in studying num\_answers ~ 1 + session\_comments + (1|author) where num\_answers is the number of answers the first comment receives, session\_comments refers to the session length and author represents the redditor username.

### Linear mixed-effects regression¶

Even though we cannot expect a simple linear mixed-effects regression to be an appropriate model for our count data, we start with it.

In [50]:

```
m_lmer = lmer(num_answers~1+session_comments+(1|author), data=data_first, REML=FALSE)
```

In [51]:

```
summary(m_lmer)
```

Out[51]:

```
Linear mixed model fit by maximum likelihood  ['lmerMod']
Formula: num_answers ~ 1 + session_comments + (1 | author)
   Data: data_first

      AIC       BIC    logLik  deviance  df.resid 
1789598.4 1789642.8 -894795.2 1789590.4    494479 

Scaled residuals: 
    Min      1Q  Median      3Q     Max 
 -3.889  -0.382  -0.379   0.285 271.813 

Random effects:
 Groups   Name        Variance Std.Dev.
 author   (Intercept) 0.0603   0.2456  
 Residual             2.1245   1.4576  
Number of obs: 494483, groups:  author, 368403

Fixed effects:
                 Estimate Std. Error t value
(Intercept)      0.525314   0.003298  159.28
session_comments 0.047161   0.001477   31.92

Correlation of Fixed Effects:
            (Intr)
sssn_cmmnts -0.765
```

In [52]:

```
mcp.fnc(m_lmer)
```

What we can see in above plot is, that the residuals do not appear to be normally distributed and we can see clear heteroskedasticity. Thus, as expected, a linear model does not provide a good fit here.

### Linear mixed-effects regression (log-transform)¶

Next, let us take the log of the response variable and repeat a linear regression.

In [53]:

```
m_lmer_log = lmer(log1p(num_answers)~1+session_comments+(1|author), data=data_first, REML=FALSE)
```

In [54]:

```
summary(m_lmer_log)
```

Out[54]:

```
Linear mixed model fit by maximum likelihood  ['lmerMod']
Formula: log1p(num_answers) ~ 1 + session_comments + (1 | author)
   Data: data_first

      AIC       BIC    logLik  deviance  df.resid 
 619376.4  619420.8 -309684.2  619368.4    494479 

Scaled residuals: 
    Min      1Q  Median      3Q     Max 
-1.6434 -0.7107 -0.7098  0.7904 12.3128 

Random effects:
 Groups   Name        Variance Std.Dev.
 author   (Intercept) 0.007913 0.08895 
 Residual             0.197079 0.44394 
Number of obs: 494483, groups:  author, 368403

Fixed effects:
                  Estimate Std. Error t value
(Intercept)      0.3049251  0.0010112  301.55
session_comments 0.0232390  0.0004527   51.33

Correlation of Fixed Effects:
            (Intr)
sssn_cmmnts -0.764
```

In [55]:

```
mcp.fnc(m_lmer_log)
```

That does not look satisfying at all. We should focus on generalized mixed-effects models to better model the count data.

### Poisson GLMER¶

Let us start with a Poisson GLMER, which is a main choice when working with count data.

In [56]:

```
m_poisson = glmer(num_answers~1+session_comments+(1|author),data=data_first,family=poisson())
```

```
Warning message:
In checkConv(attr(opt, "derivs"), opt$par, ctrl = control$checkConv, : Model failed to converge with max|grad| = 0.00156296 (tol = 0.001, component 1)Warning message:
In checkConv(attr(opt, "derivs"), opt$par, ctrl = control$checkConv, : Model is nearly unidentifiable: very large eigenvalue
 - Rescale variables?
```

In [57]:

```
summary(m_poisson)
```

Out[57]:

```
Generalized linear mixed model fit by maximum likelihood (Laplace
  Approximation) [glmerMod]
 Family: poisson  ( log )
Formula: num_answers ~ 1 + session_comments + (1 | author)
   Data: data_first

     AIC      BIC   logLik deviance df.resid 
 1037430  1037463  -518712  1037424   494480 

Scaled residuals: 
    Min      1Q  Median      3Q     Max 
-9.0286 -0.5835 -0.5563  0.6138 15.6626 

Random effects:
 Groups Name        Variance Std.Dev.
 author (Intercept) 0.5952   0.7715  
Number of obs: 494483, groups:  author, 368403

Fixed effects:
                  Estimate Std. Error z value Pr(>|z|)    
(Intercept)      -0.944834   0.003839 -246.11   <2e-16 ***
session_comments  0.070186   0.001378   50.95   <2e-16 ***
---
Signif. codes:  0 ‘***’ 0.001 ‘**’ 0.01 ‘*’ 0.05 ‘.’ 0.1 ‘ ’ 1

Correlation of Fixed Effects:
            (Intr)
sssn_cmmnts -0.647
convergence code: 0
Model failed to converge with max|grad| = 0.00156296 (tol = 0.001, component 1)
Model is nearly unidentifiable: very large eigenvalue
 - Rescale variables?
```

In [58]:

```
plot(fitted(m_poisson),resid(m_poisson))
```

The convergence message shows low magnitude and for such large data this might be a false positive as emphasized in the lme4 docu.

The residual plots for generalized linear models are generally quite difficult to interpret as the residuals do not necessarily need to follow a normal distribution any longer. However, the fitted vs. residual plots shows a typical pattern for Poisson regression with curvilinear traces. What we can do though as a first step here, is to look at overdispersion (see http://glmm.wikidot.com/faq).

In [59]:

```
overdisp_fun <- function(model) {
  ## number of variance parameters in 
  ##   an n-by-n variance-covariance matrix
  vpars <- function(m) {
    nrow(m)*(nrow(m)+1)/2
  }
  model.df <- sum(sapply(VarCorr(model),vpars))+length(fixef(model))
  rdf <- nrow(model.frame(model))-model.df
  rp <- residuals(model,type="pearson")
  Pearson.chisq <- sum(rp^2)
  prat <- Pearson.chisq/rdf
  pval <- pchisq(Pearson.chisq, df=rdf, lower.tail=FALSE)
  c(chisq=Pearson.chisq,ratio=prat,rdf=rdf,p=pval)
}
```

In [60]:

```
overdisp_fun(m_poisson)
```

Out[60]:

chisq
:   309799.206213001

ratio
:   0.626515139566821

rdf
:   494480

p
:   1

It does not seem that we have any problem with overdispersion here.

Let us be sure and check it with the dispersion\_glmer function from the blmeco packages.

In [61]:

```
library("blmeco") 
dispersion_glmer(m_poisson)
```

Out[61]:

0.971810619282066

The value is between 0.75 and 1.4 which gives an indicator that there is no overdispersion problem.

What might be an issue for our model though, is the vast presence of zeros in our data. In order to tackle this issue, one can look at zero-inflated Poisson models. Unfortunately, these are only implemented in the MCMCglmm and glmmADMB packages which both utilize complex fitting methods that do not scale to large data and are thus, not useful for our large data case.

To that end, we use a generic "implementation of the EM algorithm that alternates between fitting a GLMM with data that are weighted according to their zero probability, and fitting a binary FLM for the probability that a data point is zero"---for detailed reference please refer to https://groups.nceas.ucsb.edu/non-linear-modeling/projects/owls/WRITEUP/owls.pdf.

In [62]:

```
source("../owls_R_funs.R")
```

In [63]:

```
m_poisson_zi = zipme(cformula=num_answers~1+session_comments+(1|author), zformula=z~1,data=data_first,cfamily=poisson())
```

```
itr: 1
```

```
Warning message:
In checkConv(attr(opt, "derivs"), opt$par, ctrl = control$checkConv, : Model is nearly unidentifiable: very large eigenvalue
 - Rescale variables?
```

```
itr: 2
```

```
Warning message:
In checkConv(attr(opt, "derivs"), opt$par, ctrl = control$checkConv, : Model failed to converge with max|grad| = 0.00135615 (tol = 0.001, component 1)Warning message:
In checkConv(attr(opt, "derivs"), opt$par, ctrl = control$checkConv, : Model is nearly unidentifiable: very large eigenvalue
 - Rescale variables?
```

```
itr: 3
```

```
Warning message:
In checkConv(attr(opt, "derivs"), opt$par, ctrl = control$checkConv, : Model is nearly unidentifiable: very large eigenvalue
 - Rescale variables?
```

```
itr: 4
```

```
Warning message:
In checkConv(attr(opt, "derivs"), opt$par, ctrl = control$checkConv, : Model failed to converge with max|grad| = 0.00137264 (tol = 0.001, component 1)Warning message:
In checkConv(attr(opt, "derivs"), opt$par, ctrl = control$checkConv, : Model is nearly unidentifiable: very large eigenvalue
 - Rescale variables?
```

```
itr: 5
```

```
Warning message:
In checkConv(attr(opt, "derivs"), opt$par, ctrl = control$checkConv, : Model is nearly unidentifiable: very large eigenvalue
 - Rescale variables?
```

```
itr: 6
```

```
Warning message:
In checkConv(attr(opt, "derivs"), opt$par, ctrl = control$checkConv, : Model failed to converge with max|grad| = 0.00135065 (tol = 0.001, component 1)Warning message:
In checkConv(attr(opt, "derivs"), opt$par, ctrl = control$checkConv, : Model is nearly unidentifiable: very large eigenvalue
 - Rescale variables?
```

```
itr: 7
```

```
Warning message:
In checkConv(attr(opt, "derivs"), opt$par, ctrl = control$checkConv, : Model is nearly unidentifiable: very large eigenvalue
 - Rescale variables?
```

```
itr: 8
```

```
Warning message:
In checkConv(attr(opt, "derivs"), opt$par, ctrl = control$checkConv, : Model failed to converge with max|grad| = 0.00117913 (tol = 0.001, component 1)Warning message:
In checkConv(attr(opt, "derivs"), opt$par, ctrl = control$checkConv, : Model is nearly unidentifiable: very large eigenvalue
 - Rescale variables?
```

```
itr: 9
```

```
Warning message:
In checkConv(attr(opt, "derivs"), opt$par, ctrl = control$checkConv, : Model is nearly unidentifiable: very large eigenvalue
 - Rescale variables?
```

```
itr: 10
```

```
Warning message:
In checkConv(attr(opt, "derivs"), opt$par, ctrl = control$checkConv, : Model is nearly unidentifiable: very large eigenvalue
 - Rescale variables?
```

```
itr: 11
```

```
Warning message:
In checkConv(attr(opt, "derivs"), opt$par, ctrl = control$checkConv, : Model is nearly unidentifiable: very large eigenvalue
 - Rescale variables?
```

```
itr: 12
```

```
Warning message:
In checkConv(attr(opt, "derivs"), opt$par, ctrl = control$checkConv, : Model failed to converge with max|grad| = 0.00144662 (tol = 0.001, component 1)Warning message:
In checkConv(attr(opt, "derivs"), opt$par, ctrl = control$checkConv, : Model is nearly unidentifiable: very large eigenvalue
 - Rescale variables?
```

```
itr: 13
```

```
Warning message:
In checkConv(attr(opt, "derivs"), opt$par, ctrl = control$checkConv, : Model failed to converge with max|grad| = 0.00244959 (tol = 0.001, component 1)Warning message:
In checkConv(attr(opt, "derivs"), opt$par, ctrl = control$checkConv, : Model is nearly unidentifiable: very large eigenvalue
 - Rescale variables?
```

```
itr: 14
```

```
Warning message:
In checkConv(attr(opt, "derivs"), opt$par, ctrl = control$checkConv, : Model is nearly unidentifiable: very large eigenvalue
 - Rescale variables?
```

```
itr: 15
```

```
Warning message:
In checkConv(attr(opt, "derivs"), opt$par, ctrl = control$checkConv, : Model failed to converge with max|grad| = 0.00102558 (tol = 0.001, component 1)Warning message:
In checkConv(attr(opt, "derivs"), opt$par, ctrl = control$checkConv, : Model is nearly unidentifiable: very large eigenvalue
 - Rescale variables?
```

```
itr: 16
```

```
Warning message:
In checkConv(attr(opt, "derivs"), opt$par, ctrl = control$checkConv, : Model failed to converge with max|grad| = 0.00174901 (tol = 0.001, component 1)Warning message:
In checkConv(attr(opt, "derivs"), opt$par, ctrl = control$checkConv, : Model is nearly unidentifiable: very large eigenvalue
 - Rescale variables?
```

```
itr: 17
```

```
Warning message:
In checkConv(attr(opt, "derivs"), opt$par, ctrl = control$checkConv, : Model is nearly unidentifiable: very large eigenvalue
 - Rescale variables?
```

```
itr: 18
```

```
Warning message:
In checkConv(attr(opt, "derivs"), opt$par, ctrl = control$checkConv, : Model is nearly unidentifiable: very large eigenvalue
 - Rescale variables?
```

```
itr: 19
```

```
Warning message:
In checkConv(attr(opt, "derivs"), opt$par, ctrl = control$checkConv, : Model failed to converge with max|grad| = 0.00192292 (tol = 0.001, component 1)Warning message:
In checkConv(attr(opt, "derivs"), opt$par, ctrl = control$checkConv, : Model is nearly unidentifiable: very large eigenvalue
 - Rescale variables?
```

```
itr: 20
```

```
Warning message:
In checkConv(attr(opt, "derivs"), opt$par, ctrl = control$checkConv, : Model failed to converge with max|grad| = 0.00153061 (tol = 0.001, component 1)Warning message:
In checkConv(attr(opt, "derivs"), opt$par, ctrl = control$checkConv, : Model is nearly unidentifiable: very large eigenvalue
 - Rescale variables?
```

In [64]:

```
summary(m_poisson_zi)
```

Out[64]:

```
          Length Class    Mode   
zfit          30 glm      list   
cfit           1 glmerMod S4     
itr            1 -none-   numeric
deltainfo     20 -none-   numeric
z         494483 -none-   numeric
```

In [65]:

```
summary(m_poisson_zi$cfit)
```

Out[65]:

```
Generalized linear mixed model fit by maximum likelihood (Laplace
  Approximation) [glmerMod]
 Family: poisson  ( log )
Formula: num_answers ~ 1 + session_comments + (1 | author)
   Data: bydataw
Weights: (1 - z)

      AIC       BIC    logLik  deviance  df.resid 
1015027.6 1015060.9 -507510.8 1015021.6    494480 

Scaled residuals: 
    Min      1Q  Median      3Q     Max 
-7.2612 -0.5806 -0.5553  0.5877 15.6814 

Random effects:
 Groups Name        Variance Std.Dev.
 author (Intercept) 0.5753   0.7585  
Number of obs: 494483, groups:  author, 368403

Fixed effects:
                  Estimate Std. Error z value Pr(>|z|)    
(Intercept)      -0.902693   0.003826 -235.93   <2e-16 ***
session_comments  0.070039   0.001380   50.75   <2e-16 ***
---
Signif. codes:  0 ‘***’ 0.001 ‘**’ 0.01 ‘*’ 0.05 ‘.’ 0.1 ‘ ’ 1

Correlation of Fixed Effects:
            (Intr)
sssn_cmmnts -0.652
convergence code: 0
Model failed to converge with max|grad| = 0.00153061 (tol = 0.001, component 1)
Model is nearly unidentifiable: very large eigenvalue
 - Rescale variables?
```

Even though we see some convergence warnings (low magnitude, for each iteration), we can see is that the coefficients are very similar to the model fitted without zero-inflation. Comparing the BIC thoughs reveals a better fit in general for the ZIP.

Again, for completeness, we fit a negative binomial regression with quasi likelihood.

In [66]:

```
#just for initialization
init = glm.nb(num_answers~1+session_comments,data=data_first)
```

In [67]:

```
init$theta
```

Out[67]:

1.22525038734232

In [68]:

```
m_pql_nb = glmmPQL(num_answers~1+session_comments, random = list(~1 | author), data = data_first, family = negative.binomial(theta=init$theta))
```

```
iteration 1
iteration 2
iteration 3
iteration 4
iteration 5
iteration 6
iteration 7
iteration 8
iteration 9
iteration 10
```

In [69]:

```
summary(m_pql_nb)
```

Out[69]:

```
Linear mixed-effects model fit by maximum likelihood
 Data: data_first 
  AIC BIC logLik
   NA  NA     NA

Random effects:
 Formula: ~1 | author
        (Intercept)  Residual
StdDev:   0.7943736 0.6988311

Variance function:
 Structure: fixed weights
 Formula: ~invwt 
Fixed effects: num_answers ~ 1 + session_comments 
                      Value   Std.Error     DF    t-value p-value
(Intercept)      -0.9019146 0.003185255 368402 -283.15304       0
session_comments  0.0702522 0.001310569 126079   53.60434       0
 Correlation: 
                 (Intr)
session_comments -0.715

Standardized Within-Group Residuals:
       Min         Q1        Med         Q3        Max 
-2.6602585 -0.7120744 -0.6625956  0.5889095  9.8148781 

Number of Observations: 494483
Number of Groups: 368403
```

Again, very similar results.

## Final model choice¶

Given our extensive analysis regarding the appropriate model for our data, we can conclude that all have very similar conclusions, namely that the session length has a positive effect on num\_answers of the first comment in the session. Probably, the most appropriate model would be the zero-inflated Poisson regression. However, as the simple Poisson regression scales much better for larger data, we will continue to use it from this point on. Also, we do not use quasi likelihood, as it is difficult to compare the significance of fixed and random effects as no likelihood can be given; yet, it is reassuring that the coefficients are very similar to the Poisson models.

## Significance of effects¶

Now that we have decided on a model, we want to extend our inference. First, we want to study the significance of the fixed effects as well as potential additional random effects.

There are several options for determining the significance of a fixed effect on the model. For example, a common approach is to use the ratio of the slope to its standard error and then use a t-test for determining its significance. Alternatively, one can also use F-tests for contrasting simpler to more complex models. Both methods require a specification of the degrees of freedom though which is not trivial in mixed models; methods such as the Satterthwaite approximation or Kenward-Roger approximation can be used for that task.
As stated in Baayen 2008, we can also directly use the t-statistics given to derive statistical significance. Because we have a huge dataset, a t-statistic above 2 can be regarded as significant at the 5\% level in a two-tailed test. You can derive this directly from the results above.

However, here, we follow an approach used for comparing models: the Bayesian Information Criterion (alternatively we could also work with AIC or LRT). Basically, the idea is to specify different (in this case nested) models that successively incorporate additional fixed or random effects and then compare their BIC scores. The lower the BIC, the better a model is (above a small threshold).

The baseline model we start with is:

num\_answers = 1 + (1|author)

The reason why we incorporate the random effect for author from the beginning is that this is necessary based on the design of the experiment. Also, as pointed out in http://glmm.wikidot.com/faq, you should not compare the fit of a (g)lmer with one from a (g)lm.

Okay, so let us get the BIC for the baseline model.

In [70]:

```
m1 = glmer(num_answers~1+(1|author), data = data_first, family=poisson())
```

In [71]:

```
summary(m1)
```

Out[71]:

```
Generalized linear mixed model fit by maximum likelihood (Laplace
  Approximation) [glmerMod]
 Family: poisson  ( log )
Formula: num_answers ~ 1 + (1 | author)
   Data: data_first

     AIC      BIC   logLik deviance df.resid 
 1039904  1039926  -519950  1039900   494481 

Scaled residuals: 
    Min      1Q  Median      3Q     Max 
-9.5417 -0.5954 -0.5518  0.5735 15.2965 

Random effects:
 Groups Name        Variance Std.Dev.
 author (Intercept) 0.5988   0.7738  
Number of obs: 494483, groups:  author, 368403

Fixed effects:
             Estimate Std. Error z value Pr(>|z|)    
(Intercept) -0.824676   0.002934  -281.1   <2e-16 ***
---
Signif. codes:  0 ‘***’ 0.001 ‘**’ 0.01 ‘*’ 0.05 ‘.’ 0.1 ‘ ’ 1
```

Next, let us incorporate the session length.

In [72]:

```
m2 = glmer(num_answers~1+session_comments+(1|author), data = data_first, family=poisson())
```

```
Warning message:
In checkConv(attr(opt, "derivs"), opt$par, ctrl = control$checkConv, : Model failed to converge with max|grad| = 0.00156296 (tol = 0.001, component 1)Warning message:
In checkConv(attr(opt, "derivs"), opt$par, ctrl = control$checkConv, : Model is nearly unidentifiable: very large eigenvalue
 - Rescale variables?
```

In [73]:

```
summary(m2)
```

Out[73]:

```
Generalized linear mixed model fit by maximum likelihood (Laplace
  Approximation) [glmerMod]
 Family: poisson  ( log )
Formula: num_answers ~ 1 + session_comments + (1 | author)
   Data: data_first

     AIC      BIC   logLik deviance df.resid 
 1037430  1037463  -518712  1037424   494480 

Scaled residuals: 
    Min      1Q  Median      3Q     Max 
-9.0286 -0.5835 -0.5563  0.6138 15.6626 

Random effects:
 Groups Name        Variance Std.Dev.
 author (Intercept) 0.5952   0.7715  
Number of obs: 494483, groups:  author, 368403

Fixed effects:
                  Estimate Std. Error z value Pr(>|z|)    
(Intercept)      -0.944834   0.003839 -246.11   <2e-16 ***
session_comments  0.070186   0.001378   50.95   <2e-16 ***
---
Signif. codes:  0 ‘***’ 0.001 ‘**’ 0.01 ‘*’ 0.05 ‘.’ 0.1 ‘ ’ 1

Correlation of Fixed Effects:
            (Intr)
sssn_cmmnts -0.647
convergence code: 0
Model failed to converge with max|grad| = 0.00156296 (tol = 0.001, component 1)
Model is nearly unidentifiable: very large eigenvalue
 - Rescale variables?
```

Based on the lower BIC we can clearly see that the incorporation of the session\_comments improves our model, thus, we make inference on it (also the t-value indicates high significance).

We stop at this point as our scope of interest is covered. However, one can arbitrarily extend the existing model.
For example, one might also argue that it is necessary to incorporate a random effect for the intercept based on different subreddits.

In [ ]:

```
m3 = glmer(num_answers~1+session_comments+(1|author)+(1|subreddit), data = data_first, family=poisson())
```

In [78]:

```
summary(m3)
```

Out[78]:

```
Generalized linear mixed model fit by maximum likelihood (Laplace
  Approximation) [glmerMod]
 Family: poisson  ( log )
Formula: num_answers ~ 1 + session_comments + (1 | author) + (1 | subreddit)
   Data: data_first

     AIC      BIC   logLik deviance df.resid 
 1037216  1037260  -518604  1037208   494479 

Scaled residuals: 
    Min      1Q  Median      3Q     Max 
-7.6744 -0.6316 -0.5779  0.5274 14.5395 

Random effects:
 Groups    Name        Variance Std.Dev.
 author    (Intercept) 0.542151 0.73631 
 subreddit (Intercept) 0.008722 0.09339 
Number of obs: 494483, groups:  author, 368403; subreddit, 14040

Fixed effects:
                  Estimate Std. Error z value Pr(>|z|)    
(Intercept)      -0.934665   0.004608 -202.84   <2e-16 ***
session_comments  0.068017   0.001373   49.54   <2e-16 ***
---
Signif. codes:  0 ‘***’ 0.001 ‘**’ 0.01 ‘*’ 0.05 ‘.’ 0.1 ‘ ’ 1

Correlation of Fixed Effects:
            (Intr)
sssn_cmmnts -0.513
convergence code: 0
Model is nearly unidentifiable: very large eigenvalue
 - Rescale variables?
```

Again, the BIC shows an improvement in the model. Yet, the inference on the fixed effects does not change. For complexity reasons, we neglect the random effect for the subreddit for the complete data.

## Convergence problems¶

Convergence warnings (which we partly also see for experiments on the complete data) are most likely false positives and do not effect the results. The gold standard approach to investigate this is to utilize different optimizers and compare the coefficients. If they are similar, our inference is stable which is the case here. The code is based on Ben Bolkers code presented online: http://stackoverflow.com/questions/21344555/convergence-error-for-development-version-of-lme4

In [ ]:

```
g0.bobyqa <- glmer(num_answers~1+session_comments+(1|author),
      family = poisson, data = data_first,
                   control=glmerControl(optimizer="bobyqa"))
g0.NM <- update(g0.bobyqa,control=glmerControl(optimizer="Nelder_Mead"))
library(optimx)
g0.nlminb <- update(g0.bobyqa,control=glmerControl(optimizer="optimx",
                              optCtrl=list(method="nlminb")))
g0.LBFGSB <- update(g0.bobyqa,control=glmerControl(optimizer="optimx",
                              optCtrl=list(method="L-BFGS-B")))

library(nloptr)
## from https://github.com/lme4/lme4/issues/98:
defaultControl <- list(algorithm="NLOPT_LN_BOBYQA",xtol_rel=1e-6,maxeval=1e5)
nloptwrap2 <- function(fn,par,lower,upper,control=list(),...) {
    for (n in names(defaultControl)) 
      if (is.null(control[[n]])) control[[n]] <- defaultControl[[n]]
    res <- nloptr(x0=par,eval_f=fn,lb=lower,ub=upper,opts=control,...)
    with(res,list(par=solution,
                  fval=objective,
                  feval=iterations,
                  conv=if (status>0) 0 else status,
                  message=message))
}
g0.bobyqa2 <- update(g0.bobyqa,control=glmerControl(optimizer=nloptwrap2))
g0.NM2 <- update(g0.bobyqa,control=glmerControl(optimizer=nloptwrap2,
                           optCtrl=list(algorithm="NLOPT_LN_NELDERMEAD")))
```

In [79]:

```
getpar <- function(x) c(getME(x,c("theta")),fixef(x))
modList <- list(bobyqa=g0.bobyqa,NM=g0.NM,nlminb=g0.nlminb,
                bobyqa2=g0.bobyqa2,NM2=g0.NM2,LBFGSB=g0.LBFGSB)
ctab <- sapply(modList,getpar)
library(reshape2)
mtab <- melt(ctab)
library(ggplot2)
theme_set(theme_bw())
ggplot(mtab,aes(x=Var2,y=value,colour=Var2))+
    geom_point()+facet_wrap(~Var1,scale="free")
```

In [ ]:

```

```
