# Supplementary material for: Evidence of Online Performance Deterioration in User Sessions on Reddit
Source: PLoS One. 2016 Aug 25;11(8):e0161636. doi: 10.1371/journal.pone.0161636 (PMC4999233; doi:10.1371/journal.pone.0161636)
Supplement: S3 Notebook — (HTML) [file pone.0161636.s003.html]

start\_score


In [32]:

```
library(lme4)
options(rgl.useNULL=TRUE)
library(LMERConvenienceFunctions)

library(ggplot2)
options(jupyter.plot_mimetypes = 'image/png')
library(repr)
options(repr.plot.width=6, repr.plot.height=6)

library(car)
library(MASS)

library(vcd)
```

# Score¶

In this notebook, we document our various steps taken to study the effect of the overall length of a session on the score of the very first comment. Note that we only do this on a sample of 1 mio. data points here.

In [33]:

```
data = read.csv("/home/psinger/Reddit-depletion/data/sample.csv", header=TRUE)
```

In [34]:

```
nrow(data)
```

Out[34]:

1000000

## Data inspection¶

We start by getting a better feeling of our data.

We limit the data to only consider the first comment of each session and limit for session lengths as stated in the paper.

In [35]:

```
idx = data$session_index==1 & data$session_comments<11
```

In [36]:

```
data_first = data[idx,]
```

In [37]:

```
nrow(data_first)
```

Out[37]:

494483

In [38]:

```
hist(data_first$score)
```

In [39]:

```
hist(data_first$score[data_first$score<10&data_first$score>-10])
```

## Regression model¶

We use mixed-effects models where in the most basic form (that we analyze in this notebook), we are interested in studying score ~ 1 + session\_comments + (1|author) where score is the score the first comment received, session\_comments refers to the session length and author represents the redditor username.

### Linear mixed-effects regression¶

We start with a linear mixed-effects model.

In [40]:

```
m_lmer = lmer(score~1+session_comments+(1|author), data=data_first, REML=FALSE)
```

In [41]:

```
summary(m_lmer)
```

Out[41]:

```
Linear mixed model fit by maximum likelihood  ['lmerMod']
Formula: score ~ 1 + session_comments + (1 | author)
   Data: data_first

     AIC      BIC   logLik deviance df.resid 
 5349951  5349995 -2674971  5349943   494479 

Scaled residuals: 
    Min      1Q  Median      3Q     Max 
-23.396  -0.097  -0.094  -0.060  96.551 

Random effects:
 Groups   Name        Variance Std.Dev.
 author   (Intercept)   38.72   6.222  
 Residual             2887.46  53.735  
Number of obs: 494483, groups:  author, 368403

Fixed effects:
                 Estimate Std. Error t value
(Intercept)       6.06803    0.12054   50.34
session_comments  0.19623    0.05403    3.63

Correlation of Fixed Effects:
            (Intr)
sssn_cmmnts -0.767
```

In [42]:

```
mcp.fnc(m_lmer)
```

What we can see in above plot is, that the residuals do not appear to be normally distributed and we can see clear heteroskedasticity. Thus, a linear model does not provide a good fit here.

### Linear mixed-effects regression (log-transform)¶

Next, let us take the log of the response variable and repeat a linear regression. However, we first add a constant to the data in order to make all outcomes positive.

In [43]:

```
min(data_first$score)
data_first$score.t = data_first$score + abs(min(data_first$score))
```

Out[43]:

-1267

In [44]:

```
m_lmer_log = lmer(log1p(score.t)~1+session_comments+(1|author), data=data_first, REML=FALSE)
```

In [45]:

```
summary(m_lmer_log)
```

Out[45]:

```
Linear mixed model fit by maximum likelihood  ['lmerMod']
Formula: log1p(score.t) ~ 1 + session_comments + (1 | author)
   Data: data_first

     AIC      BIC   logLik deviance df.resid 
-2102270 -2102226  1051139 -2102278   494479 

Scaled residuals: 
     Min       1Q   Median       3Q      Max 
-245.357   -0.127   -0.121   -0.073   56.098 

Random effects:
 Groups   Name        Variance  Std.Dev.
 author   (Intercept) 1.541e-05 0.003925
 Residual             8.186e-04 0.028612
Number of obs: 494483, groups:  author, 368403

Fixed effects:
                  Estimate Std. Error t value
(Intercept)      7.150e+00  6.439e-05  111042
session_comments 8.995e-05  2.885e-05       3

Correlation of Fixed Effects:
            (Intr)
sssn_cmmnts -0.767
```

In [46]:

```
mcp.fnc(m_lmer_log)
```

Not much better. We should focus on generalized mixed-effects models to better model the count data.

### Poisson GLMER¶

Let us start with a Poisson GLMER, which is a main choice when working with count data.

In [47]:

```
m_poisson = glmer(score.t~1+session_comments+(1|author),data=data_first,family=poisson(link=log))
```

```
Warning message:
In checkConv(attr(opt, "derivs"), opt$par, ctrl = control$checkConv, : Model is nearly unidentifiable: very large eigenvalue
 - Rescale variables?
```

In [65]:

```
summary(m_poisson)
```

Out[65]:

```
Generalized linear mixed model fit by maximum likelihood (Laplace
  Approximation) [glmerMod]
 Family: poisson  ( log )
Formula: score.t ~ 1 + session_comments + (1 | author)
   Data: data_first

     AIC      BIC   logLik deviance df.resid 
 5117887  5117920 -2558940  5117881   494480 

Scaled residuals: 
    Min      1Q  Median      3Q     Max 
-31.519  -0.086  -0.068  -0.033  94.888 

Random effects:
 Groups Name        Variance  Std.Dev.
 author (Intercept) 0.0004712 0.02171 
Number of obs: 494483, groups:  author, 368403

Fixed effects:
                  Estimate Std. Error z value Pr(>|z|)    
(Intercept)      7.149e+00  7.873e-05   90806  < 2e-16 ***
session_comments 1.584e-04  3.395e-05       5  3.1e-06 ***
---
Signif. codes:  0 ‘***’ 0.001 ‘**’ 0.01 ‘*’ 0.05 ‘.’ 0.1 ‘ ’ 1

Correlation of Fixed Effects:
            (Intr)
sssn_cmmnts -0.717
convergence code: 0
Model is nearly unidentifiable: very large eigenvalue
 - Rescale variables?
```

In [49]:

```
plot(fitted(m_poisson), resid(m_poisson))
```

The convergence message shows low magnitude and for such large data this might be a false positive as emphasized in the lme4 docu.

The residual plots for generalized linear models are generally quite difficult to interpret as the residuals do not necessarily need to follow a normal distribution any longer. However, the fitted vs. residual plots shows a typical pattern for Poisson regression with curvilinear traces. What we can do though as a first step here, is to look at overdispersion (see http://glmm.wikidot.com/faq).

In [50]:

```
overdisp_fun <- function(model) {
  ## number of variance parameters in 
  ##   an n-by-n variance-covariance matrix
  vpars <- function(m) {
    nrow(m)*(nrow(m)+1)/2
  }
  model.df <- sum(sapply(VarCorr(model),vpars))+length(fixef(model))
  rdf <- nrow(model.frame(model))-model.df
  rp <- residuals(model,type="pearson")
  Pearson.chisq <- sum(rp^2)
  prat <- Pearson.chisq/rdf
  pval <- pchisq(Pearson.chisq, df=rdf, lower.tail=FALSE)
  c(chisq=Pearson.chisq,ratio=prat,rdf=rdf,p=pval)
}
```

In [51]:

```
overdisp_fun(m_poisson)
```

Out[51]:

chisq
:   351306.773465826

ratio
:   0.710456992124709

rdf
:   494480

p
:   1

It does not seem that we have any problem with overdispersion here.

Let us be sure and check it with the dispersion\_glmer function from the blmeco packages.

In [52]:

```
library("blmeco") 
dispersion_glmer(m_poisson)
```

Out[52]:

0.969081693732171

The value is between 0.75 and 1.4 which gives an indicator that there is no overdispersion problem.

For completeness, we fit a negative binomial regression with quasi likelihood.

In [53]:

```
#just for initialization
init = glm.nb(score.t~1+session_comments,data=data_first)
```

In [54]:

```
init$theta
```

Out[54]:

1976.59036542476

In [55]:

```
m_pql_nb = glmmPQL(score.t~1+session_comments, random = list(~1 | author), data = data_first, family = negative.binomial(theta=init$theta))
```

```
iteration 1
```

In [56]:

```
summary(m_pql_nb)
```

Out[56]:

```
Linear mixed-effects model fit by maximum likelihood
 Data: data_first 
  AIC BIC logLik
   NA  NA     NA

Random effects:
 Formula: ~1 | author
        (Intercept) Residual
StdDev: 0.004892255 1.174285

Variance function:
 Structure: fixed weights
 Formula: ~invwt 
Fixed effects: score.t ~ 1 + session_comments 
                    Value    Std.Error     DF  t-value p-value
(Intercept)      7.149185 9.465207e-05 368402 75531.21   0e+00
session_comments 0.000154 4.241721e-05 126079     3.63   3e-04
 Correlation: 
                 (Intr)
session_comments -0.767

Standardized Within-Group Residuals:
         Min           Q1          Med           Q3          Max 
-23.38786033  -0.09667899  -0.09392720  -0.05994872  96.55893067 

Number of Observations: 494483
Number of Groups: 368403
```

Again, very similar results.

## Final model choice¶

Given our extensive analysis regarding the appropriate model for our data, we can conclude that all have very similar conclusions, namely that the session length has a (slightly) positive effect on the score of the first comment in the session. For the rest of this section, let us focus on Poisson regression that seems to be appropriate for this count data at hand.

## Significance of effects¶

Now that we have decided on a model, we want to extend our inference. First, we want to study the significance of the fixed effects as well as potential additional random effects.

There are several options for determining the significance of a fixed effect on the model. For example, a common approach is to use the ratio of the slope to its standard error and then use a t-test for determining its significance. Alternatively, one can also use F-tests for contrasting simpler to more complex models. Both methods require a specification of the degrees of freedom though which is not trivial in mixed models; methods such as the Satterthwaite approximation or Kenward-Roger approximation can be used for that task.
As stated in Baayen 2008, we can also directly use the t-statistics given to derive statistical significance. Because we have a huge dataset, a t-statistic above 2 can be regarded as significant at the 5\% level in a two-tailed test. You can derive this directly from the results above.

However, here, we follow an approach used for comparing models: the Bayesian Information Criterion (alternatively we could also work with AIC or LRT). Basically, the idea is to specify different (in this case nested) models that successively incorporate additional fixed or random effects and then compare their BIC scores. The lower the BIC, the better a model is (above a small threshold).

The baseline model we start with is:

score = 1 + (1|author)

The reason why we incorporate the random effect for author from the beginning is that this is necessary based on the design of the experiment. Also, as pointed out in http://glmm.wikidot.com/faq, you should not compare the fit of a (g)lmer with one from a (g)lm.

Okay, so let us get the BIC for the baseline model.

In [57]:

```
m1 = glmer(score.t~1+(1|author), data = data_first, family=poisson())
```

```
Warning message:
In checkConv(attr(opt, "derivs"), opt$par, ctrl = control$checkConv, : Model is nearly unidentifiable: very large eigenvalue
 - Rescale variables?
```

In [58]:

```
summary(m1)
```

Out[58]:

```
Generalized linear mixed model fit by maximum likelihood (Laplace
  Approximation) [glmerMod]
 Family: poisson  ( log )
Formula: score.t ~ 1 + (1 | author)
   Data: data_first

     AIC      BIC   logLik deviance df.resid 
 5117907  5117929 -2558951  5117903   494481 

Scaled residuals: 
    Min      1Q  Median      3Q     Max 
-31.521  -0.088  -0.070  -0.035  94.883 

Random effects:
 Groups Name        Variance  Std.Dev.
 author (Intercept) 0.0004712 0.02171 
Number of obs: 494483, groups:  author, 368403

Fixed effects:
             Estimate Std. Error z value Pr(>|z|)    
(Intercept) 7.149e+00  5.491e-05  130193   <2e-16 ***
---
Signif. codes:  0 ‘***’ 0.001 ‘**’ 0.01 ‘*’ 0.05 ‘.’ 0.1 ‘ ’ 1
convergence code: 0
Model is nearly unidentifiable: very large eigenvalue
 - Rescale variables?
```

Next, let us incorporate the session length.

In [59]:

```
m2 = glmer(score.t~1+session_comments+(1|author), data = data_first, family=poisson())
```

```
Warning message:
In checkConv(attr(opt, "derivs"), opt$par, ctrl = control$checkConv, : Model is nearly unidentifiable: very large eigenvalue
 - Rescale variables?
```

In [60]:

```
summary(m2)
```

Out[60]:

```
Generalized linear mixed model fit by maximum likelihood (Laplace
  Approximation) [glmerMod]
 Family: poisson  ( log )
Formula: score.t ~ 1 + session_comments + (1 | author)
   Data: data_first

     AIC      BIC   logLik deviance df.resid 
 5117887  5117920 -2558940  5117881   494480 

Scaled residuals: 
    Min      1Q  Median      3Q     Max 
-31.519  -0.086  -0.068  -0.033  94.888 

Random effects:
 Groups Name        Variance  Std.Dev.
 author (Intercept) 0.0004712 0.02171 
Number of obs: 494483, groups:  author, 368403

Fixed effects:
                  Estimate Std. Error z value Pr(>|z|)    
(Intercept)      7.149e+00  7.873e-05   90806  < 2e-16 ***
session_comments 1.584e-04  3.395e-05       5  3.1e-06 ***
---
Signif. codes:  0 ‘***’ 0.001 ‘**’ 0.01 ‘*’ 0.05 ‘.’ 0.1 ‘ ’ 1

Correlation of Fixed Effects:
            (Intr)
sssn_cmmnts -0.717
convergence code: 0
Model is nearly unidentifiable: very large eigenvalue
 - Rescale variables?
```

Based on the lower BIC we can see that the incorporation of the session\_comments improves our model, thus, we make inference on it (also the t-value indicates significance).

We stop at this point as our scope of interest is covered. However, one can arbitrarily extend the existing model.
For example, one might also argue that it is necessary to incorporate a random effect for the intercept based on different subreddits.

In [61]:

```
m3 = glmer(score.t~1+session_comments+(1|author)+(1|subreddit), data = data_first, family=poisson())
```

```
Warning message:
In checkConv(attr(opt, "derivs"), opt$par, ctrl = control$checkConv, : Model failed to converge with max|grad| = 0.0383561 (tol = 0.001, component 1)Warning message:
In checkConv(attr(opt, "derivs"), opt$par, ctrl = control$checkConv, : Model is nearly unidentifiable: very large eigenvalue
 - Rescale variables?
```

In [62]:

```
summary(m3)
```

Out[62]:

```
Generalized linear mixed model fit by maximum likelihood (Laplace
  Approximation) [glmerMod]
 Family: poisson  ( log )
Formula: score.t ~ 1 + session_comments + (1 | author) + (1 | subreddit)
   Data: data_first

     AIC      BIC   logLik deviance df.resid 
 5115229  5115274 -2557611  5115221   494479 

Scaled residuals: 
    Min      1Q  Median      3Q     Max 
-31.466  -0.099  -0.045  -0.009  94.320 

Random effects:
 Groups    Name        Variance  Std.Dev.
 author    (Intercept) 4.645e-04 0.02155 
 subreddit (Intercept) 3.959e-06 0.00199 
Number of obs: 494483, groups:  author, 368403; subreddit, 14040

Fixed effects:
                  Estimate Std. Error z value Pr(>|z|)    
(Intercept)      7.147e+00  9.825e-05   72748  < 2e-16 ***
session_comments 1.444e-04  3.400e-05       4 2.16e-05 ***
---
Signif. codes:  0 ‘***’ 0.001 ‘**’ 0.01 ‘*’ 0.05 ‘.’ 0.1 ‘ ’ 1

Correlation of Fixed Effects:
            (Intr)
sssn_cmmnts -0.563
convergence code: 0
Model failed to converge with max|grad| = 0.0383561 (tol = 0.001, component 1)
Model is nearly unidentifiable: very large eigenvalue
 - Rescale variables?
```

Again, the BIC shows an improvement in the model. Yet, the inference on the fixed effects does not change.

## Convergence problems¶

Convergence warnings (which we partly also see for experiments on the complete data) are most likely false positives and do not effect the results. The gold standard approach to investigate this is to utilize different optimizers and compare the coefficients. If they are quite similar, our inference is stable which is the case here. The code is based on Ben Bolkers code presented online: http://stackoverflow.com/questions/21344555/convergence-error-for-development-version-of-lme4

In [63]:

```
g0.bobyqa <- glmer(score.t~1+session_comments+(1|author),
      family = poisson, data = data_first,
                   control=glmerControl(optimizer="bobyqa"))
g0.NM <- update(g0.bobyqa,control=glmerControl(optimizer="Nelder_Mead"))
library(optimx)
g0.nlminb <- update(g0.bobyqa,control=glmerControl(optimizer="optimx",
                              optCtrl=list(method="nlminb")))
g0.LBFGSB <- update(g0.bobyqa,control=glmerControl(optimizer="optimx",
                              optCtrl=list(method="L-BFGS-B")))

library(nloptr)
## from https://github.com/lme4/lme4/issues/98:
defaultControl <- list(algorithm="NLOPT_LN_BOBYQA",xtol_rel=1e-6,maxeval=1e5)
nloptwrap2 <- function(fn,par,lower,upper,control=list(),...) {
    for (n in names(defaultControl)) 
      if (is.null(control[[n]])) control[[n]] <- defaultControl[[n]]
    res <- nloptr(x0=par,eval_f=fn,lb=lower,ub=upper,opts=control,...)
    with(res,list(par=solution,
                  fval=objective,
                  feval=iterations,
                  conv=if (status>0) 0 else status,
                  message=message))
}
g0.bobyqa2 <- update(g0.bobyqa,control=glmerControl(optimizer=nloptwrap2))
g0.NM2 <- update(g0.bobyqa,control=glmerControl(optimizer=nloptwrap2,
                           optCtrl=list(algorithm="NLOPT_LN_NELDERMEAD")))
```

```
Warning message:
In checkConv(attr(opt, "derivs"), opt$par, ctrl = control$checkConv, : Model failed to converge with max|grad| = 0.00253142 (tol = 0.001, component 1)Warning message:
In checkConv(attr(opt, "derivs"), opt$par, ctrl = control$checkConv, : Model is nearly unidentifiable: very large eigenvalue
 - Rescale variables?Warning message:
In checkConv(attr(opt, "derivs"), opt$par, ctrl = control$checkConv, : Model failed to converge with max|grad| = 0.00549289 (tol = 0.001, component 1)Warning message:
In checkConv(attr(opt, "derivs"), opt$par, ctrl = control$checkConv, : Model is nearly unidentifiable: very large eigenvalue
 - Rescale variables?Warning message:
In optimx.check(par, optcfg$ufn, optcfg$ugr, optcfg$uhess, lower, : Parameters or bounds appear to have different scalings.
  This can cause poor performance in optimization. 
  It is important for derivative free methods like BOBYQA, UOBYQA, NEWUOA.Warning message:
In optwrap(optimizer, devfun, start, rho$lower, control = control, : convergence code 1 from optimxWarning message:
In checkConv(attr(opt, "derivs"), opt$par, ctrl = control$checkConv, : Model failed to converge with max|grad| = 1.90446 (tol = 0.001, component 1)Warning message:
In checkConv(attr(opt, "derivs"), opt$par, ctrl = control$checkConv, : Model is nearly unidentifiable: very large eigenvalue
 - Rescale variables?Warning message:
In optimx.check(par, optcfg$ufn, optcfg$ugr, optcfg$uhess, lower, : Parameters or bounds appear to have different scalings.
  This can cause poor performance in optimization. 
  It is important for derivative free methods like BOBYQA, UOBYQA, NEWUOA.Warning message:
In checkConv(attr(opt, "derivs"), opt$par, ctrl = control$checkConv, : Model failed to converge with max|grad| = 0.245149 (tol = 0.001, component 1)Warning message:
In checkConv(attr(opt, "derivs"), opt$par, ctrl = control$checkConv, : Model is nearly unidentifiable: very large eigenvalue
 - Rescale variables?Warning message:
In checkConv(attr(opt, "derivs"), opt$par, ctrl = control$checkConv, : Model failed to converge with max|grad| = 0.77017 (tol = 0.001, component 1)Warning message:
In checkConv(attr(opt, "derivs"), opt$par, ctrl = control$checkConv, : Model is nearly unidentifiable: very large eigenvalue
 - Rescale variables?Warning message:
In checkConv(attr(opt, "derivs"), opt$par, ctrl = control$checkConv, : Model failed to converge with max|grad| = 0.00217698 (tol = 0.001, component 1)Warning message:
In checkConv(attr(opt, "derivs"), opt$par, ctrl = control$checkConv, : Model is nearly unidentifiable: very large eigenvalue
 - Rescale variables?
```

In [64]:

```
getpar <- function(x) c(getME(x,c("theta")),fixef(x))
modList <- list(bobyqa=g0.bobyqa,NM=g0.NM,nlminb=g0.nlminb,
                bobyqa2=g0.bobyqa2,NM2=g0.NM2,LBFGSB=g0.LBFGSB)
ctab <- sapply(modList,getpar)
library(reshape2)
mtab <- melt(ctab)
library(ggplot2)
theme_set(theme_bw())
ggplot(mtab,aes(x=Var2,y=value,colour=Var2))+
    geom_point()+facet_wrap(~Var1,scale="free")
```
