# Supplementary material for: Evidence of Online Performance Deterioration in User Sessions on Reddit
Source: PLoS One. 2016 Aug 25;11(8):e0161636. doi: 10.1371/journal.pone.0161636 (PMC4999233; doi:10.1371/journal.pone.0161636)
Supplement: S4 Notebook — (HTML) [file pone.0161636.s004.html]

start\_readability


In [30]:

```
library(lme4)
options(rgl.useNULL=TRUE)
library(LMERConvenienceFunctions)

library(ggplot2)
options(jupyter.plot_mimetypes = 'image/png')
library(repr)
options(repr.plot.width=6, repr.plot.height=6)

library(car)
library(MASS)

library(vcd)
```

# Readability¶

In this notebook, we document our various steps taken to study the effect of the overall length of a session on the readability (flesch\_kincaid\_grade) of the very first comment. Note that we only do this on a sample of 1 mio. data points here.

In [31]:

```
data = read.csv("/home/psinger/Reddit-depletion/data/sample.csv", header=TRUE)
```

In [32]:

```
nrow(data)
```

Out[32]:

1000000

## Data inspection¶

We start by getting a better feeling of our data.

We limit the data to only consider the first comment of each session and limit for session lengths as stated in the paper.

In [33]:

```
idx = data$session_index==1 & data$session_comments<11
```

In [34]:

```
data_first = data[idx,]
```

In [35]:

```
nrow(data_first)
```

Out[35]:

494483

In [36]:

```
hist(data_first$flesch_kincaid_grade)
```

## Regression model¶

We use mixed-effects models where in the most basic form (that we analyze in this notebook), we are interested in studying flesch\_kincaid\_grade ~ 1 + session\_comments + (1|author) where flesch\_kincaid\_grade is the readability of the first comment, session\_comments refers to the session length and author represents the redditor username.

### Linear mixed-effects regression¶

We start with a linear mixed-effects model.

In [37]:

```
m_lmer = lmer(flesch_kincaid_grade~1+session_comments+(1|author), data=data_first, REML=FALSE)
```

In [38]:

```
summary(m_lmer)
```

Out[38]:

```
Linear mixed model fit by maximum likelihood  ['lmerMod']
Formula: flesch_kincaid_grade ~ 1 + session_comments + (1 | author)
   Data: data_first

     AIC      BIC   logLik deviance df.resid 
 2919248  2919292 -1459620  2919240   494479 

Scaled residuals: 
    Min      1Q  Median      3Q     Max 
-2.4220 -0.5677 -0.0436  0.5167  3.9946 

Random effects:
 Groups   Name        Variance Std.Dev.
 author   (Intercept)  2.356   1.535   
 Residual             19.182   4.380   
Number of obs: 494483, groups:  author, 368403

Fixed effects:
                 Estimate Std. Error t value
(Intercept)      4.996541   0.010406   480.1
session_comments 0.057542   0.004639    12.4

Correlation of Fixed Effects:
            (Intr)
sssn_cmmnts -0.755
```

In [39]:

```
mcp.fnc(m_lmer)
```

While not perfect, the residuals do not look to bad for large data here. The deviations from normality might be explained by the peaks in data at both ends of the distribution (large and low readability). Let us quickly try two transformations on the outcome (sqrt and log) for which we first set the outcome positive by adding a constant.

In [40]:

```
min(data_first$flesch_kincaid_grade)
data_first$flesch_kincaid_grade.t = data_first$flesch_kincaid_grade + 4.4
```

Out[40]:

-3.4

### Linear mixed-effects regression (transform)¶

In [41]:

```
m_lmer_log = lmer(log(flesch_kincaid_grade.t)~1+session_comments+(1|author), data=data_first, REML=FALSE)
```

In [42]:

```
summary(m_lmer_log)
```

Out[42]:

```
Linear mixed model fit by maximum likelihood  ['lmerMod']
Formula: log(flesch_kincaid_grade.t) ~ 1 + session_comments + (1 | author)
   Data: data_first

      AIC       BIC    logLik  deviance  df.resid 
 966827.2  966871.7 -483409.6  966819.2    494479 

Scaled residuals: 
    Min      1Q  Median      3Q     Max 
-3.5478 -0.2786  0.2008  0.5826  2.4038 

Random effects:
 Groups   Name        Variance Std.Dev.
 author   (Intercept) 0.04439  0.2107  
 Residual             0.37091  0.6090  
Number of obs: 494483, groups:  author, 368403

Fixed effects:
                  Estimate Std. Error t value
(Intercept)      2.0754187  0.0014449  1436.4
session_comments 0.0093903  0.0006442    14.6

Correlation of Fixed Effects:
            (Intr)
sssn_cmmnts -0.755
```

In [43]:

```
mcp.fnc(m_lmer_log)
```

Does not really look better.

In [44]:

```
m_lmer_sqrt = lmer(sqrt(flesch_kincaid_grade.t)~1+session_comments+(1|author), data=data_first, REML=FALSE)
```

In [45]:

```
summary(m_lmer_sqrt)
```

Out[45]:

```
Linear mixed model fit by maximum likelihood  ['lmerMod']
Formula: sqrt(flesch_kincaid_grade.t) ~ 1 + session_comments + (1 | author)
   Data: data_first

      AIC       BIC    logLik  deviance  df.resid 
1176302.6 1176347.1 -588147.3 1176294.6    494479 

Scaled residuals: 
    Min      1Q  Median      3Q     Max 
-2.9183 -0.4581  0.0823  0.5838  3.1829 

Random effects:
 Groups   Name        Variance Std.Dev.
 author   (Intercept) 0.0714   0.2672  
 Residual             0.5632   0.7505  
Number of obs: 494483, groups:  author, 368403

Fixed effects:
                  Estimate Std. Error t value
(Intercept)      2.9573469  0.0017865    1655
session_comments 0.0111383  0.0007962      14

Correlation of Fixed Effects:
            (Intr)
sssn_cmmnts -0.754
```

In [46]:

```
mcp.fnc(m_lmer_sqrt)
```

Looks quite similar to the non-transformed.

Let us focus on a potentially more appropriate Gamma generalized linear mixed-effects model next though.

### Gamma GLMER¶

Let us model the data with a Gamma GLMER using a log link.

In [47]:

```
m_gamma = glmer(flesch_kincaid_grade.t~1+session_comments+(1|author),data=data_first,family=Gamma(link="log"))
```

```
Warning message:
In checkConv(attr(opt, "derivs"), opt$par, ctrl = control$checkConv, : Model failed to converge with max|grad| = 0.00129599 (tol = 0.001, component 1)Warning message:
In checkConv(attr(opt, "derivs"), opt$par, ctrl = control$checkConv, : Model is nearly unidentifiable: very large eigenvalue
 - Rescale variables?
```

In [48]:

```
summary(m_gamma)
```

Out[48]:

```
Generalized linear mixed model fit by maximum likelihood (Laplace
  Approximation) [glmerMod]
 Family: Gamma  ( log )
Formula: flesch_kincaid_grade.t ~ 1 + session_comments + (1 | author)
   Data: data_first

     AIC      BIC   logLik deviance df.resid 
 2873226  2873270 -1436609  2873218   494479 

Scaled residuals: 
    Min      1Q  Median      3Q     Max 
-2.3029 -0.3326  0.1449  0.5257  7.1092 

Random effects:
 Groups   Name        Variance Std.Dev.
 author   (Intercept) 0.1972   0.4441  
 Residual             0.1638   0.4048  
Number of obs: 494483, groups:  author, 368403

Fixed effects:
                  Estimate Std. Error t value Pr(>|z|)    
(Intercept)      2.0674861  0.0015880  1301.9   <2e-16 ***
session_comments 0.0082267  0.0005727    14.4   <2e-16 ***
---
Signif. codes:  0 ‘***’ 0.001 ‘**’ 0.01 ‘*’ 0.05 ‘.’ 0.1 ‘ ’ 1

Correlation of Fixed Effects:
            (Intr)
sssn_cmmnts -0.581
convergence code: 0
Model failed to converge with max|grad| = 0.00129599 (tol = 0.001, component 1)
Model is nearly unidentifiable: very large eigenvalue
 - Rescale variables?
```

The convergence message shows low magnitude and for such large data this might be a false positive as emphasized in the lme4 docu.

The coefficients are quite similar to our previous analyses.

## Final model choice¶

Given our extensive analysis regarding the appropriate model for our data, we conclude that regardless of our model choice, the coefficients and subsequent inference are very similar. Nonetheless, we will proceed with a linear model on the flesch\_kincaid\_grade due to the reasonable diagnostic plots and the simplicity of the model. We thus accept slight deviations from normality of the residuals, the residuals do not indicate too much heteroskedasticity.

## Significance of effects¶

Now that we have decided on a model, we want to extend our inference. First, we want to study the significance of the fixed effects as well as potential additional random effects.

There are several options for determining the significance of a fixed effect on the model. For example, a common approach is to use the ratio of the slope to its standard error and then use a t-test for determining its significance. Alternatively, one can also use F-tests for contrasting simpler to more complex models. Both methods require a specification of the degrees of freedom though which is not trivial in mixed models; methods such as the Satterthwaite approximation or Kenward-Roger approximation can be used for that task.
As stated in Baayen 2008, we can also directly use the t-statistics given to derive statistical significance. Because we have a huge dataset, a t-statistic above 2 can be regarded as significant at the 5\% level in a two-tailed test. You can derive this directly from the results above.

However, here, we follow an approach used for comparing models: the Bayesian Information Criterion (alternatively we could also work with AIC or LRT). Basically, the idea is to specify different (in this case nested) models that successively incorporate additional fixed or random effects and then compare their BIC scores. The lower the BIC, the better a model is (above a small threshold).

The baseline model we start with is:

flesch\_kincaid\_grade = 1 + (1|author)

The reason why we incorporate the random effect for author from the beginning is that this is necessary based on the design of the experiment. Also, as pointed out in http://glmm.wikidot.com/faq, you should not compare the fit of a (g)lmer with one from a (g)lm.

Okay, so let us get the BIC for the baseline model.

In [49]:

```
m1 = lmer(flesch_kincaid_grade~1+(1|author), data = data_first, REML=FALSE)
```

In [63]:

```
summary(m1)
```

Out[63]:

```
Linear mixed model fit by maximum likelihood  ['lmerMod']
Formula: flesch_kincaid_grade ~ 1 + (1 | author)
   Data: data_first

     AIC      BIC   logLik deviance df.resid 
 2919400  2919433 -1459697  2919394   494480 

Scaled residuals: 
    Min      1Q  Median      3Q     Max 
-2.4227 -0.5717 -0.0429  0.5122  3.9879 

Random effects:
 Groups   Name        Variance Std.Dev.
 author   (Intercept)  2.364   1.538   
 Residual             19.181   4.380   
Number of obs: 494483, groups:  author, 368403

Fixed effects:
            Estimate Std. Error t value
(Intercept) 5.093963   0.006827   746.2
```

Next, let us incorporate the session length.

In [51]:

```
m2 = lmer(flesch_kincaid_grade~1+session_comments+(1|author), data = data_first, REML=FALSE)
```

In [64]:

```
summary(m2)
```

Out[64]:

```
Linear mixed model fit by maximum likelihood  ['lmerMod']
Formula: flesch_kincaid_grade ~ 1 + session_comments + (1 | author)
   Data: data_first

     AIC      BIC   logLik deviance df.resid 
 2919248  2919292 -1459620  2919240   494479 

Scaled residuals: 
    Min      1Q  Median      3Q     Max 
-2.4220 -0.5677 -0.0436  0.5167  3.9946 

Random effects:
 Groups   Name        Variance Std.Dev.
 author   (Intercept)  2.356   1.535   
 Residual             19.182   4.380   
Number of obs: 494483, groups:  author, 368403

Fixed effects:
                 Estimate Std. Error t value
(Intercept)      4.996541   0.010406   480.1
session_comments 0.057542   0.004639    12.4

Correlation of Fixed Effects:
            (Intr)
sssn_cmmnts -0.755
```

On this sample, we can see that the inclusion of the session lengths has a lower BIC.

We stop at this point as our scope of interest is covered. However, one can arbitrarily extend the existing model.
For example, one might also argue that it is necessary to incorporate a random effect for the intercept based on different subreddits.

In [54]:

```
m3 = lmer(flesch_kincaid_grade~1+session_comments+(1|author)+(1|subreddit), data = data_first, REML=FALSE)
```

In [55]:

```
summary(m3)
```

Out[55]:

```
Linear mixed model fit by maximum likelihood  ['lmerMod']
Formula: flesch_kincaid_grade ~ 1 + session_comments + (1 | author) +  
    (1 | subreddit)
   Data: data_first

     AIC      BIC   logLik deviance df.resid 
 2903195  2903251 -1451593  2903185   494478 

Scaled residuals: 
    Min      1Q  Median      3Q     Max 
-4.2622 -0.5886 -0.0485  0.5086  4.4493 

Random effects:
 Groups    Name        Variance Std.Dev.
 author    (Intercept)  1.68    1.296   
 subreddit (Intercept)  1.54    1.241   
 Residual              18.83    4.339   
Number of obs: 494483, groups:  author, 368403; subreddit, 14040

Fixed effects:
                 Estimate Std. Error t value
(Intercept)      4.974557   0.020758  239.64
session_comments 0.050942   0.004576   11.13

Correlation of Fixed Effects:
            (Intr)
sssn_cmmnts -0.349
```

Again, the BIC shows an improvement in the model. Yet, the inference on the fixed effects does not change much (it gets more pronounced though). For complexity reasons, we neglect the random effect for the subreddit for the complete data.

### Outliers¶

We also want to check for robustness when removing outliers.

In [56]:

```
data_first_limit <- romr.fnc(m2, data_first, trim = 2.5)$data
m2_limit = lmer(flesch_kincaid_grade~1+session_comments+(1|author),data=data_first_limit, REML=FALSE)
```

```
n.removed = 11450 
percent.removed = 2.31555
```

In [57]:

```
summary(m2_limit)
```

Out[57]:

```
Linear mixed model fit by maximum likelihood  ['lmerMod']
Formula: flesch_kincaid_grade ~ 1 + session_comments + (1 | author)
   Data: data_first_limit

     AIC      BIC   logLik deviance df.resid 
 2716596  2716640 -1358294  2716588   483029 

Scaled residuals: 
    Min      1Q  Median      3Q     Max 
-2.6904 -0.5761  0.0163  0.6162  3.2259 

Random effects:
 Groups   Name        Variance Std.Dev.
 author   (Intercept)  2.305   1.518   
 Residual             14.021   3.744   
Number of obs: 483033, groups:  author, 361641

Fixed effects:
                 Estimate Std. Error t value
(Intercept)      4.613093   0.009173   502.9
session_comments 0.068851   0.004077    16.9

Correlation of Fixed Effects:
            (Intr)
sssn_cmmnts -0.751
```

In [58]:

```
mcp.fnc(m2_limit)
```

What happens is that the residual diagnostics look better (expected). The effect of the session length is again positive (not changed) but a bit stronger.

## Convergence problems¶

Convergence warnings (which we partly also see for experiments on the complete data) are most likely false positives and do not effect the results. The gold standard approach to investigate this is to utilize different optimizers and compare the coefficients. If they are similar, our inference is stable which is the case here. The code is based on Ben Bolkers code presented online: http://stackoverflow.com/questions/21344555/convergence-error-for-development-version-of-lme4

In [62]:

```
g0.bobyqa <- lmer(flesch_kincaid_grade~1+session_comments+(1|author),
      data = data_first,
                   control=lmerControl(optimizer="bobyqa"))
g0.NM <- update(g0.bobyqa,control=lmerControl(optimizer="Nelder_Mead"))
library(optimx)
g0.nlminb <- update(g0.bobyqa,control=lmerControl(optimizer="optimx",
                              optCtrl=list(method="nlminb")))
g0.LBFGSB <- update(g0.bobyqa,control=lmerControl(optimizer="optimx",
                              optCtrl=list(method="L-BFGS-B")))
```

```
Warning message:
In checkConv(attr(opt, "derivs"), opt$par, ctrl = control$checkConv, : Model failed to converge with max|grad| = 0.0029294 (tol = 0.002, component 1)
```

```
Error in control$optimizer == "none": comparison (1) is possible only for atomic and list types
```

```
Error in control$optimizer == "none": comparison (1) is possible only for atomic and list types
```

In [65]:

```
getpar <- function(x) c(getME(x,c("theta")),fixef(x))
modList <- list(bobyqa=g0.bobyqa,NM=g0.NM,nlminb=g0.nlminb,
                LBFGSB=g0.LBFGSB)
ctab <- sapply(modList,getpar)
library(reshape2)
mtab <- melt(ctab)
library(ggplot2)
theme_set(theme_bw())
ggplot(mtab,aes(x=Var2,y=value,colour=Var2))+
    geom_point()+facet_wrap(~Var1,scale="free")
```

In [ ]:

```

```
