# Supplementary material for: Evidence of Online Performance Deterioration in User Sessions on Reddit
Source: PLoS One. 2016 Aug 25;11(8):e0161636. doi: 10.1371/journal.pone.0161636 (PMC4999233; doi:10.1371/journal.pone.0161636)
Supplement: S5 Notebook — (HTML) [file pone.0161636.s005.html]

dynamic\_text\_length


In [52]:

```
library(lme4)
options(rgl.useNULL=TRUE)
library(LMERConvenienceFunctions)

library(ggplot2)
options(jupyter.plot_mimetypes = 'image/png')
library(repr)
options(repr.plot.width=6, repr.plot.height=6)

library(car)
library(MASS)

library(vcd)
```

# Body length¶

In this notebook, we document our various steps taken to study the effect of the course of a session on the body length (number of characters). Note that we only do this on a sample of 1 mio. data points here.

In [53]:

```
#no zeros in the outcome variable
data = read.csv("/home/psinger/Reddit-depletion/data/sample.csv", header=TRUE)
```

In [54]:

```
nrow(data)
```

Out[54]:

1000000

We can limit our data to those sessions that have at least two and less than 11 comments.

In [55]:

```
idx = data$session_comments > 1 & data$session_comments < 11
data = data[idx,]
```

In [56]:

```
nrow(data)
```

Out[56]:

515196

## Data inspection¶

We start by getting a better feeling of our data. Note that body\_length represents count data.

In [57]:

```
hist(data$body_length)
```

In [58]:

```
hist(data$body_length[data$body_length<1000])
```

## Regression model¶

We use mixed-effects models where in the most basic form (that we analyze in this notebook), we are interested in studying body\_length ~ 1 + session\_index + (1|session\_comments) + (1|author).

### Linear mixed-effects regression¶

Even though we cannot expect a simple linear mixed-effects regression to be an appropriate model for our count data, we start with it.

In [59]:

```
m_lmer = lmer(body_length~1+session_index+session_comments+(1|author), data=data, REML=FALSE)
```

In [60]:

```
summary(m_lmer)
```

Out[60]:

```
Linear mixed model fit by maximum likelihood  ['lmerMod']
Formula: body_length ~ 1 + session_index + session_comments + (1 | author)
   Data: data

     AIC      BIC   logLik deviance df.resid 
 7246789  7246845 -3623390  7246779   515191 

Scaled residuals: 
   Min     1Q Median     3Q    Max 
-6.372 -0.412 -0.247  0.084 35.261 

Random effects:
 Groups   Name        Variance Std.Dev.
 author   (Intercept) 12897    113.6   
 Residual             63957    252.9   
Number of obs: 515196, groups:  author, 288629

Fixed effects:
                 Estimate Std. Error t value
(Intercept)      170.9110     0.8158  209.50
session_index     -5.2595     0.2833  -18.57
session_comments   3.6147     0.2222   16.27

Correlation of Fixed Effects:
            (Intr) sssn_n
session_ndx -0.169       
sssn_cmmnts -0.530 -0.642
```

In [61]:

```
mcp.fnc(m_lmer)
```

What we can see in above plot is, that the residuals do not appear to be normally distributed and we can see clear heteroskedasticity. Thus, as expected, a linear model does not provide a good fit here.

### Linear mixed-effects regression (log-transform)¶

Next, let us take the log of the response variable and repeat a linear regression.

In [62]:

```
m_lmer_log = lmer(log(body_length)~1+session_index+session_comments+(1|author), data=data, REML=FALSE)
```

In [63]:

```
summary(m_lmer_log)
```

Out[63]:

```
Linear mixed model fit by maximum likelihood  ['lmerMod']
Formula: log(body_length) ~ 1 + session_index + session_comments + (1 |  
    author)
   Data: data

      AIC       BIC    logLik  deviance  df.resid 
1649931.4 1649987.2 -824960.7 1649921.4    515191 

Scaled residuals: 
    Min      1Q  Median      3Q     Max 
-4.7413 -0.5556  0.0374  0.6037  4.0714 

Random effects:
 Groups   Name        Variance Std.Dev.
 author   (Intercept) 0.3303   0.5747  
 Residual             1.1621   1.0780  
Number of obs: 515196, groups:  author, 288629

Fixed effects:
                   Estimate Std. Error t value
(Intercept)       4.4384597  0.0035791  1240.1
session_index    -0.0209770  0.0012290   -17.1
session_comments  0.0181105  0.0009679    18.7

Correlation of Fixed Effects:
            (Intr) sssn_n
session_ndx -0.167       
sssn_cmmnts -0.526 -0.639
```

In [64]:

```
mcp.fnc(m_lmer_log)
```

That looks pretty good already. For completeness, we proceed by looking at generalized linear mixed-effects models though as they usually capture count data quite well.

### Poisson GLMER¶

Let us start with a Poisson GLMER, which is a main choice when working with count data.

In [65]:

```
m_poisson = glmer(body_length~1+session_index+session_comments+(1|author),data=data,family=poisson())
```

```
Warning message:
In checkConv(attr(opt, "derivs"), opt$par, ctrl = control$checkConv, : Model failed to converge with max|grad| = 0.00407952 (tol = 0.001, component 1)Warning message:
In checkConv(attr(opt, "derivs"), opt$par, ctrl = control$checkConv, : Model is nearly unidentifiable: very large eigenvalue
 - Rescale variables?
```

In [66]:

```
summary(m_poisson)
```

Out[66]:

```
Generalized linear mixed model fit by maximum likelihood (Laplace
  Approximation) [glmerMod]
 Family: poisson  ( log )
Formula: body_length ~ 1 + session_index + session_comments + (1 | author)
   Data: data

      AIC       BIC    logLik  deviance  df.resid 
 38014403  38014447 -19007197  38014395    515192 

Scaled residuals: 
    Min      1Q  Median      3Q     Max 
-69.779  -3.179  -0.045   0.908 196.933 

Random effects:
 Groups Name        Variance Std.Dev.
 author (Intercept) 1.195    1.093   
Number of obs: 515196, groups:  author, 288629

Fixed effects:
                   Estimate Std. Error z value Pr(>|z|)    
(Intercept)       4.568e+00  2.065e-03  2211.5   <2e-16 ***
session_index    -2.563e-02  1.084e-04  -236.4   <2e-16 ***
session_comments  1.853e-02  9.025e-05   205.3   <2e-16 ***
---
Signif. codes:  0 ‘***’ 0.001 ‘**’ 0.01 ‘*’ 0.05 ‘.’ 0.1 ‘ ’ 1

Correlation of Fixed Effects:
            (Intr) sssn_n
session_ndx -0.027       
sssn_cmmnts -0.091 -0.594
convergence code: 0
Model failed to converge with max|grad| = 0.00407952 (tol = 0.001, component 1)
Model is nearly unidentifiable: very large eigenvalue
 - Rescale variables?
```

In [67]:

```
plot(fitted(m_poisson),resid(m_poisson))
```

The convergence message shows low magnitude and for such large data this might be a false positive as emphasized in the lme4 docu.

The residual plots for generalized linear models are generally quite difficult to interpret as the residuals do not necessarily need to follow a normal distribution any longer. However, the fitted vs. residual plots shows a typical pattern for Poisson regression with curvilinear traces. What we can do though as a first step here, is to look at overdispersion (see http://glmm.wikidot.com/faq).

In [68]:

```
overdisp_fun <- function(model) {
  ## number of variance parameters in 
  ##   an n-by-n variance-covariance matrix
  vpars <- function(m) {
    nrow(m)*(nrow(m)+1)/2
  }
  model.df <- sum(sapply(VarCorr(model),vpars))+length(fixef(model))
  rdf <- nrow(model.frame(model))-model.df
  rp <- residuals(model,type="pearson")
  Pearson.chisq <- sum(rp^2)
  prat <- Pearson.chisq/rdf
  pval <- pchisq(Pearson.chisq, df=rdf, lower.tail=FALSE)
  c(chisq=Pearson.chisq,ratio=prat,rdf=rdf,p=pval)
}
```

In [69]:

```
overdisp_fun(m_poisson)
```

Out[69]:

chisq
:   34358623.4865417

ratio
:   66.6909103529202

rdf
:   515192

p
:   0

As expected, we have to deal with overdispersion. There are generally some ways of doing so: (i) adding an individual-level random effect or (ii) using a negative binomial model or (iii) using quasi likelihood. Let us start with the first option.

In [70]:

```
data$obs<-factor(seq.int(nrow(data)))
m_poisson2 = glmer(body_length~1+session_index+session_comments+(1|author)+(1|obs),data=data,family=poisson())
```

```
Warning message:
In checkConv(attr(opt, "derivs"), opt$par, ctrl = control$checkConv, : Model is nearly unidentifiable: very large eigenvalue
 - Rescale variables?
```

In [71]:

```
summary(m_poisson2)
```

Out[71]:

```
Generalized linear mixed model fit by maximum likelihood (Laplace
  Approximation) [glmerMod]
 Family: poisson  ( log )
Formula: body_length ~ 1 + session_index + session_comments + (1 | author) +  
    (1 | obs)
   Data: data

     AIC      BIC   logLik deviance df.resid 
 6237919  6237975 -3118954  6237909   515191 

Scaled residuals: 
     Min       1Q   Median       3Q      Max 
-1.64115 -0.08574  0.00234  0.04149  0.15658 

Random effects:
 Groups Name        Variance Std.Dev.
 obs    (Intercept) 1.0735   1.036   
 author (Intercept) 0.3237   0.569   
Number of obs: 515196, groups:  obs, 515196; author, 288629

Fixed effects:
                   Estimate Std. Error z value Pr(>|z|)    
(Intercept)       4.4548812  0.0034859  1278.0   <2e-16 ***
session_index    -0.0206626  0.0011955   -17.3   <2e-16 ***
session_comments  0.0179436  0.0009419    19.1   <2e-16 ***
---
Signif. codes:  0 ‘***’ 0.001 ‘**’ 0.01 ‘*’ 0.05 ‘.’ 0.1 ‘ ’ 1

Correlation of Fixed Effects:
            (Intr) sssn_n
session_ndx -0.167       
sssn_cmmnts -0.526 -0.639
convergence code: 0
Model is nearly unidentifiable: very large eigenvalue
 - Rescale variables?
```

In [72]:

```
overdisp_fun(m_poisson2)
```

Out[72]:

chisq
:   19332.9050753099

ratio
:   0.0375257042054498

rdf
:   515191

p
:   1

Great, the overdispersion problem is gone. The results (coefficients) are very similar to the previous models though (specifically to the log-normal).

For completeness, we also want to utilize a negative binomial family though by first using the nb functionality provided in lme4.

In [ ]:

```
m_nb = glmer.nb(body_length~1+session_index+session_comments+(1|author),data=data)
```

```
Warning message:
In checkConv(attr(opt, "derivs"), opt$par, ctrl = control$checkConv, : Model failed to converge with max|grad| = 0.00407952 (tol = 0.001, component 1)Warning message:
In checkConv(attr(opt, "derivs"), opt$par, ctrl = control$checkConv, : Model is nearly unidentifiable: very large eigenvalue
 - Rescale variables?Warning message:
In checkConv(attr(opt, "derivs"), opt$par, ctrl = control$checkConv, : Model failed to converge with max|grad| = 0.366738 (tol = 0.001, component 1)Warning message:
In checkConv(attr(opt, "derivs"), opt$par, ctrl = control$checkConv, : Model is nearly unidentifiable: very large eigenvalue
 - Rescale variables?Warning message:
In checkConv(attr(opt, "derivs"), opt$par, ctrl = control$checkConv, : Model failed to converge with max|grad| = 0.00397176 (tol = 0.001, component 1)Warning message:
In checkConv(attr(opt, "derivs"), opt$par, ctrl = control$checkConv, : Model is nearly unidentifiable: very large eigenvalue
 - Rescale variables?Warning message:
In checkConv(attr(opt, "derivs"), opt$par, ctrl = control$checkConv, : Model failed to converge with max|grad| = 0.0115897 (tol = 0.001, component 1)Warning message:
In checkConv(attr(opt, "derivs"), opt$par, ctrl = control$checkConv, : Model is nearly unidentifiable: very large eigenvalue
 - Rescale variables?Warning message:
In checkConv(attr(opt, "derivs"), opt$par, ctrl = control$checkConv, : Model failed to converge with max|grad| = 0.0276036 (tol = 0.001, component 1)Warning message:
In checkConv(attr(opt, "derivs"), opt$par, ctrl = control$checkConv, : Model is nearly unidentifiable: very large eigenvalue
 - Rescale variables?
```

In [105]:

```
summary(m_nb)
```

Out[105]:

```
Generalized linear mixed model fit by maximum likelihood (Laplace
  Approximation) [glmerMod]
 Family: Negative Binomial(1.2607)  ( log )
Formula: body_length ~ 1 + session_index + session_comments + (1 | author)
   Data: data

     AIC      BIC   logLik deviance df.resid 
 6256424  6256480 -3128207  6256414   515191 

Scaled residuals: 
    Min      1Q  Median      3Q     Max 
-1.1206 -0.6550 -0.2316  0.4070 13.3236 

Random effects:
 Groups Name        Variance Std.Dev.
 author (Intercept) 0.4134   0.643   
Number of obs: 515196, groups:  author, 288629

Fixed effects:
                   Estimate Std. Error z value Pr(>|z|)    
(Intercept)       4.7722006  0.0034437  1385.8   <2e-16 ***
session_index    -0.0227814  0.0011235   -20.3   <2e-16 ***
session_comments  0.0221241  0.0008964    24.7   <2e-16 ***
---
Signif. codes:  0 ‘***’ 0.001 ‘**’ 0.01 ‘*’ 0.05 ‘.’ 0.1 ‘ ’ 1

Correlation of Fixed Effects:
            (Intr) sssn_n
session_ndx -0.153       
sssn_cmmnts -0.512 -0.638
convergence code: 0
Model failed to converge with max|grad| = 0.00430912 (tol = 0.001, component 1)
Model is nearly unidentifiable: very large eigenvalue
 - Rescale variables?
```

We end up with multiple convergence warnings (not shown); the coefficients are quite similar to our previous observations though. Note that this model also takes a very long time to fit for this sample of 1 mio. data points; it does not really scale for larger data which in our case consists of more than 50 mio. data points.

As a consequence, let us next also fit a negative binomial regression, but this time we use quasi likelihood.

In [ ]:

```
#just for initialization
init = glm.nb(body_length~1+session_index+session_comments,data=data)
```

In [106]:

```
init$theta
```

Out[106]:

0.86141910645522

In [ ]:

```
m_pql_nb = glmmPQL(body_length~1+session_index+session_comments, random = list(~1 | author), data = data, family = negative.binomial(theta=init$theta))
```

In [107]:

```
summary(m_pql_nb)
```

Out[107]:

```
Linear mixed-effects model fit by maximum likelihood
 Data: data 
  AIC BIC logLik
   NA  NA     NA

Random effects:
 Formula: ~1 | author
        (Intercept)  Residual
StdDev:   0.5674362 0.8578411

Variance function:
 Structure: fixed weights
 Formula: ~invwt 
Fixed effects: body_length ~ 1 + session_index + session_comments 
                     Value   Std.Error     DF   t-value p-value
(Intercept)       4.793046 0.003166117 288628 1513.8561       0
session_index    -0.022682 0.001074639 226565  -21.1065       0
session_comments  0.022100 0.000849586 226565   26.0122       0
 Correlation: 
                 (Intr) sssn_n
session_index    -0.165       
session_comments -0.523 -0.637

Standardized Within-Group Residuals:
       Min         Q1        Med         Q3        Max 
-2.7901396 -0.6490876 -0.2448333  0.3938080 15.4216657 

Number of Observations: 515196
Number of Groups: 288629
```

Again, similar results (even though we receive some convergence warnings).

## Final model choice¶

Given our extensive analysis regarding the appropriate model for our data, we conclude that regardless of our model choice, the coefficients and subsequent inference are very similar. Nonetheless, we will proceed with a linear model on the log-transformed body\_length due to the appropriate diagnostic plots, the simplicity of the model and the high mean of body\_length.

## Significance of effects¶

Now that we have decided on a model, we want to extend our inference. First, we want to study the significance of the fixed effects as well as potential additional random effects.

There are several options for determining the significance of a fixed effect on the model. For example, a common approach is to use the ratio of the slope to its standard error and then use a t-test for determining its significance. Alternatively, one can also use F-tests for contrasting simpler to more complex models. Both methods require a specification of the degrees of freedom though which is not trivial in mixed models; methods such as the Satterthwaite approximation or Kenward-Roger approximation can be used for that task.
As stated in Baayen 2008, we can also directly use the t-statistics given to derive statistical significance. Because we have a huge dataset, a t-statistic above 2 can be regarded as significant at the 5\% level in a two-tailed test. You can derive this directly from the results above.

However, here, we follow an approach used for comparing models: the Bayesian Information Criterion (alternatively we could also work with AIC or LRT). Basically, the idea is to specify different (in this case nested) models that successively incorporate additional fixed or random effects and then compare their BIC scores. The lower the BIC, the better a model is (above a small threshold).

The baseline model we start with is:

body\_length = 1 + (1|author)

The reason why we incorporate the random effect for author from the beginning is that this is necessary based on the design of the experiment. Also, as pointed out in http://glmm.wikidot.com/faq, you should not compare the fit of a (g)lmer with one from a (g)lm.

Okay, so let us get the BIC for the baseline model.

In [ ]:

```
m1 = lmer(log(body_length)~1+(1|author), data = data, REML=FALSE)
```

In [108]:

```
summary(m1)
```

Out[108]:

```
Linear mixed model fit by maximum likelihood  ['lmerMod']
Formula: log(body_length) ~ 1 + (1 | author)
   Data: data

      AIC       BIC    logLik  deviance  df.resid 
1650321.4 1650354.9 -825157.7 1650315.4    515193 

Scaled residuals: 
    Min      1Q  Median      3Q     Max 
-4.7292 -0.5556  0.0381  0.6032  4.0935 

Random effects:
 Groups   Name        Variance Std.Dev.
 author   (Intercept) 0.330    0.5745  
 Residual             1.163    1.0786  
Number of obs: 515196, groups:  author, 288629

Fixed effects:
            Estimate Std. Error t value
(Intercept) 4.458131   0.001941    2296
```

Next, let us incorporate the index and session length individually.

In [ ]:

```
m2 = lmer(log(body_length)~1+session_index+(1|author), data = data, REML=FALSE)
```

In [109]:

```
summary(m2)
```

Out[109]:

```
Linear mixed model fit by maximum likelihood  ['lmerMod']
Formula: log(body_length) ~ 1 + session_index + (1 | author)
   Data: data

      AIC       BIC    logLik  deviance  df.resid 
1650279.3 1650324.0 -825135.7 1650271.3    515192 

Scaled residuals: 
    Min      1Q  Median      3Q     Max 
-4.7159 -0.5578  0.0370  0.6037  4.0808 

Random effects:
 Groups   Name        Variance Std.Dev.
 author   (Intercept) 0.330    0.5745  
 Residual             1.163    1.0785  
Number of obs: 515196, groups:  author, 288629

Fixed effects:
                Estimate Std. Error t value
(Intercept)    4.4737038  0.0030443  1469.5
session_index -0.0062794  0.0009456    -6.6

Correlation of Fixed Effects:
            (Intr)
session_ndx -0.770
```

In [111]:

```
m3 = lmer(log(body_length)~1+session_comments+(1|author), data = data, REML=FALSE)
summary(m3)
```

Out[111]:

```
Linear mixed model fit by maximum likelihood  ['lmerMod']
Formula: log(body_length) ~ 1 + session_comments + (1 | author)
   Data: data

      AIC       BIC    logLik  deviance  df.resid 
1650220.6 1650265.3 -825106.3 1650212.6    515192 

Scaled residuals: 
    Min      1Q  Median      3Q     Max 
-4.7530 -0.5562  0.0372  0.6030  4.1022 

Random effects:
 Groups   Name        Variance Std.Dev.
 author   (Intercept) 0.3302   0.5746  
 Residual             1.1629   1.0784  
Number of obs: 515196, groups:  author, 288629

Fixed effects:
                  Estimate Std. Error t value
(Intercept)      4.4282411  0.0035296  1254.6
session_comments 0.0075506  0.0007446    10.1

Correlation of Fixed Effects:
            (Intr)
sssn_cmmnts -0.835
```

Based on the lower BIC we can clearly see that the incorporation of the session\_index and session\_comments (separately) improve our model, thus, we make inference on it (also the t-value indicates high significance).

In [ ]:

```
m4 = lmer(log(body_length)~1+session_index+session_comments+(1|author), data = data, REML=FALSE)
```

In [110]:

```
summary(m4)
```

Out[110]:

```
Linear mixed model fit by maximum likelihood  ['lmerMod']
Formula: log(body_length) ~ 1 + session_index + session_comments + (1 |  
    author)
   Data: data

      AIC       BIC    logLik  deviance  df.resid 
1649931.4 1649987.2 -824960.7 1649921.4    515191 

Scaled residuals: 
    Min      1Q  Median      3Q     Max 
-4.7413 -0.5556  0.0374  0.6037  4.0714 

Random effects:
 Groups   Name        Variance Std.Dev.
 author   (Intercept) 0.3303   0.5747  
 Residual             1.1621   1.0780  
Number of obs: 515196, groups:  author, 288629

Fixed effects:
                   Estimate Std. Error t value
(Intercept)       4.4384597  0.0035791  1240.1
session_index    -0.0209770  0.0012290   -17.1
session_comments  0.0181105  0.0009679    18.7

Correlation of Fixed Effects:
            (Intr) sssn_n
session_ndx -0.167       
sssn_cmmnts -0.526 -0.639
```

As expected based on our initial empirical analysis in the paper, both the session index and session length are important effects to incorporate.

We stop at this point as our scope of interest is covered. However, one can arbitrarily extend the existing model.
For example, one might also argue that it is necessary to incorporate a random effect for the intercept based on different subreddits.

In [ ]:

```
m5 = lmer(log(body_length)~1+session_index+session_comments+(1|author)+(1|subreddit), data = data, REML=FALSE)
```

In [126]:

```
summary(m5)
```

Out[126]:

```
Linear mixed model fit by maximum likelihood  ['lmerMod']
Formula: log(body_length) ~ 1 + session_index + session_comments + (1 |  
    author) + (1 | subreddit)
   Data: data

      AIC       BIC    logLik  deviance  df.resid 
1608475.2 1608542.1 -804231.6 1608463.2    515190 

Scaled residuals: 
    Min      1Q  Median      3Q     Max 
-5.2603 -0.5574  0.0443  0.6101  5.0959 

Random effects:
 Groups    Name        Variance Std.Dev.
 author    (Intercept) 0.2067   0.4546  
 subreddit (Intercept) 0.2532   0.5032  
 Residual              1.1192   1.0579  
Number of obs: 515196, groups:  author, 288629; subreddit, 12041

Fixed effects:
                   Estimate Std. Error t value
(Intercept)       4.4609224  0.0074568   598.2
session_index    -0.0205173  0.0011854   -17.3
session_comments  0.0208530  0.0009335    22.3

Correlation of Fixed Effects:
            (Intr) sssn_n
session_ndx -0.077       
sssn_cmmnts -0.222 -0.639
```

Again, the BIC shows an improvement in the model. Yet, the inference on the fixed index effect does not change. For complexity reasons, we neglect the random effect for the subreddit for the complete data.

### Outliers¶

We also want to check for robustness when removing outliers.

In [ ]:

```
data_limit <- romr.fnc(m3, data, trim = 2.5)$data
```

In [ ]:

```
m4_limit = lmer(log(body_length)~1+session_index+session_comments+(1|author),data=data_limit, REML=FALSE)
```

In [112]:

```
summary(m4_limit)
```

Out[112]:

```
Linear mixed model fit by maximum likelihood  ['lmerMod']
Formula: log(body_length) ~ 1 + session_index + session_comments + (1 |  
    author)
   Data: data_limit

      AIC       BIC    logLik  deviance  df.resid 
1534980.4 1535036.0 -767485.2 1534970.4    506076 

Scaled residuals: 
     Min       1Q   Median       3Q      Max 
-3.02984 -0.59809  0.02065  0.61528  2.81522 

Random effects:
 Groups   Name        Variance Std.Dev.
 author   (Intercept) 0.3415   0.5844  
 Residual             0.9360   0.9675  
Number of obs: 506081, groups:  author, 285915

Fixed effects:
                   Estimate Std. Error t value
(Intercept)       4.4695738  0.0033227  1345.2
session_index    -0.0174504  0.0011308   -15.4
session_comments  0.0171682  0.0008932    19.2

Correlation of Fixed Effects:
            (Intr) sssn_n
session_ndx -0.166       
sssn_cmmnts -0.523 -0.637
```

Not much changed.

### Collinearity¶

We also want to check for multicollinearity in our model; some explanations and code for checking that in lme4 is provided in https://hlplab.wordpress.com/2011/02/24/diagnosing-collinearity-in-lme4/.

In [113]:

```
source("../mer-utils.R")
kappa.mer(m4)
max(vif.mer(m4))
```

Out[113]:

4.48954996169921

Out[113]:

1.69068454529362

Both the condition number and VIF show no reasons to be concerned about collinearity in this model.

### REML¶

Until now, we have fitted the models using ML as this allowed us to compare models with each other. To be sure regarding the consistency of our coefficient estimations, we will now also quickly fit the model with REML.

In [ ]:

```
m4_reml = lmer(log(body_length)~1+session_index+session_comments+(1|author), data = data, REML=TRUE)
```

In [114]:

```
summary(m4_reml)
```

Out[114]:

```
Linear mixed model fit by REML ['lmerMod']
Formula: log(body_length) ~ 1 + session_index + session_comments + (1 |  
    author)
   Data: data

REML criterion at convergence: 1649956

Scaled residuals: 
    Min      1Q  Median      3Q     Max 
-4.7413 -0.5556  0.0374  0.6037  4.0714 

Random effects:
 Groups   Name        Variance Std.Dev.
 author   (Intercept) 0.3303   0.5747  
 Residual             1.1621   1.0780  
Number of obs: 515196, groups:  author, 288629

Fixed effects:
                   Estimate Std. Error t value
(Intercept)       4.4384597  0.0035791  1240.1
session_index    -0.0209770  0.0012290   -17.1
session_comments  0.0181105  0.0009679    18.7

Correlation of Fixed Effects:
            (Intr) sssn_n
session_ndx -0.167       
sssn_cmmnts -0.526 -0.639
```

The results are exactly the same.

### Length as random effect¶

We have decided to incorporate the variation between different session lengths as a controlling covariate, however we might also incorporate it as a controlling random effect.

In [ ]:

```
m4_control = lmer(log(body_length)~1+session_index+(1|session_comments)+(1|author), data = data, REML=TRUE)
```

In [115]:

```
summary(m4_control)
```

Out[115]:

```
Linear mixed model fit by REML ['lmerMod']
Formula: log(body_length) ~ 1 + session_index + (1 | session_comments) +  
    (1 | author)
   Data: data

REML criterion at convergence: 1649885

Scaled residuals: 
    Min      1Q  Median      3Q     Max 
-4.7123 -0.5570  0.0369  0.6038  4.0592 

Random effects:
 Groups           Name        Variance Std.Dev.
 author           (Intercept) 0.3300   0.57443 
 session_comments (Intercept) 0.0017   0.04123 
 Residual                     1.1620   1.07798 
Number of obs: 515196, groups:  author, 288629; session_comments, 9

Fixed effects:
               Estimate Std. Error t value
(Intercept)    4.543408   0.014597  311.25
session_index -0.020563   0.001221  -16.84

Correlation of Fixed Effects:
            (Intr)
session_ndx -0.292
```

The results are similar.

## Convergence problems¶

Convergence warnings (which we partly also see for experiments on the complete data) are most likely false positives and do not effect the results. The gold standard approach to investigate this is to utilize different optimizers and compare the coefficients. If they are not similar, our inference is stable which is the case here. The code is based on Ben Bolkers code presented online: http://stackoverflow.com/questions/21344555/convergence-error-for-development-version-of-lme4

In [117]:

```
g0.bobyqa <- lmer(log(body_length)~1+session_index+session_comments+(1|author),
      data = data,
                   control=lmerControl(optimizer="bobyqa"))
g0.NM <- update(g0.bobyqa,control=lmerControl(optimizer="Nelder_Mead"))
library(optimx)
g0.nlminb <- update(g0.bobyqa,control=lmerControl(optimizer="optimx",
                              optCtrl=list(method="nlminb")))
g0.LBFGSB <- update(g0.bobyqa,control=lmerControl(optimizer="optimx",
                              optCtrl=list(method="L-BFGS-B")))
```

```
Error in control$optimizer == "none": comparison (1) is possible only for atomic and list types
```

```
Error in control$optimizer == "none": comparison (1) is possible only for atomic and list types
```

In [127]:

```
getpar <- function(x) c(getME(x,c("theta")),fixef(x))
modList <- list(bobyqa=g0.bobyqa,NM=g0.NM,nlminb=g0.nlminb,
                LBFGSB=g0.LBFGSB)
ctab <- sapply(modList,getpar)
library(reshape2)
mtab <- melt(ctab)
library(ggplot2)
theme_set(theme_bw())
ggplot(mtab,aes(x=Var2,y=value,colour=Var2))+
    geom_point()+facet_wrap(~Var1,scale="free")
```

## Randomized baseline data¶

In order to finally confirm our intuition and inference on the negative effect of the session index on the text length, we also study our randomized baseline data. We basically have two different randomized datasets: (i) new sessions based on randomized time differences and (ii) randomized positions inside existing sessions.

### Randomized sessions (time differences)¶

In [119]:

```
idx = data$session_comments_random > 1
data_random = data[idx,]
```

In [120]:

```
m1_random = lmer(log(body_length)~1+(1|author), data = data_random, REML=FALSE)
summary(m1_random)
```

Out[120]:

```
Linear mixed model fit by maximum likelihood  ['lmerMod']
Formula: log(body_length) ~ 1 + (1 | author)
   Data: data_random

      AIC       BIC    logLik  deviance  df.resid 
1243363.3 1243395.9 -621678.6 1243357.3    388335 

Scaled residuals: 
    Min      1Q  Median      3Q     Max 
-4.7249 -0.5587  0.0364  0.6030  3.8770 

Random effects:
 Groups   Name        Variance Std.Dev.
 author   (Intercept) 0.3258   0.5708  
 Residual             1.1647   1.0792  
Number of obs: 388338, groups:  author, 216454

Fixed effects:
            Estimate Std. Error t value
(Intercept) 4.460537   0.002235    1996
```

In [121]:

```
m2_random = lmer(log(body_length)~1+session_index_random+(1|author), data = data_random, REML=FALSE)
summary(m2_random)
```

Out[121]:

```
Linear mixed model fit by maximum likelihood  ['lmerMod']
Formula: log(body_length) ~ 1 + session_index_random + (1 | author)
   Data: data_random

      AIC       BIC    logLik  deviance  df.resid 
1243329.8 1243373.2 -621660.9 1243321.8    388334 

Scaled residuals: 
    Min      1Q  Median      3Q     Max 
-4.7616 -0.5606  0.0366  0.6034  3.8746 

Random effects:
 Groups   Name        Variance Std.Dev.
 author   (Intercept) 0.3251   0.5702  
 Residual             1.1651   1.0794  
Number of obs: 388338, groups:  author, 216454

Fixed effects:
                       Estimate Std. Error t value
(Intercept)           4.4701134  0.0027516    1625
session_index_random -0.0032723  0.0005486      -6

Correlation of Fixed Effects:
            (Intr)
sssn_ndx_rn -0.584
```

In [122]:

```
m3_random = lmer(log(body_length)~1+session_comments_random+(1|author), data = data_random, REML=FALSE)
summary(m3_random)
```

Out[122]:

```
Linear mixed model fit by maximum likelihood  ['lmerMod']
Formula: log(body_length) ~ 1 + session_comments_random + (1 | author)
   Data: data_random

      AIC       BIC    logLik  deviance  df.resid 
1243301.7 1243345.2 -621646.9 1243293.7    388334 

Scaled residuals: 
    Min      1Q  Median      3Q     Max 
-4.7622 -0.5602  0.0367  0.6035  3.8751 

Random effects:
 Groups   Name        Variance Std.Dev.
 author   (Intercept) 0.3247   0.5698  
 Residual             1.1653   1.0795  
Number of obs: 388338, groups:  author, 216454

Fixed effects:
                          Estimate Std. Error t value
(Intercept)              4.4744779  0.0028363    1578
session_comments_random -0.0028653  0.0003591      -8

Correlation of Fixed Effects:
            (Intr)
sssn_cmmnt_ -0.616
```

In [123]:

```
m4_random = lmer(log(body_length)~1+session_index_random+session_comments_random+(1|author), data = data_random, REML=FALSE)
summary(m4_random)
```

Out[123]:

```
Linear mixed model fit by maximum likelihood  ['lmerMod']
Formula: 
log(body_length) ~ 1 + session_index_random + session_comments_random +  
    (1 | author)
   Data: data_random

      AIC       BIC    logLik  deviance  df.resid 
1243303.7 1243358.0 -621646.9 1243293.7    388333 

Scaled residuals: 
    Min      1Q  Median      3Q     Max 
-4.7626 -0.5602  0.0367  0.6035  3.8751 

Random effects:
 Groups   Name        Variance Std.Dev.
 author   (Intercept) 0.3247   0.5698  
 Residual             1.1653   1.0795  
Number of obs: 388338, groups:  author, 216454

Fixed effects:
                          Estimate Std. Error t value
(Intercept)              4.475e+00  2.874e-03  1556.9
session_index_random    -7.055e-05  8.165e-04    -0.1
session_comments_random -2.831e-03  5.345e-04    -5.3

Correlation of Fixed Effects:
            (Intr) sssn_n_
sssn_ndx_rn -0.161        
sssn_cmmnt_ -0.289 -0.741
```

When comparing m3\_random and m4\_random, we can see now is that the incorporation of the session index is not improving the model in terms of the BIC score and is thus, not significant (also has a low t-value < 2).

The reason why the session length itself is still significant can be explained by our very conservative randomization method that just randomizes the time differences between comments before sessionizing and thus, still keeps many sessions intact.

### Randomized indexes¶

In [124]:

```
m3_random_2 = lmer(log(body_length)~1+session_comments+(1|author), data = data, REML=FALSE)
summary(m3_random_2)
```

Out[124]:

```
Linear mixed model fit by maximum likelihood  ['lmerMod']
Formula: log(body_length) ~ 1 + session_comments + (1 | author)
   Data: data

      AIC       BIC    logLik  deviance  df.resid 
1650220.6 1650265.3 -825106.3 1650212.6    515192 

Scaled residuals: 
    Min      1Q  Median      3Q     Max 
-4.7530 -0.5562  0.0372  0.6030  4.1022 

Random effects:
 Groups   Name        Variance Std.Dev.
 author   (Intercept) 0.3302   0.5746  
 Residual             1.1629   1.0784  
Number of obs: 515196, groups:  author, 288629

Fixed effects:
                  Estimate Std. Error t value
(Intercept)      4.4282411  0.0035296  1254.6
session_comments 0.0075506  0.0007446    10.1

Correlation of Fixed Effects:
            (Intr)
sssn_cmmnts -0.835
```

In [125]:

```
m4_random_2 = lmer(log(body_length)~1+session_index_random_2+session_comments+(1|author), data = data, REML=FALSE)
summary(m4_random_2)
```

Out[125]:

```
Linear mixed model fit by maximum likelihood  ['lmerMod']
Formula: log(body_length) ~ 1 + session_index_random_2 + session_comments +  
    (1 | author)
   Data: data

      AIC       BIC    logLik  deviance  df.resid 
1650222.6 1650278.4 -825106.3 1650212.6    515191 

Scaled residuals: 
    Min      1Q  Median      3Q     Max 
-4.7531 -0.5562  0.0372  0.6030  4.1022 

Random effects:
 Groups   Name        Variance Std.Dev.
 author   (Intercept) 0.3302   0.5746  
 Residual             1.1629   1.0784  
Number of obs: 515196, groups:  author, 288629

Fixed effects:
                         Estimate Std. Error t value
(Intercept)             4.428e+00  3.584e-03  1235.7
session_index_random_2 -7.037e-05  1.231e-03    -0.1
session_comments        7.586e-03  9.652e-04     7.9

Correlation of Fixed Effects:
            (Intr) ss___2
sssn_ndx__2 -0.173       
sssn_cmmnts -0.524 -0.636
```

Again, we see similar behavior, the incorporation of the random session index does not improve the model compared to one that already includes the session length.

Overall, the analyses presented in this notebook have demonstrated that the course of a session has a (negative) effect on the number of answers a post receives.

In [ ]:

```

```
