# Supplementary material for: Evidence of Online Performance Deterioration in User Sessions on Reddit
Source: PLoS One. 2016 Aug 25;11(8):e0161636. doi: 10.1371/journal.pone.0161636 (PMC4999233; doi:10.1371/journal.pone.0161636)
Supplement: S6 Notebook — (HTML) [file pone.0161636.s006.html]

dynamic\_num\_responses


In [43]:

```
library(lme4)
options(rgl.useNULL=TRUE)
library(LMERConvenienceFunctions)

library(ggplot2)
options(jupyter.plot_mimetypes = 'image/png')
library(repr)
options(repr.plot.width=6, repr.plot.height=6)

library(car)
library(MASS)

library(vcd)
```

# Number of answers¶

In this notebook, we document our various steps taken to study the effect of the course of a session on the number of answers a comment receives. Note that we only do this on a sample of 1 mio. data points here.

In [44]:

```
data = read.csv("/home/psinger/Reddit-depletion/data/sample.csv", header=TRUE)
```

In [45]:

```
nrow(data)
```

Out[45]:

1000000

## Data inspection¶

We start by getting a better feeling of our data. Note that num\_answers represents count data.

We can limit our data to those sessions that have at least two and less than 11 comments.

In [46]:

```
idx = data$session_comments > 1 & data$session_comments < 11
```

In [47]:

```
data = data[idx,]
```

In [48]:

```
nrow(data)
```

Out[48]:

515196

In [49]:

```
hist(data$num_answers)
```

In [50]:

```
hist(data$num_answers[data$num_answers<10])
```

## Regression model¶

We use mixed-effects models where in the basic form (that we analyze in this notebook), we are interested in studying num\_answers ~ 1 + session\_index + session\_comments + (1|author).

### Linear mixed-effects regression¶

Even though we cannot expect a simple linear mixed-effects regression to be an appropriate model for our count data, we start with it.

In [51]:

```
m_lmer = lmer(num_answers~1+session_index + session_comments+(1|author), data=data, REML=FALSE)
```

In [52]:

```
summary(m_lmer)
```

Out[52]:

```
Linear mixed model fit by maximum likelihood  ['lmerMod']
Formula: num_answers ~ 1 + session_index + session_comments + (1 | author)
   Data: data

      AIC       BIC    logLik  deviance  df.resid 
1823639.9 1823695.6 -911814.9 1823629.9    515191 

Scaled residuals: 
    Min      1Q  Median      3Q     Max 
 -3.054  -0.443  -0.399   0.260 200.394 

Random effects:
 Groups   Name        Variance Std.Dev.
 author   (Intercept) 0.02697  0.1642  
 Residual             1.99077  1.4109  
Number of obs: 515196, groups:  author, 288629

Fixed effects:
                  Estimate Std. Error t value
(Intercept)       0.619751   0.004167  148.72
session_index    -0.042064   0.001489  -28.25
session_comments  0.030654   0.001150   26.66

Correlation of Fixed Effects:
            (Intr) sssn_n
session_ndx -0.174       
sssn_cmmnts -0.538 -0.652
```

In [53]:

```
mcp.fnc(m_lmer)
```

What we can see in above plot is, that the residuals do not appear to be normally distributed and we can see clear heteroskedasticity. Thus, as expected, a linear model does not provide a good fit here.

### Linear mixed-effects regression (log-transform)¶

Next, let us take the log of the response variable and repeat a linear regression.

In [54]:

```
m_lmer_log = lmer(log1p(num_answers)~1+session_index+session_comments+(1|author), data=data, REML=FALSE)
```

In [55]:

```
summary(m_lmer_log)
```

Out[55]:

```
Linear mixed model fit by maximum likelihood  ['lmerMod']
Formula: log1p(num_answers) ~ 1 + session_index + session_comments + (1 |  
    author)
   Data: data

      AIC       BIC    logLik  deviance  df.resid 
 641073.8  641129.6 -320531.9  641063.8    515191 

Scaled residuals: 
    Min      1Q  Median      3Q     Max 
-1.4921 -0.7955 -0.7139  0.7173 11.6273 

Random effects:
 Groups   Name        Variance Std.Dev.
 author   (Intercept) 0.008545 0.09244 
 Residual             0.194979 0.44156 
Number of obs: 515196, groups:  author, 288629

Fixed effects:
                   Estimate Std. Error t value
(Intercept)       0.3557755  0.0013273  268.05
session_index    -0.0185186  0.0004719  -39.24
session_comments  0.0147068  0.0003657   40.22

Correlation of Fixed Effects:
            (Intr) sssn_n
session_ndx -0.174       
sssn_cmmnts -0.536 -0.649
```

In [56]:

```
mcp.fnc(m_lmer_log)
```

That does not look satisfying at all. We should focus on generalized mixed-effects models to better model the count data.

### Poisson GLMER¶

Let us start with a Poisson GLMER, which is a main choice when working with count data.

In [57]:

```
m_poisson = glmer(num_answers~1+session_index+session_comments+(1|author),data=data,family=poisson())
```

```
Warning message:
In checkConv(attr(opt, "derivs"), opt$par, ctrl = control$checkConv, : Model failed to converge with max|grad| = 0.00539401 (tol = 0.001, component 1)Warning message:
In checkConv(attr(opt, "derivs"), opt$par, ctrl = control$checkConv, : Model is nearly unidentifiable: very large eigenvalue
 - Rescale variables?
```

In [58]:

```
summary(m_poisson)
```

Out[58]:

```
Generalized linear mixed model fit by maximum likelihood (Laplace
  Approximation) [glmerMod]
 Family: poisson  ( log )
Formula: num_answers ~ 1 + session_index + session_comments + (1 | author)
   Data: data

      AIC       BIC    logLik  deviance  df.resid 
1119602.2 1119646.8 -559797.1 1119594.2    515192 

Scaled residuals: 
   Min     1Q Median     3Q    Max 
-8.708 -0.658 -0.591  0.554 44.510 

Random effects:
 Groups Name        Variance Std.Dev.
 author (Intercept) 0.398    0.6308  
Number of obs: 515196, groups:  author, 288629

Fixed effects:
                  Estimate Std. Error z value Pr(>|z|)    
(Intercept)      -0.695957   0.004442 -156.69   <2e-16 ***
session_index    -0.062017   0.001416  -43.80   <2e-16 ***
session_comments  0.045746   0.001078   42.42   <2e-16 ***
---
Signif. codes:  0 ‘***’ 0.001 ‘**’ 0.01 ‘*’ 0.05 ‘.’ 0.1 ‘ ’ 1

Correlation of Fixed Effects:
            (Intr) sssn_n
session_ndx -0.183       
sssn_cmmnts -0.513 -0.605
convergence code: 0
Model failed to converge with max|grad| = 0.00539401 (tol = 0.001, component 1)
Model is nearly unidentifiable: very large eigenvalue
 - Rescale variables?
```

In [59]:

```
plot(fitted(m_poisson),resid(m_poisson))
```

The convergence message shows low magnitude and for such large data this might be a false positive as emphasized in the lme4 docu.

The residual plots for generalized linear models are generally quite difficult to interpret as the residuals do not necessarily need to follow a normal distribution any longer. However, the fitted vs. residual plots shows a typical pattern for Poisson regression with curvilinear traces. What we can do though as a first step here, is to look at overdispersion (see http://glmm.wikidot.com/faq).

In [60]:

```
overdisp_fun <- function(model) {
  ## number of variance parameters in 
  ##   an n-by-n variance-covariance matrix
  vpars <- function(m) {
    nrow(m)*(nrow(m)+1)/2
  }
  model.df <- sum(sapply(VarCorr(model),vpars))+length(fixef(model))
  rdf <- nrow(model.frame(model))-model.df
  rp <- residuals(model,type="pearson")
  Pearson.chisq <- sum(rp^2)
  prat <- Pearson.chisq/rdf
  pval <- pchisq(Pearson.chisq, df=rdf, lower.tail=FALSE)
  c(chisq=Pearson.chisq,ratio=prat,rdf=rdf,p=pval)
}
```

In [61]:

```
overdisp_fun(m_poisson)
```

Out[61]:

chisq
:   408930.369865232

ratio
:   0.793743633179926

rdf
:   515192

p
:   1

It does not seem that we have any problem with overdispersion here.

Let us be sure and check it with the dispersion\_glmer function from the blmeco packages.

In [62]:

```
library("blmeco") 
dispersion_glmer(m_poisson)
```

Out[62]:

1.00794778266311

The value is between 0.75 and 1.4 which gives an indicator that there is no overdispersion problem.

What might be an issue for our model though, is the vast presence of zeros in our data. In order to tackle this issue, one can look at zero-inflated Poisson models. Unfortunately, these are only implemented in the MCMCglmm and glmmADMB packages which both utilize complex fitting methods that do not scale to large data and are thus, not useful for our large data case.

To that end, we use a generic "implementation of the EM algorithm that alternates between fitting a GLMM with data that are weighted according to their zero probability, and fitting a binary FLM for the probability that a data point is zero"---for detailed reference please refer to https://groups.nceas.ucsb.edu/non-linear-modeling/projects/owls/WRITEUP/owls.pdf.

In [63]:

```
source("../owls_R_funs.R")
```

In [ ]:

```
m_poisson_zi = zipme(cformula=num_answers~1+session_index+session_comments+(1|author), zformula=z~1,data=data,cfamily=poisson())
```

```
itr: 1
```

```
Warning message:
In checkConv(attr(opt, "derivs"), opt$par, ctrl = control$checkConv, : Model is nearly unidentifiable: very large eigenvalue
 - Rescale variables?
```

```
itr: 2
```

```
Warning message:
In checkConv(attr(opt, "derivs"), opt$par, ctrl = control$checkConv, : Model is nearly unidentifiable: very large eigenvalue
 - Rescale variables?
```

```
itr: 3
```

```
Warning message:
In checkConv(attr(opt, "derivs"), opt$par, ctrl = control$checkConv, : Model failed to converge with max|grad| = 0.00320821 (tol = 0.001, component 1)Warning message:
In checkConv(attr(opt, "derivs"), opt$par, ctrl = control$checkConv, : Model is nearly unidentifiable: very large eigenvalue
 - Rescale variables?
```

```
itr: 4
```

```
Warning message:
In checkConv(attr(opt, "derivs"), opt$par, ctrl = control$checkConv, : Model is nearly unidentifiable: very large eigenvalue
 - Rescale variables?
```

```
itr: 5
```

```
Warning message:
In checkConv(attr(opt, "derivs"), opt$par, ctrl = control$checkConv, : Model failed to converge with max|grad| = 0.0010158 (tol = 0.001, component 1)Warning message:
In checkConv(attr(opt, "derivs"), opt$par, ctrl = control$checkConv, : Model is nearly unidentifiable: very large eigenvalue
 - Rescale variables?
```

```
itr: 6
```

```
Warning message:
In checkConv(attr(opt, "derivs"), opt$par, ctrl = control$checkConv, : Model failed to converge with max|grad| = 0.00337835 (tol = 0.001, component 1)Warning message:
In checkConv(attr(opt, "derivs"), opt$par, ctrl = control$checkConv, : Model is nearly unidentifiable: very large eigenvalue
 - Rescale variables?
```

```
itr: 7
```

```
Warning message:
In checkConv(attr(opt, "derivs"), opt$par, ctrl = control$checkConv, : Model failed to converge with max|grad| = 0.0018187 (tol = 0.001, component 1)Warning message:
In checkConv(attr(opt, "derivs"), opt$par, ctrl = control$checkConv, : Model is nearly unidentifiable: very large eigenvalue
 - Rescale variables?
```

```
itr: 8
```

```
Warning message:
In checkConv(attr(opt, "derivs"), opt$par, ctrl = control$checkConv, : Model failed to converge with max|grad| = 0.0019164 (tol = 0.001, component 1)Warning message:
In checkConv(attr(opt, "derivs"), opt$par, ctrl = control$checkConv, : Model is nearly unidentifiable: very large eigenvalue
 - Rescale variables?
```

In [87]:

```
summary(m_poisson_zi)
```

Out[87]:

```
          Length Class    Mode   
zfit          30 glm      list   
cfit           1 glmerMod S4     
itr            1 -none-   numeric
deltainfo     20 -none-   numeric
z         515196 -none-   numeric
```

In [88]:

```
summary(m_poisson_zi$cfit)
```

Out[88]:

```
Generalized linear mixed model fit by maximum likelihood (Laplace
  Approximation) [glmerMod]
 Family: poisson  ( log )
Formula: num_answers ~ 1 + session_index + session_comments + (1 | author)
   Data: bydataw
Weights: (1 - z)

      AIC       BIC    logLik  deviance  df.resid 
1086528.5 1086573.1 -543260.2 1086520.5    515192 

Scaled residuals: 
     Min       1Q   Median       3Q      Max 
-11.7907  -0.6490  -0.5860   0.5147  21.4109 

Random effects:
 Groups Name        Variance Std.Dev.
 author (Intercept) 0.3912   0.6254  
Number of obs: 515196, groups:  author, 288629

Fixed effects:
                  Estimate Std. Error z value Pr(>|z|)    
(Intercept)      -0.651175   0.004437 -146.76   <2e-16 ***
session_index    -0.060732   0.001419  -42.80   <2e-16 ***
session_comments  0.044971   0.001082   41.57   <2e-16 ***
---
Signif. codes:  0 ‘***’ 0.001 ‘**’ 0.01 ‘*’ 0.05 ‘.’ 0.1 ‘ ’ 1

Correlation of Fixed Effects:
            (Intr) sssn_n
session_ndx -0.183       
sssn_cmmnts -0.514 -0.607
convergence code: 0
Model failed to converge with max|grad| = 0.00108068 (tol = 0.001, component 1)
Model is nearly unidentifiable: very large eigenvalue
 - Rescale variables?
```

Even though we see some convergence warnings (low magnitude, for each iteration), we can see is that the coefficients are very similar to the model fitted without zero-inflation. Comparing the BIC thoughs reveals a better fit in general for the ZIP.

Again, for completeness, we fit a negative binomial regression with quasi likelihood.

In [ ]:

```
#just for initialization
init = glm.nb(num_answers~1+session_index+session_comments,data=data)
```

In [89]:

```
init$theta
```

Out[89]:

1.63717013177077

In [ ]:

```
m_pql_nb = glmmPQL(num_answers~1+session_index+session_comments, random = list(~1 | author), data = data, family = negative.binomial(theta=init$theta))
```

```
iteration 1
iteration 2
iteration 3
iteration 4
iteration 5
iteration 6
iteration 7
iteration 8
iteration 9
iteration 10
```

In [108]:

```
summary(m_pql_nb)
```

Out[108]:

```
Linear mixed-effects model fit by maximum likelihood
 Data: data 
  AIC BIC logLik
   NA  NA     NA

Random effects:
 Formula: ~1 | author
        (Intercept)  Residual
StdDev:   0.5794954 0.8185763

Variance function:
 Structure: fixed weights
 Formula: ~invwt 
Fixed effects: num_answers ~ 1 + session_index + session_comments 
                      Value   Std.Error     DF    t-value p-value
(Intercept)      -0.6119775 0.004101172 288628 -149.22014       0
session_index    -0.0575931 0.001397287 226565  -41.21776       0
session_comments  0.0434079 0.001071905 226565   40.49599       0
 Correlation: 
                 (Intr) sssn_n
session_index    -0.185       
session_comments -0.536 -0.618

Standardized Within-Group Residuals:
       Min         Q1        Med         Q3        Max 
-4.0031806 -0.7304668 -0.6600525  0.5278735 23.7386414 

Number of Observations: 515196
Number of Groups: 288629
```

Again, very similar results.

## Final model choice¶

Given our extensive analysis regarding the appropriate model for our data, we can conclude that all have very similar conclusions, namely that the session\_index has a negative effect on num\_answers. Probably, the most appropriate model would be the zero-inflated Poisson regression. However, as the simple Poisson regression scales much better for larger data, we will continue to use it from this point on. Also, we do not use quasi likelihood, as it is difficult to compare the significance of fixed and random effects as no likelihood can be given.

## Significance of effects¶

Now that we have decided on a model, we want to extend our inference. First, we want to study the significance of the fixed effects as well as potential additional random effects.

There are several options for determining the significance of a fixed effect on the model. For example, a common approach is to use the ratio of the slope to its standard error and then use a t-test for determining its significance. Alternatively, one can also use F-tests for contrasting simpler to more complex models. Both methods require a specification of the degrees of freedom though which is not trivial in mixed models; methods such as the Satterthwaite approximation or Kenward-Roger approximation can be used for that task.
As stated in Baayen 2008, we can also directly use the t-statistics given to derive statistical significance. Because we have a huge dataset, a t-statistic above 2 can be regarded as significant at the 5\% level in a two-tailed test. You can derive this directly from the results above.

However, here, we follow an approach used for comparing models: the Bayesian Information Criterion (alternatively we could also work with AIC or LRT). Basically, the idea is to specify different (in this case nested) models that successively incorporate additional fixed or random effects and then compare their BIC scores. The lower the BIC, the better a model is (above a small threshold).

The baseline model we start with is:

num\_answers = 1 + (1|author)

The reason why we incorporate the random effect for author from the beginning is that this is necessary based on the design of the experiment. Also, as pointed out in http://glmm.wikidot.com/faq, you should not compare the fit of a (g)lmer with one from a (g)lm.

Okay, so let us get the BIC for the baseline model.

In [91]:

```
m1 = glmer(num_answers~1+(1|author), data = data, family=poisson())
summary(m1)
```

Out[91]:

```
Generalized linear mixed model fit by maximum likelihood (Laplace
  Approximation) [glmerMod]
 Family: poisson  ( log )
Formula: num_answers ~ 1 + (1 | author)
   Data: data

      AIC       BIC    logLik  deviance  df.resid 
1121866.2 1121888.5 -560931.1 1121862.2    515194 

Scaled residuals: 
   Min     1Q Median     3Q    Max 
-8.647 -0.657 -0.584  0.512 41.656 

Random effects:
 Groups Name        Variance Std.Dev.
 author (Intercept) 0.4012   0.6334  
Number of obs: 515196, groups:  author, 288629

Fixed effects:
             Estimate Std. Error z value Pr(>|z|)    
(Intercept) -0.666373   0.002642  -252.3   <2e-16 ***
---
Signif. codes:  0 ‘***’ 0.001 ‘**’ 0.01 ‘*’ 0.05 ‘.’ 0.1 ‘ ’ 1
```

Next, let us incorporate the index and session length individually.

In [92]:

```
m2 = glmer(num_answers~1+session_index+(1|author), data = data, family=poisson())
summary(m2)
```

Out[92]:

```
Generalized linear mixed model fit by maximum likelihood (Laplace
  Approximation) [glmerMod]
 Family: poisson  ( log )
Formula: num_answers ~ 1 + session_index + (1 | author)
   Data: data

      AIC       BIC    logLik  deviance  df.resid 
1121353.7 1121387.1 -560673.8 1121347.7    515193 

Scaled residuals: 
   Min     1Q Median     3Q    Max 
-8.588 -0.664 -0.592  0.524 41.917 

Random effects:
 Groups Name        Variance Std.Dev.
 author (Intercept) 0.3994   0.632   
Number of obs: 515196, groups:  author, 288629

Fixed effects:
               Estimate Std. Error z value Pr(>|z|)    
(Intercept)   -0.603266   0.003816 -158.07   <2e-16 ***
session_index -0.025455   0.001130  -22.52   <2e-16 ***
---
Signif. codes:  0 ‘***’ 0.001 ‘**’ 0.01 ‘*’ 0.05 ‘.’ 0.1 ‘ ’ 1

Correlation of Fixed Effects:
            (Intr)
session_ndx -0.722
convergence code: 0
Model is nearly unidentifiable: very large eigenvalue
 - Rescale variables?
```

In [93]:

```
m3 = glmer(num_answers~1+session_comments+(1|author), data = data, family=poisson())
summary(m3)
```

Out[93]:

```
Generalized linear mixed model fit by maximum likelihood (Laplace
  Approximation) [glmerMod]
 Family: poisson  ( log )
Formula: num_answers ~ 1 + session_comments + (1 | author)
   Data: data

      AIC       BIC    logLik  deviance  df.resid 
1121525.0 1121558.4 -560759.5 1121519.0    515193 

Scaled residuals: 
   Min     1Q Median     3Q    Max 
-8.719 -0.657 -0.598  0.539 42.410 

Random effects:
 Groups Name        Variance Std.Dev.
 author (Intercept) 0.4018   0.6339  
Number of obs: 515196, groups:  author, 288629

Fixed effects:
                  Estimate Std. Error z value Pr(>|z|)    
(Intercept)      -0.730513   0.004365 -167.34   <2e-16 ***
session_comments  0.015978   0.000858   18.62   <2e-16 ***
---
Signif. codes:  0 ‘***’ 0.001 ‘**’ 0.01 ‘*’ 0.05 ‘.’ 0.1 ‘ ’ 1

Correlation of Fixed Effects:
            (Intr)
sssn_cmmnts -0.796
convergence code: 0
Model failed to converge with max|grad| = 0.00474022 (tol = 0.001, component 1)
Model is nearly unidentifiable: very large eigenvalue
 - Rescale variables?
```

Based on the lower BIC we can clearly see that the incorporation of the session\_index and session\_comments (separately) improve our model, thus, we make inference on it (also the t-value indicates high significance).

In [94]:

```
m4 = glmer(num_answers~1+session_index+session_comments+(1|author), data = data, family=poisson())
summary(m4)
```

Out[94]:

```
Generalized linear mixed model fit by maximum likelihood (Laplace
  Approximation) [glmerMod]
 Family: poisson  ( log )
Formula: num_answers ~ 1 + session_index + session_comments + (1 | author)
   Data: data

      AIC       BIC    logLik  deviance  df.resid 
1119602.2 1119646.8 -559797.1 1119594.2    515192 

Scaled residuals: 
   Min     1Q Median     3Q    Max 
-8.708 -0.658 -0.591  0.554 44.510 

Random effects:
 Groups Name        Variance Std.Dev.
 author (Intercept) 0.398    0.6308  
Number of obs: 515196, groups:  author, 288629

Fixed effects:
                  Estimate Std. Error z value Pr(>|z|)    
(Intercept)      -0.695957   0.004442 -156.69   <2e-16 ***
session_index    -0.062017   0.001416  -43.80   <2e-16 ***
session_comments  0.045746   0.001078   42.42   <2e-16 ***
---
Signif. codes:  0 ‘***’ 0.001 ‘**’ 0.01 ‘*’ 0.05 ‘.’ 0.1 ‘ ’ 1

Correlation of Fixed Effects:
            (Intr) sssn_n
session_ndx -0.183       
sssn_cmmnts -0.513 -0.605
convergence code: 0
Model failed to converge with max|grad| = 0.00539401 (tol = 0.001, component 1)
Model is nearly unidentifiable: very large eigenvalue
 - Rescale variables?
```

As expected based on our initial empirical analysis in the paper, both the session index and session length are important effect to incorporate.

We stop at this point as our scope of interest is covered. However, one can arbitrarily extend the existing model.
For example, one might also argue that it is necessary to incorporate a random effect for the intercept based on different subreddits.

In [95]:

```
m5 = glmer(num_answers~1+session_index+session_comments+(1|author)+(1|subreddit), data = data, family=poisson())
summary(m5)
```

Out[95]:

```
Generalized linear mixed model fit by maximum likelihood (Laplace
  Approximation) [glmerMod]
 Family: poisson  ( log )
Formula: num_answers ~ 1 + session_index + session_comments + (1 | author) +  
    (1 | subreddit)
   Data: data

      AIC       BIC    logLik  deviance  df.resid 
1117926.1 1117981.9 -558958.1 1117916.1    515191 

Scaled residuals: 
   Min     1Q Median     3Q    Max 
-8.817 -0.691 -0.600  0.497 45.886 

Random effects:
 Groups    Name        Variance Std.Dev.
 author    (Intercept) 0.36087  0.6007  
 subreddit (Intercept) 0.01945  0.1395  
Number of obs: 515196, groups:  author, 288629; subreddit, 12041

Fixed effects:
                  Estimate Std. Error z value Pr(>|z|)    
(Intercept)      -0.725504   0.005606 -129.41   <2e-16 ***
session_index    -0.061710   0.001411  -43.73   <2e-16 ***
session_comments  0.044560   0.001078   41.35   <2e-16 ***
---
Signif. codes:  0 ‘***’ 0.001 ‘**’ 0.01 ‘*’ 0.05 ‘.’ 0.1 ‘ ’ 1

Correlation of Fixed Effects:
            (Intr) sssn_n
session_ndx -0.145       
sssn_cmmnts -0.384 -0.602
convergence code: 0
Model is nearly unidentifiable: very large eigenvalue
 - Rescale variables?
```

Again, the BIC shows an improvement in the model. Yet, the inference on the fixed effects does not change. For complexity reasons, we neglect the random effect for the subreddit for the complete data.

### Collinearity¶

We also want to check for multicollinearity in our model; some explanations and code for checking that in lme4 is provided in https://hlplab.wordpress.com/2011/02/24/diagnosing-collinearity-in-lme4/.

In [96]:

```
source("../mer-utils.R")
kappa.mer(m4)
max(vif.mer(m4))
```

Out[96]:

4.48954996169921

Out[96]:

1.57796040588551

Both the condition number and VIF show no reasons to be concerned about collinearity in this model.

### Length as random effect¶

We have decided to incorporate the variation between different session lengths as a controlling covariate, however we might also incorporate it as a controlling random effect.

In [97]:

```
m4_control = glmer(num_answers~1+session_index+(1|session_comments)+(1|author), data = data, family=poisson())
summary(m4_control)
```

Out[97]:

```
Generalized linear mixed model fit by maximum likelihood (Laplace
  Approximation) [glmerMod]
 Family: poisson  ( log )
Formula: num_answers ~ 1 + session_index + (1 | session_comments) + (1 |  
    author)
   Data: data

      AIC       BIC    logLik  deviance  df.resid 
1121355.8 1121400.4 -560673.9 1121347.8    515192 

Scaled residuals: 
   Min     1Q Median     3Q    Max 
-8.588 -0.664 -0.592  0.524 41.917 

Random effects:
 Groups           Name        Variance Std.Dev.
 author           (Intercept) 0.3994   0.632   
 session_comments (Intercept) 0.0000   0.000   
Number of obs: 515196, groups:  author, 288629; session_comments, 9

Fixed effects:
               Estimate Std. Error z value Pr(>|z|)    
(Intercept)   -0.603261   0.003817 -158.03   <2e-16 ***
session_index -0.025456   0.001130  -22.52   <2e-16 ***
---
Signif. codes:  0 ‘***’ 0.001 ‘**’ 0.01 ‘*’ 0.05 ‘.’ 0.1 ‘ ’ 1

Correlation of Fixed Effects:
            (Intr)
session_ndx -0.722
convergence code: 0
Model is nearly unidentifiable: very large eigenvalue
 - Rescale variables?
```

The coefficient also indicates a negative effect (but lower effect size).

## Convergence problems¶

Convergence warnings (which we partly also see for experiments on the complete data) are most likely false positives and do not effect the results. The gold standard approach to investigate this is to utilize different optimizers and compare the coefficients. If they are not similar, our inference is stable which is the case here. The code is based on Ben Bolkers code presented online: http://stackoverflow.com/questions/21344555/convergence-error-for-development-version-of-lme4

In [105]:

```
g0.bobyqa <- glmer(num_answers~1+session_index+session_comments+(1|author),
      family = poisson, data = data,
                   control=glmerControl(optimizer="bobyqa"))
g0.NM <- update(g0.bobyqa,control=glmerControl(optimizer="Nelder_Mead"))
library(optimx)
g0.nlminb <- update(g0.bobyqa,control=glmerControl(optimizer="optimx",
                              optCtrl=list(method="nlminb")))
g0.LBFGSB <- update(g0.bobyqa,control=glmerControl(optimizer="optimx",
                              optCtrl=list(method="L-BFGS-B")))

library(nloptr)
## from https://github.com/lme4/lme4/issues/98:
defaultControl <- list(algorithm="NLOPT_LN_BOBYQA",xtol_rel=1e-6,maxeval=1e5)
nloptwrap2 <- function(fn,par,lower,upper,control=list(),...) {
    for (n in names(defaultControl)) 
      if (is.null(control[[n]])) control[[n]] <- defaultControl[[n]]
    res <- nloptr(x0=par,eval_f=fn,lb=lower,ub=upper,opts=control,...)
    with(res,list(par=solution,
                  fval=objective,
                  feval=iterations,
                  conv=if (status>0) 0 else status,
                  message=message))
}
g0.bobyqa2 <- update(g0.bobyqa,control=glmerControl(optimizer=nloptwrap2))
g0.NM2 <- update(g0.bobyqa,control=glmerControl(optimizer=nloptwrap2,
                           optCtrl=list(algorithm="NLOPT_LN_NELDERMEAD")))
```

```
Warning message:
In checkConv(attr(opt, "derivs"), opt$par, ctrl = control$checkConv, : Model is nearly unidentifiable: very large eigenvalue
 - Rescale variables?Warning message:
In checkConv(attr(opt, "derivs"), opt$par, ctrl = control$checkConv, : Model failed to converge with max|grad| = 0.00283729 (tol = 0.001, component 1)Warning message:
In checkConv(attr(opt, "derivs"), opt$par, ctrl = control$checkConv, : Model is nearly unidentifiable: very large eigenvalue
 - Rescale variables?Warning message:
In checkConv(attr(opt, "derivs"), opt$par, ctrl = control$checkConv, : Model is nearly unidentifiable: very large eigenvalue
 - Rescale variables?Warning message:
In checkConv(attr(opt, "derivs"), opt$par, ctrl = control$checkConv, : Model failed to converge with max|grad| = 0.105164 (tol = 0.001, component 1)Warning message:
In checkConv(attr(opt, "derivs"), opt$par, ctrl = control$checkConv, : Model is nearly unidentifiable: very large eigenvalue
 - Rescale variables?Warning message:
In checkConv(attr(opt, "derivs"), opt$par, ctrl = control$checkConv, : Model failed to converge with max|grad| = 0.00586488 (tol = 0.001, component 1)Warning message:
In checkConv(attr(opt, "derivs"), opt$par, ctrl = control$checkConv, : Model is nearly unidentifiable: very large eigenvalue
 - Rescale variables?Warning message:
In checkConv(attr(opt, "derivs"), opt$par, ctrl = control$checkConv, : Model is nearly unidentifiable: very large eigenvalue
 - Rescale variables?
```

In [109]:

```
getpar <- function(x) c(getME(x,c("theta")),fixef(x))
modList <- list(bobyqa=g0.bobyqa,NM=g0.NM,nlminb=g0.nlminb,
                bobyqa2=g0.bobyqa2,NM2=g0.NM2,LBFGSB=g0.LBFGSB)
ctab <- sapply(modList,getpar)
library(reshape2)
mtab <- melt(ctab)
library(ggplot2)
theme_set(theme_bw())
ggplot(mtab,aes(x=Var2,y=value,colour=Var2))+
    geom_point()+facet_wrap(~Var1,scale="free")
```

## Randomized baseline data¶

In order to finally confirm our intuition and inference on the negative effect of the session index on the number of responses, we also study our randomized baseline data. We basically have two different randomized datasets: (i) new sessions based on randomized time differences and (ii) randomized positions inside existing sessions.

In [ ]:

```
idx = data$session_comments_random > 1
data_random = data[idx,]
```

In [99]:

```
m1_random = glmer(num_answers~1+(1|author), data = data_random, family=poisson())
summary(m1_random)
```

Out[99]:

```
Generalized linear mixed model fit by maximum likelihood (Laplace
  Approximation) [glmerMod]
 Family: poisson  ( log )
Formula: num_answers ~ 1 + (1 | author)
   Data: data_random

      AIC       BIC    logLik  deviance  df.resid 
 846628.8  846650.5 -423312.4  846624.8    388336 

Scaled residuals: 
   Min     1Q Median     3Q    Max 
-9.559 -0.657 -0.584  0.511 41.652 

Random effects:
 Groups Name        Variance Std.Dev.
 author (Intercept) 0.4025   0.6344  
Number of obs: 388338, groups:  author, 216454

Fixed effects:
             Estimate Std. Error z value Pr(>|z|)    
(Intercept) -0.664973   0.003042  -218.6   <2e-16 ***
---
Signif. codes:  0 ‘***’ 0.001 ‘**’ 0.01 ‘*’ 0.05 ‘.’ 0.1 ‘ ’ 1
```

In [100]:

```
m2_random = glmer(num_answers~1+session_index_random+(1|author), data = data_random, family=poisson())
summary(m2_random)
```

Out[100]:

```
Generalized linear mixed model fit by maximum likelihood (Laplace
  Approximation) [glmerMod]
 Family: poisson  ( log )
Formula: num_answers ~ 1 + session_index_random + (1 | author)
   Data: data_random

      AIC       BIC    logLik  deviance  df.resid 
 846630.0  846662.6 -423312.0  846624.0    388335 

Scaled residuals: 
   Min     1Q Median     3Q    Max 
-9.561 -0.657 -0.584  0.512 41.531 

Random effects:
 Groups Name        Variance Std.Dev.
 author (Intercept) 0.4026   0.6345  
Number of obs: 388338, groups:  author, 216454

Fixed effects:
                       Estimate Std. Error z value Pr(>|z|)    
(Intercept)          -0.6667287  0.0035875 -185.85   <2e-16 ***
session_index_random  0.0005890  0.0006369    0.92    0.355    
---
Signif. codes:  0 ‘***’ 0.001 ‘**’ 0.01 ‘*’ 0.05 ‘.’ 0.1 ‘ ’ 1

Correlation of Fixed Effects:
            (Intr)
sssn_ndx_rn -0.529
convergence code: 0
Model is nearly unidentifiable: very large eigenvalue
 - Rescale variables?
```

In [101]:

```
m3_random = glmer(num_answers~1+session_comments_random+(1|author), data = data_random, family=poisson())
summary(m3_random)
```

Out[101]:

```
Generalized linear mixed model fit by maximum likelihood (Laplace
  Approximation) [glmerMod]
 Family: poisson  ( log )
Formula: num_answers ~ 1 + session_comments_random + (1 | author)
   Data: data_random

      AIC       BIC    logLik  deviance  df.resid 
 846612.7  846645.3 -423303.3  846606.7    388335 

Scaled residuals: 
   Min     1Q Median     3Q    Max 
-9.565 -0.657 -0.587  0.515 41.162 

Random effects:
 Groups Name        Variance Std.Dev.
 author (Intercept) 0.4029   0.6347  
Number of obs: 388338, groups:  author, 216454

Fixed effects:
                          Estimate Std. Error z value Pr(>|z|)    
(Intercept)             -0.6736861  0.0036617  -184.0  < 2e-16 ***
session_comments_random  0.0017494  0.0004071     4.3 1.73e-05 ***
---
Signif. codes:  0 ‘***’ 0.001 ‘**’ 0.01 ‘*’ 0.05 ‘.’ 0.1 ‘ ’ 1

Correlation of Fixed Effects:
            (Intr)
sssn_cmmnt_ -0.557
convergence code: 0
Model failed to converge with max|grad| = 0.00176358 (tol = 0.001, component 1)
Model is nearly unidentifiable: very large eigenvalue
 - Rescale variables?
```

In [102]:

```
m4_random = glmer(num_answers~1+session_index_random+session_comments_random+(1|author), data = data_random, family=poisson())
summary(m4_random)
```

Out[102]:

```
Generalized linear mixed model fit by maximum likelihood (Laplace
  Approximation) [glmerMod]
 Family: poisson  ( log )
Formula: num_answers ~ 1 + session_index_random + session_comments_random +  
    (1 | author)
   Data: data_random

      AIC       BIC    logLik  deviance  df.resid 
 846604.1  846647.6 -423298.1  846596.1    388334 

Scaled residuals: 
   Min     1Q Median     3Q    Max 
-9.560 -0.657 -0.587  0.516 41.382 

Random effects:
 Groups Name        Variance Std.Dev.
 author (Intercept) 0.4028   0.6347  
Number of obs: 388338, groups:  author, 216454

Fixed effects:
                          Estimate Std. Error z value Pr(>|z|)    
(Intercept)             -0.6717834  0.0037117 -180.99  < 2e-16 ***
session_index_random    -0.0030156  0.0009276   -3.25  0.00115 ** 
session_comments_random  0.0031729  0.0005920    5.36 8.34e-08 ***
---
Signif. codes:  0 ‘***’ 0.001 ‘**’ 0.01 ‘*’ 0.05 ‘.’ 0.1 ‘ ’ 1

Correlation of Fixed Effects:
            (Intr) sssn_n_
sssn_ndx_rn -0.165        
sssn_cmmnt_ -0.256 -0.728 
convergence code: 0
Model is nearly unidentifiable: very large eigenvalue
 - Rescale variables?
```

When comparing m3\_random and m4\_random, we can see now is that the incorporation of the session index is not improving the model in terms of the BIC score and is thus, not significant (also has a low t-value < 2).

### Randomized indexes¶

In [103]:

```
m3_random_2 = glmer(num_answers~1+session_comments+(1|author), data = data, family=poisson())
summary(m3_random_2)
```

Out[103]:

```
Generalized linear mixed model fit by maximum likelihood (Laplace
  Approximation) [glmerMod]
 Family: poisson  ( log )
Formula: num_answers ~ 1 + session_comments + (1 | author)
   Data: data

      AIC       BIC    logLik  deviance  df.resid 
1121525.0 1121558.4 -560759.5 1121519.0    515193 

Scaled residuals: 
   Min     1Q Median     3Q    Max 
-8.719 -0.657 -0.598  0.539 42.410 

Random effects:
 Groups Name        Variance Std.Dev.
 author (Intercept) 0.4018   0.6339  
Number of obs: 515196, groups:  author, 288629

Fixed effects:
                  Estimate Std. Error z value Pr(>|z|)    
(Intercept)      -0.730513   0.004365 -167.34   <2e-16 ***
session_comments  0.015978   0.000858   18.62   <2e-16 ***
---
Signif. codes:  0 ‘***’ 0.001 ‘**’ 0.01 ‘*’ 0.05 ‘.’ 0.1 ‘ ’ 1

Correlation of Fixed Effects:
            (Intr)
sssn_cmmnts -0.796
convergence code: 0
Model failed to converge with max|grad| = 0.00474022 (tol = 0.001, component 1)
Model is nearly unidentifiable: very large eigenvalue
 - Rescale variables?
```

In [104]:

```
m4_random_2 = glmer(num_answers~1+session_index_random_2+session_comments+(1|author), data = data, family=poisson())
summary(m4_random_2)
```

Out[104]:

```
Generalized linear mixed model fit by maximum likelihood (Laplace
  Approximation) [glmerMod]
 Family: poisson  ( log )
Formula: num_answers ~ 1 + session_index_random_2 + session_comments +  
    (1 | author)
   Data: data

      AIC       BIC    logLik  deviance  df.resid 
1121526.5 1121571.1 -560759.2 1121518.5    515192 

Scaled residuals: 
   Min     1Q Median     3Q    Max 
-8.724 -0.657 -0.598  0.539 42.410 

Random effects:
 Groups Name        Variance Std.Dev.
 author (Intercept) 0.4018   0.6339  
Number of obs: 515196, groups:  author, 288629

Fixed effects:
                         Estimate Std. Error z value Pr(>|z|)    
(Intercept)            -0.7309949  0.0044251 -165.19   <2e-16 ***
session_index_random_2  0.0009546  0.0014135    0.68    0.499    
session_comments        0.0155018  0.0011117   13.94   <2e-16 ***
---
Signif. codes:  0 ‘***’ 0.001 ‘**’ 0.01 ‘*’ 0.05 ‘.’ 0.1 ‘ ’ 1

Correlation of Fixed Effects:
            (Intr) ss___2
sssn_ndx__2 -0.161       
sssn_cmmnts -0.505 -0.636
convergence code: 0
Model failed to converge with max|grad| = 0.00790221 (tol = 0.001, component 1)
Model is nearly unidentifiable: very large eigenvalue
 - Rescale variables?
```

Again, we see similar behavior, the incorporation of the random session index does not improve the model compared to one that already includes the session length.

Overall, the analyses presented in this notebook have demonstrated that the course of a session has a (negative) effect on the number of answers a post receives.

In [ ]:

```

```
