# Supplementary material for: Evidence of Online Performance Deterioration in User Sessions on Reddit
Source: PLoS One. 2016 Aug 25;11(8):e0161636. doi: 10.1371/journal.pone.0161636 (PMC4999233; doi:10.1371/journal.pone.0161636)
Supplement: S7 Notebook — (HTML) [file pone.0161636.s007.html]

dynamic\_score


In [44]:

```
library(lme4)
options(rgl.useNULL=TRUE)
library(LMERConvenienceFunctions)

library(ggplot2)
options(jupyter.plot_mimetypes = 'image/png')
library(repr)
options(repr.plot.width=6, repr.plot.height=6)

library(car)
library(MASS)

library(vcd)
```

# Score¶

In this notebook, we document our various steps taken to study the effect of the course of a session on the score a comment receives. Note that we only do this on a sample of 1 mio. data points here.

In [45]:

```
data = read.csv("/home/psinger/Reddit-depletion/data/sample.csv", header=TRUE)
```

In [46]:

```
nrow(data)
```

Out[46]:

1000000

## Data inspection¶

We start by getting a better feeling of our data.

We can limit our data to those sessions that have at least two and less than 11 comments.

In [47]:

```
idx = data$session_comments > 1 & data$session_comments < 11
```

In [48]:

```
data = data[idx,]
```

In [49]:

```
nrow(data)
```

Out[49]:

515196

In [50]:

```
hist(data$score)
```

In [51]:

```
hist(data$score[data$score<10&data$score>-10])
```

## Regression model¶

We use mixed-effects models where in the basic form (that we analyze in this notebook), we are interested in studying score ~ 1 + session\_index + session\_comments + (1|author).

### Linear mixed-effects regression¶

We start with a linear mixed-effects model.

In [52]:

```
m_lmer = lmer(score~1+session_index + session_comments+(1|author), data=data, REML=FALSE)
```

In [53]:

```
summary(m_lmer)
```

Out[53]:

```
Linear mixed model fit by maximum likelihood  ['lmerMod']
Formula: score ~ 1 + session_index + session_comments + (1 | author)
   Data: data

     AIC      BIC   logLik deviance df.resid 
 5497695  5497751 -2748843  5497685   515191 

Scaled residuals: 
    Min      1Q  Median      3Q     Max 
-25.258  -0.100  -0.083  -0.053 110.277 

Random effects:
 Groups   Name        Variance Std.Dev.
 author   (Intercept)   18.74   4.329  
 Residual             2504.33  50.043  
Number of obs: 515196, groups:  author, 288629

Fixed effects:
                 Estimate Std. Error t value
(Intercept)       6.22975    0.14724   42.31
session_index    -0.38838    0.05266   -7.38
session_comments  0.14978    0.04063    3.69

Correlation of Fixed Effects:
            (Intr) sssn_n
session_ndx -0.175       
sssn_cmmnts -0.538 -0.652
```

In [54]:

```
mcp.fnc(m_lmer)
```

What we can see in above plot is, that the residuals do not appear to be normally distributed and we can see clear heteroskedasticity. Thus, a linear model does not provide a good fit here.

### Linear mixed-effects regression (log-transform)¶

Next, let us take the log of the response variable and repeat a linear regression. However, we first add a constant to the data in order to make all outcomes positive.

In [55]:

```
min(data$score)
data$score.t = data$score + abs(min(data$score))
```

Out[55]:

-1267

In [56]:

```
m_lmer_log = lmer(log1p(score.t)~1+session_index+session_comments+(1|author), data=data, REML=FALSE)
```

In [57]:

```
summary(m_lmer_log)
```

Out[57]:

```
Linear mixed model fit by maximum likelihood  ['lmerMod']
Formula: log1p(score.t) ~ 1 + session_index + session_comments + (1 |  
    author)
   Data: data

     AIC      BIC   logLik deviance df.resid 
-2256429 -2256373  1128219 -2256439   515191 

Scaled residuals: 
     Min       1Q   Median       3Q      Max 
-261.979   -0.127   -0.105   -0.062   61.584 

Random effects:
 Groups   Name        Variance  Std.Dev.
 author   (Intercept) 1.123e-05 0.003351
 Residual             7.225e-04 0.026879
Number of obs: 515196, groups:  author, 288629

Fixed effects:
                   Estimate Std. Error t value
(Intercept)       7.150e+00  7.948e-05   89954
session_index    -2.361e-04  2.839e-05      -8
session_comments  7.838e-05  2.193e-05       4

Correlation of Fixed Effects:
            (Intr) sssn_n
session_ndx -0.174       
sssn_cmmnts -0.538 -0.652
```

In [58]:

```
mcp.fnc(m_lmer_log)
```

That does not look satisfying at all. We should focus on generalized mixed-effects models to better model the count data.

### Poisson GLMER¶

Let us start with a Poisson GLMER, which is a main choice when working with count data.

In [59]:

```
m_poisson = glmer(score.t~1+session_index+session_comments+(1|author),data=data,family=poisson())
```

```
Warning message:
In checkConv(attr(opt, "derivs"), opt$par, ctrl = control$checkConv, : Model failed to converge with max|grad| = 0.00317215 (tol = 0.001, component 1)Warning message:
In checkConv(attr(opt, "derivs"), opt$par, ctrl = control$checkConv, : Model is nearly unidentifiable: very large eigenvalue
 - Rescale variables?
```

In [60]:

```
summary(m_poisson)
```

Out[60]:

```
Generalized linear mixed model fit by maximum likelihood (Laplace
  Approximation) [glmerMod]
 Family: poisson  ( log )
Formula: score.t ~ 1 + session_index + session_comments + (1 | author)
   Data: data

     AIC      BIC   logLik deviance df.resid 
 5286971  5287016 -2643482  5286963   515192 

Scaled residuals: 
    Min      1Q  Median      3Q     Max 
-31.801  -0.092  -0.067  -0.026  90.279 

Random effects:
 Groups Name        Variance  Std.Dev.
 author (Intercept) 0.0002952 0.01718 
Number of obs: 515196, groups:  author, 288629

Fixed effects:
                   Estimate Std. Error z value Pr(>|z|)    
(Intercept)       7.149e+00  9.572e-05   74692  < 2e-16 ***
session_index    -3.098e-04  3.248e-05     -10  < 2e-16 ***
session_comments  1.054e-04  2.568e-05       4 4.08e-05 ***
---
Signif. codes:  0 ‘***’ 0.001 ‘**’ 0.01 ‘*’ 0.05 ‘.’ 0.1 ‘ ’ 1

Correlation of Fixed Effects:
            (Intr) sssn_n
session_ndx -0.165       
sssn_cmmnts -0.523 -0.637
convergence code: 0
Model failed to converge with max|grad| = 0.00317215 (tol = 0.001, component 1)
Model is nearly unidentifiable: very large eigenvalue
 - Rescale variables?
```

In [61]:

```
plot(fitted(m_poisson),resid(m_poisson))
```

The convergence message shows low magnitude and for such large data this might be a false positive as emphasized in the lme4 docu.

The residual plots for generalized linear models are generally quite difficult to interpret as the residuals do not necessarily need to follow a normal distribution any longer. However, the fitted vs. residual plots shows a typical pattern for Poisson regression with curvilinear traces. What we can do though as a first step here, is to look at overdispersion (see http://glmm.wikidot.com/faq).

In [62]:

```
overdisp_fun <- function(model) {
  ## number of variance parameters in 
  ##   an n-by-n variance-covariance matrix
  vpars <- function(m) {
    nrow(m)*(nrow(m)+1)/2
  }
  model.df <- sum(sapply(VarCorr(model),vpars))+length(fixef(model))
  rdf <- nrow(model.frame(model))-model.df
  rp <- residuals(model,type="pearson")
  Pearson.chisq <- sum(rp^2)
  prat <- Pearson.chisq/rdf
  pval <- pchisq(Pearson.chisq, df=rdf, lower.tail=FALSE)
  c(chisq=Pearson.chisq,ratio=prat,rdf=rdf,p=pval)
}
```

In [63]:

```
overdisp_fun(m_poisson)
```

Out[63]:

chisq
:   502883.576881925

ratio
:   0.97610905620026

rdf
:   515192

p
:   1

It does not seem that we have any problem with overdispersion here.

Let us be sure and check it with the dispersion\_glmer function from the blmeco packages.

In [64]:

```
library("blmeco") 
dispersion_glmer(m_poisson)
```

Out[64]:

1.00454113384361

The value is between 0.75 and 1.4 which gives an indicator that there is no overdispersion problem.

Again, for completeness, we fit a negative binomial regression with quasi likelihood.

In [65]:

```
#just for initialization
init = glm.nb(score.t~1+session_index+session_comments,data=data)
```

In [66]:

```
init$theta
```

Out[66]:

2636.65298197502

In [67]:

```
m_pql_nb = glmmPQL(score.t~1+session_index, random = list(~1 | author), data = data, family = negative.binomial(theta=init$theta))
```

```
iteration 1
```

In [68]:

```
summary(m_pql_nb)
```

Out[68]:

```
Linear mixed-effects model fit by maximum likelihood
 Data: data 
  AIC BIC logLik
   NA  NA     NA

Random effects:
 Formula: ~1 | author
        (Intercept) Residual
StdDev: 0.003408434 1.151876

Variance function:
 Structure: fixed weights
 Formula: ~invwt 
Fixed effects: score.t ~ 1 + session_index 
                  Value    Std.Error     DF  t-value p-value
(Intercept)    7.149542 9.748272e-05 288628 73341.63       0
session_index -0.000206 3.136380e-05 226566    -6.56       0
 Correlation: 
              (Intr)
session_index -0.823

Standardized Within-Group Residuals:
         Min           Q1          Med           Q3          Max 
-25.24565198  -0.09913141  -0.08359083  -0.05338234 110.26378433 

Number of Observations: 515196
Number of Groups: 288629
```

Again, very similar results.

## Final model choice¶

Given our extensive analysis regarding the appropriate model for our data, we can conclude that all have very similar conclusions, namely that the session\_index has a negative effect on score. For the rest of this section, let us focus on Poisson regression that seems to be appropriate for this count data at hand.

## Significance of effects¶

Now that we have decided on a model, we want to extend our inference. First, we want to study the significance of the fixed effects as well as potential additional random effects.

There are several options for determining the significance of a fixed effect on the model. For example, a common approach is to use the ratio of the slope to its standard error and then use a t-test for determining its significance. Alternatively, one can also use F-tests for contrasting simpler to more complex models. Both methods require a specification of the degrees of freedom though which is not trivial in mixed models; methods such as the Satterthwaite approximation or Kenward-Roger approximation can be used for that task.
As stated in Baayen 2008, we can also directly use the t-statistics given to derive statistical significance. Because we have a huge dataset, a t-statistic above 2 can be regarded as significant at the 5\% level in a two-tailed test. You can derive this directly from the results above.

However, here, we follow an approach used for comparing models: the Bayesian Information Criterion (alternatively we could also work with AIC or LRT). Basically, the idea is to specify different (in this case nested) models that successively incorporate additional fixed or random effects and then compare their BIC scores. The lower the BIC, the better a model is (above a small threshold).

The baseline model we start with is:

score = 1 + (1|author)

The reason why we incorporate the random effect for author from the beginning is that this is necessary based on the design of the experiment. Also, as pointed out in http://glmm.wikidot.com/faq, you should not compare the fit of a (g)lmer with one from a (g)lm.

Okay, so let us get the BIC for the baseline model.

In [69]:

```
m1 = glmer(score.t~1+(1|author), data = data, family=poisson())
summary(m1)
```

```
Warning message:
In checkConv(attr(opt, "derivs"), opt$par, ctrl = control$checkConv, : Model failed to converge with max|grad| = 0.00309017 (tol = 0.001, component 1)Warning message:
In checkConv(attr(opt, "derivs"), opt$par, ctrl = control$checkConv, : Model is nearly unidentifiable: very large eigenvalue
 - Rescale variables?
```

Out[69]:

```
Generalized linear mixed model fit by maximum likelihood (Laplace
  Approximation) [glmerMod]
 Family: poisson  ( log )
Formula: score.t ~ 1 + (1 | author)
   Data: data

     AIC      BIC   logLik deviance df.resid 
 5287064  5287087 -2643530  5287060   515194 

Scaled residuals: 
    Min      1Q  Median      3Q     Max 
-31.791  -0.093  -0.071  -0.029  90.268 

Random effects:
 Groups Name        Variance  Std.Dev.
 author (Intercept) 0.0002953 0.01718 
Number of obs: 515196, groups:  author, 288629

Fixed effects:
             Estimate Std. Error z value Pr(>|z|)    
(Intercept) 7.149e+00  5.325e-05  134260   <2e-16 ***
---
Signif. codes:  0 ‘***’ 0.001 ‘**’ 0.01 ‘*’ 0.05 ‘.’ 0.1 ‘ ’ 1
convergence code: 0
Model failed to converge with max|grad| = 0.00309017 (tol = 0.001, component 1)
Model is nearly unidentifiable: very large eigenvalue
 - Rescale variables?
```

Next, let us incorporate the index and session length individually.

In [70]:

```
m2 = glmer(score.t~1+session_index+(1|author), data = data, family=poisson())
summary(m2)
```

```
Warning message:
In checkConv(attr(opt, "derivs"), opt$par, ctrl = control$checkConv, : Model failed to converge with max|grad| = 0.00225972 (tol = 0.001, component 1)Warning message:
In checkConv(attr(opt, "derivs"), opt$par, ctrl = control$checkConv, : Model is nearly unidentifiable: very large eigenvalue
 - Rescale variables?
```

Out[70]:

```
Generalized linear mixed model fit by maximum likelihood (Laplace
  Approximation) [glmerMod]
 Family: poisson  ( log )
Formula: score.t ~ 1 + session_index + (1 | author)
   Data: data

     AIC      BIC   logLik deviance df.resid 
 5286986  5287019 -2643490  5286980   515193 

Scaled residuals: 
    Min      1Q  Median      3Q     Max 
-31.797  -0.096  -0.067  -0.026  90.267 

Random effects:
 Groups Name        Variance  Std.Dev.
 author (Intercept) 0.0002953 0.01718 
Number of obs: 515196, groups:  author, 288629

Fixed effects:
                Estimate Std. Error z value Pr(>|z|)    
(Intercept)    7.149e+00  8.159e-05   87621   <2e-16 ***
session_index -2.249e-04  2.505e-05      -9   <2e-16 ***
---
Signif. codes:  0 ‘***’ 0.001 ‘**’ 0.01 ‘*’ 0.05 ‘.’ 0.1 ‘ ’ 1

Correlation of Fixed Effects:
            (Intr)
session_ndx -0.758
convergence code: 0
Model failed to converge with max|grad| = 0.00225972 (tol = 0.001, component 1)
Model is nearly unidentifiable: very large eigenvalue
 - Rescale variables?
```

In [71]:

```
m3 = glmer(score.t~1+session_comments+(1|author), data = data, family=poisson())
summary(m3)
```

```
Warning message:
In checkConv(attr(opt, "derivs"), opt$par, ctrl = control$checkConv, : Model failed to converge with max|grad| = 0.00397523 (tol = 0.001, component 1)Warning message:
In checkConv(attr(opt, "derivs"), opt$par, ctrl = control$checkConv, : Model is nearly unidentifiable: very large eigenvalue
 - Rescale variables?
```

Out[71]:

```
Generalized linear mixed model fit by maximum likelihood (Laplace
  Approximation) [glmerMod]
 Family: poisson  ( log )
Formula: score.t ~ 1 + session_comments + (1 | author)
   Data: data

     AIC      BIC   logLik deviance df.resid 
 5287060  5287093 -2643527  5287054   515193 

Scaled residuals: 
    Min      1Q  Median      3Q     Max 
-31.790  -0.094  -0.069  -0.029  90.263 

Random effects:
 Groups Name        Variance  Std.Dev.
 author (Intercept) 0.0002953 0.01718 
Number of obs: 515196, groups:  author, 288629

Fixed effects:
                   Estimate Std. Error z value Pr(>|z|)    
(Intercept)       7.149e+00  9.440e-05   75731   <2e-16 ***
session_comments -5.053e-05  1.980e-05      -3   0.0107 *  
---
Signif. codes:  0 ‘***’ 0.001 ‘**’ 0.01 ‘*’ 0.05 ‘.’ 0.1 ‘ ’ 1

Correlation of Fixed Effects:
            (Intr)
sssn_cmmnts -0.826
convergence code: 0
Model failed to converge with max|grad| = 0.00397523 (tol = 0.001, component 1)
Model is nearly unidentifiable: very large eigenvalue
 - Rescale variables?
```

Based on the lower BIC we can see that the incorporation of the session\_index improves our model, thus, we make inference on it (also the t-value indicates high significance). The session\_comments alone are not significant; let's check incorporating both.

In [72]:

```
m4 = glmer(score.t~1+session_index+session_comments+(1|author), data = data, family=poisson())
summary(m4)
```

```
Warning message:
In checkConv(attr(opt, "derivs"), opt$par, ctrl = control$checkConv, : Model failed to converge with max|grad| = 0.00317215 (tol = 0.001, component 1)Warning message:
In checkConv(attr(opt, "derivs"), opt$par, ctrl = control$checkConv, : Model is nearly unidentifiable: very large eigenvalue
 - Rescale variables?
```

Out[72]:

```
Generalized linear mixed model fit by maximum likelihood (Laplace
  Approximation) [glmerMod]
 Family: poisson  ( log )
Formula: score.t ~ 1 + session_index + session_comments + (1 | author)
   Data: data

     AIC      BIC   logLik deviance df.resid 
 5286971  5287016 -2643482  5286963   515192 

Scaled residuals: 
    Min      1Q  Median      3Q     Max 
-31.801  -0.092  -0.067  -0.026  90.279 

Random effects:
 Groups Name        Variance  Std.Dev.
 author (Intercept) 0.0002952 0.01718 
Number of obs: 515196, groups:  author, 288629

Fixed effects:
                   Estimate Std. Error z value Pr(>|z|)    
(Intercept)       7.149e+00  9.572e-05   74692  < 2e-16 ***
session_index    -3.098e-04  3.248e-05     -10  < 2e-16 ***
session_comments  1.054e-04  2.568e-05       4 4.08e-05 ***
---
Signif. codes:  0 ‘***’ 0.001 ‘**’ 0.01 ‘*’ 0.05 ‘.’ 0.1 ‘ ’ 1

Correlation of Fixed Effects:
            (Intr) sssn_n
session_ndx -0.165       
sssn_cmmnts -0.523 -0.637
convergence code: 0
Model failed to converge with max|grad| = 0.00317215 (tol = 0.001, component 1)
Model is nearly unidentifiable: very large eigenvalue
 - Rescale variables?
```

As expected based on our initial empirical analysis in the paper, both the session index and session length are important effects to incorporate. The significance for session\_index is less pronounced though as for the other experiments; however, for the complete data reported in the paper, strong significiance is given.

We stop at this point as our scope of interest is covered. However, one can arbitrarily extend the existing model.
For example, one might also argue that it is necessary to incorporate a random effect for the intercept based on different subreddits.

In [73]:

```
m5 = glmer(score.t~1+session_index+session_comments+(1|author)+(1|subreddit), data = data, family=poisson())
summary(m5)
```

```
Warning message:
In checkConv(attr(opt, "derivs"), opt$par, ctrl = control$checkConv, : Model failed to converge with max|grad| = 0.0564161 (tol = 0.001, component 1)Warning message:
In checkConv(attr(opt, "derivs"), opt$par, ctrl = control$checkConv, : Model is nearly unidentifiable: very large eigenvalue
 - Rescale variables?
```

Out[73]:

```
Generalized linear mixed model fit by maximum likelihood (Laplace
  Approximation) [glmerMod]
 Family: poisson  ( log )
Formula: score.t ~ 1 + session_index + session_comments + (1 | author) +  
    (1 | subreddit)
   Data: data

     AIC      BIC   logLik deviance df.resid 
 5284397  5284452 -2642193  5284387   515191 

Scaled residuals: 
    Min      1Q  Median      3Q     Max 
-31.806  -0.102  -0.046  -0.002  90.232 

Random effects:
 Groups    Name        Variance  Std.Dev.
 author    (Intercept) 2.898e-04 0.017022
 subreddit (Intercept) 2.640e-06 0.001625
Number of obs: 515196, groups:  author, 288629; subreddit, 12041

Fixed effects:
                   Estimate Std. Error z value Pr(>|z|)    
(Intercept)       7.148e+00  1.088e-04   65684   <2e-16 ***
session_index    -3.228e-04  3.246e-05     -10   <2e-16 ***
session_comments  7.380e-05  2.571e-05       3   0.0041 ** 
---
Signif. codes:  0 ‘***’ 0.001 ‘**’ 0.01 ‘*’ 0.05 ‘.’ 0.1 ‘ ’ 1

Correlation of Fixed Effects:
            (Intr) sssn_n
session_ndx -0.144       
sssn_cmmnts -0.446 -0.635
convergence code: 0
Model failed to converge with max|grad| = 0.0564161 (tol = 0.001, component 1)
Model is nearly unidentifiable: very large eigenvalue
 - Rescale variables?
```

Again, the BIC shows an improvement in the model. Yet, the inference on the fixed effects does not change. For complexity reasons, we neglect the random effect for the subreddit for the complete data.

### Collinearity¶

We also want to check for multicollinearity in our model; some explanations and code for checking that in lme4 is provided in https://hlplab.wordpress.com/2011/02/24/diagnosing-collinearity-in-lme4/.

In [74]:

```
source("../mer-utils.R")
kappa.mer(m4)
max(vif.mer(m4))
```

Out[74]:

4.48954996169921

Out[74]:

1.68117610385677

Both the condition number and VIF show no reasons to be concerned about collinearity in this model.

### Length as random effect¶

We have decided to incorporate the variation between different session lengths as a controlling covariate, however we might also incorporate it as a controlling random effect.

In [75]:

```
m4_control = glmer(score.t~1+session_index+(1|session_comments)+(1|author), data = data, family=poisson())
summary(m4_control)
```

```
Warning message:
In checkConv(attr(opt, "derivs"), opt$par, ctrl = control$checkConv, : Model failed to converge with max|grad| = 16.194 (tol = 0.001, component 1)Warning message:
In checkConv(attr(opt, "derivs"), opt$par, ctrl = control$checkConv, : Model is nearly unidentifiable: very large eigenvalue
 - Rescale variables?
```

Out[75]:

```
Generalized linear mixed model fit by maximum likelihood (Laplace
  Approximation) [glmerMod]
 Family: poisson  ( log )
Formula: score.t ~ 1 + session_index + (1 | session_comments) + (1 | author)
   Data: data

     AIC      BIC   logLik deviance df.resid 
 5287002  5287047 -2643497  5286994   515192 

Scaled residuals: 
    Min      1Q  Median      3Q     Max 
-31.763  -0.095  -0.068  -0.025  89.944 

Random effects:
 Groups           Name        Variance  Std.Dev.
 author           (Intercept) 3.003e-04 0.017330
 session_comments (Intercept) 1.211e-07 0.000348
Number of obs: 515196, groups:  author, 288629; session_comments, 9

Fixed effects:
                Estimate Std. Error z value Pr(>|z|)    
(Intercept)    7.150e+00  1.888e-04   37863   <2e-16 ***
session_index -3.723e-04  3.447e-05     -11   <2e-16 ***
---
Signif. codes:  0 ‘***’ 0.001 ‘**’ 0.01 ‘*’ 0.05 ‘.’ 0.1 ‘ ’ 1

Correlation of Fixed Effects:
            (Intr)
session_ndx -0.690
convergence code: 0
Model failed to converge with max|grad| = 16.194 (tol = 0.001, component 1)
Model is nearly unidentifiable: very large eigenvalue
 - Rescale variables?
```

The results are similar.

## Convergence problems¶

Convergence warnings (which we partly also see for experiments on the complete data) are most likely false positives and do not effect the results. The gold standard approach to investigate this is to utilize different optimizers and compare the coefficients. If they are not similar, our inference is stable which is the case here. The code is based on Ben Bolkers code presented online: http://stackoverflow.com/questions/21344555/convergence-error-for-development-version-of-lme4

In [85]:

```
g0.bobyqa <- glmer(score.t~1+session_index+session_comments+(1|author),
      family = poisson, data = data,
                   control=glmerControl(optimizer="bobyqa"))
g0.NM <- update(g0.bobyqa,control=glmerControl(optimizer="Nelder_Mead"))
library(optimx)
g0.nlminb <- update(g0.bobyqa,control=glmerControl(optimizer="optimx",
                              optCtrl=list(method="nlminb")))
g0.LBFGSB <- update(g0.bobyqa,control=glmerControl(optimizer="optimx",
                              optCtrl=list(method="L-BFGS-B")))

library(nloptr)
## from https://github.com/lme4/lme4/issues/98:
defaultControl <- list(algorithm="NLOPT_LN_BOBYQA",xtol_rel=1e-6,maxeval=1e5)
nloptwrap2 <- function(fn,par,lower,upper,control=list(),...) {
    for (n in names(defaultControl)) 
      if (is.null(control[[n]])) control[[n]] <- defaultControl[[n]]
    res <- nloptr(x0=par,eval_f=fn,lb=lower,ub=upper,opts=control,...)
    with(res,list(par=solution,
                  fval=objective,
                  feval=iterations,
                  conv=if (status>0) 0 else status,
                  message=message))
}
g0.bobyqa2 <- update(g0.bobyqa,control=glmerControl(optimizer=nloptwrap2))
g0.NM2 <- update(g0.bobyqa,control=glmerControl(optimizer=nloptwrap2,
                           optCtrl=list(algorithm="NLOPT_LN_NELDERMEAD")))
```

```
Warning message:
In checkConv(attr(opt, "derivs"), opt$par, ctrl = control$checkConv, : Model failed to converge with max|grad| = 0.0525527 (tol = 0.001, component 1)Warning message:
In checkConv(attr(opt, "derivs"), opt$par, ctrl = control$checkConv, : Model is nearly unidentifiable: very large eigenvalue
 - Rescale variables?Warning message:
In checkConv(attr(opt, "derivs"), opt$par, ctrl = control$checkConv, : Model failed to converge with max|grad| = 0.00803934 (tol = 0.001, component 1)Warning message:
In checkConv(attr(opt, "derivs"), opt$par, ctrl = control$checkConv, : Model is nearly unidentifiable: very large eigenvalue
 - Rescale variables?Warning message:
In optimx.check(par, optcfg$ufn, optcfg$ugr, optcfg$uhess, lower, : Parameters or bounds appear to have different scalings.
  This can cause poor performance in optimization. 
  It is important for derivative free methods like BOBYQA, UOBYQA, NEWUOA.Warning message:
In optwrap(optimizer, devfun, start, rho$lower, control = control, : convergence code 1 from optimxWarning message:
In checkConv(attr(opt, "derivs"), opt$par, ctrl = control$checkConv, : Model failed to converge with max|grad| = 1.00577 (tol = 0.001, component 1)Warning message:
In checkConv(attr(opt, "derivs"), opt$par, ctrl = control$checkConv, : Model is nearly unidentifiable: very large eigenvalue
 - Rescale variables?Warning message:
In optimx.check(par, optcfg$ufn, optcfg$ugr, optcfg$uhess, lower, : Parameters or bounds appear to have different scalings.
  This can cause poor performance in optimization. 
  It is important for derivative free methods like BOBYQA, UOBYQA, NEWUOA.Warning message:
In checkConv(attr(opt, "derivs"), opt$par, ctrl = control$checkConv, : Model failed to converge with max|grad| = 0.540356 (tol = 0.001, component 1)Warning message:
In checkConv(attr(opt, "derivs"), opt$par, ctrl = control$checkConv, : Model is nearly unidentifiable: very large eigenvalue
 - Rescale variables?Warning message:
In checkConv(attr(opt, "derivs"), opt$par, ctrl = control$checkConv, : Model failed to converge with max|grad| = 2.25709 (tol = 0.001, component 1)Warning message:
In checkConv(attr(opt, "derivs"), opt$par, ctrl = control$checkConv, : Model is nearly unidentifiable: very large eigenvalue
 - Rescale variables?Warning message:
In checkConv(attr(opt, "derivs"), opt$par, ctrl = control$checkConv, : Model failed to converge with max|grad| = 0.00446455 (tol = 0.001, component 1)Warning message:
In checkConv(attr(opt, "derivs"), opt$par, ctrl = control$checkConv, : Model is nearly unidentifiable: very large eigenvalue
 - Rescale variables?
```

In [86]:

```
getpar <- function(x) c(getME(x,c("theta")),fixef(x))
modList <- list(bobyqa=g0.bobyqa,NM=g0.NM,nlminb=g0.nlminb,
                bobyqa2=g0.bobyqa2,NM2=g0.NM2,LBFGSB=g0.LBFGSB)
ctab <- sapply(modList,getpar)
library(reshape2)
mtab <- melt(ctab)
library(ggplot2)
theme_set(theme_bw())
ggplot(mtab,aes(x=Var2,y=value,colour=Var2))+
    geom_point()+facet_wrap(~Var1,scale="free")
```

## Randomized baseline data¶

In order to finally confirm our intuition and inference on the negative effect of the session index on the number of responses, we also study our randomized baseline data. We basically have two different randomized datasets: (i) new sessions based on randomized time differences and (ii) randomized positions inside existing sessions.

In [78]:

```
idx = data$session_comments_random > 1
data_random = data[idx,]
```

In [79]:

```
m1_random = glmer(score.t~1+(1|author), data = data_random, family=poisson())
summary(m1_random)
```

```
Warning message:
In checkConv(attr(opt, "derivs"), opt$par, ctrl = control$checkConv, : Model failed to converge with max|grad| = 0.0017097 (tol = 0.001, component 1)Warning message:
In checkConv(attr(opt, "derivs"), opt$par, ctrl = control$checkConv, : Model is nearly unidentifiable: very large eigenvalue
 - Rescale variables?
```

Out[79]:

```
Generalized linear mixed model fit by maximum likelihood (Laplace
  Approximation) [glmerMod]
 Family: poisson  ( log )
Formula: score.t ~ 1 + (1 | author)
   Data: data_random

     AIC      BIC   logLik deviance df.resid 
 3993292  3993314 -1996644  3993288   388336 

Scaled residuals: 
    Min      1Q  Median      3Q     Max 
-26.714  -0.091  -0.069  -0.027  88.683 

Random effects:
 Groups Name        Variance  Std.Dev.
 author (Intercept) 0.0003202 0.0179  
Number of obs: 388338, groups:  author, 216454

Fixed effects:
             Estimate Std. Error z value Pr(>|z|)    
(Intercept) 7.149e+00  6.248e-05  114424   <2e-16 ***
---
Signif. codes:  0 ‘***’ 0.001 ‘**’ 0.01 ‘*’ 0.05 ‘.’ 0.1 ‘ ’ 1
convergence code: 0
Model failed to converge with max|grad| = 0.0017097 (tol = 0.001, component 1)
Model is nearly unidentifiable: very large eigenvalue
 - Rescale variables?
```

In [80]:

```
m2_random = glmer(score.t~1+session_index_random+(1|author), data = data_random, family=poisson())
summary(m2_random)
```

```
Warning message:
In checkConv(attr(opt, "derivs"), opt$par, ctrl = control$checkConv, : Model failed to converge with max|grad| = 0.0055438 (tol = 0.001, component 1)Warning message:
In checkConv(attr(opt, "derivs"), opt$par, ctrl = control$checkConv, : Model is nearly unidentifiable: very large eigenvalue
 - Rescale variables?
```

Out[80]:

```
Generalized linear mixed model fit by maximum likelihood (Laplace
  Approximation) [glmerMod]
 Family: poisson  ( log )
Formula: score.t ~ 1 + session_index_random + (1 | author)
   Data: data_random

     AIC      BIC   logLik deviance df.resid 
 3993293  3993326 -1996644  3993287   388335 

Scaled residuals: 
    Min      1Q  Median      3Q     Max 
-26.712  -0.092  -0.069  -0.028  88.683 

Random effects:
 Groups Name        Variance  Std.Dev.
 author (Intercept) 0.0003202 0.0179  
Number of obs: 388338, groups:  author, 216454

Fixed effects:
                       Estimate Std. Error z value Pr(>|z|)    
(Intercept)           7.149e+00  7.553e-05   94645   <2e-16 ***
session_index_random -1.678e-05  1.471e-05      -1    0.254    
---
Signif. codes:  0 ‘***’ 0.001 ‘**’ 0.01 ‘*’ 0.05 ‘.’ 0.1 ‘ ’ 1

Correlation of Fixed Effects:
            (Intr)
sssn_ndx_rn -0.562
convergence code: 0
Model failed to converge with max|grad| = 0.0055438 (tol = 0.001, component 1)
Model is nearly unidentifiable: very large eigenvalue
 - Rescale variables?
```

In [81]:

```
m3_random = glmer(score.t~1+session_comments_random+(1|author), data = data_random, family=poisson())
summary(m3_random)
```

```
Warning message:
In checkConv(attr(opt, "derivs"), opt$par, ctrl = control$checkConv, : Model failed to converge with max|grad| = 0.0351677 (tol = 0.001, component 1)Warning message:
In checkConv(attr(opt, "derivs"), opt$par, ctrl = control$checkConv, : Model is nearly unidentifiable: very large eigenvalue
 - Rescale variables?
```

Out[81]:

```
Generalized linear mixed model fit by maximum likelihood (Laplace
  Approximation) [glmerMod]
 Family: poisson  ( log )
Formula: score.t ~ 1 + session_comments_random + (1 | author)
   Data: data_random

     AIC      BIC   logLik deviance df.resid 
 3993294  3993327 -1996644  3993288   388335 

Scaled residuals: 
    Min      1Q  Median      3Q     Max 
-26.715  -0.091  -0.069  -0.028  88.684 

Random effects:
 Groups Name        Variance  Std.Dev.
 author (Intercept) 0.0003202 0.0179  
Number of obs: 388338, groups:  author, 216454

Fixed effects:
                         Estimate Std. Error z value Pr(>|z|)    
(Intercept)             7.149e+00  7.792e-05   91743   <2e-16 ***
session_comments_random 3.044e-06  9.735e-06       0    0.755    
---
Signif. codes:  0 ‘***’ 0.001 ‘**’ 0.01 ‘*’ 0.05 ‘.’ 0.1 ‘ ’ 1

Correlation of Fixed Effects:
            (Intr)
sssn_cmmnt_ -0.598
convergence code: 0
Model failed to converge with max|grad| = 0.0351677 (tol = 0.001, component 1)
Model is nearly unidentifiable: very large eigenvalue
 - Rescale variables?
```

In [82]:

```
m4_random = glmer(score.t~1+session_index_random+session_comments_random+(1|author), data = data_random, family=poisson())
summary(m4_random)
```

```
Warning message:
In checkConv(attr(opt, "derivs"), opt$par, ctrl = control$checkConv, : Model failed to converge with max|grad| = 0.0216727 (tol = 0.001, component 1)Warning message:
In checkConv(attr(opt, "derivs"), opt$par, ctrl = control$checkConv, : Model is nearly unidentifiable: very large eigenvalue
 - Rescale variables?
```

Out[82]:

```
Generalized linear mixed model fit by maximum likelihood (Laplace
  Approximation) [glmerMod]
 Family: poisson  ( log )
Formula: score.t ~ 1 + session_index_random + session_comments_random +  
    (1 | author)
   Data: data_random

     AIC      BIC   logLik deviance df.resid 
 3993292  3993336 -1996642  3993284   388334 

Scaled residuals: 
    Min      1Q  Median      3Q     Max 
-26.711  -0.091  -0.069  -0.028  88.685 

Random effects:
 Groups Name        Variance  Std.Dev.
 author (Intercept) 0.0003202 0.0179  
Number of obs: 388338, groups:  author, 216454

Fixed effects:
                          Estimate Std. Error z value Pr(>|z|)    
(Intercept)              7.149e+00  7.888e-05   90633   <2e-16 ***
session_index_random    -4.349e-05  2.162e-05      -2   0.0442 *  
session_comments_random  2.414e-05  1.431e-05       2   0.0916 .  
---
Signif. codes:  0 ‘***’ 0.001 ‘**’ 0.01 ‘*’ 0.05 ‘.’ 0.1 ‘ ’ 1

Correlation of Fixed Effects:
            (Intr) sssn_n_
sssn_ndx_rn -0.155        
sssn_cmmnt_ -0.288 -0.733 
convergence code: 0
Model failed to converge with max|grad| = 0.0216727 (tol = 0.001, component 1)
Model is nearly unidentifiable: very large eigenvalue
 - Rescale variables?
```

When comparing m3\_random and m4\_random, we can see now is that the incorporation of the session index is not improving the model in terms of the BIC score and is thus, not significant (also has a low t-value < 2).

The reason why the session length itself is still significant can be explained by our very conservative randomization method that just randomizes the time differences between comments before sessionizing and thus, still keeps many sessions intact.

### Randomized indexes¶

In [83]:

```
m3_random_2 = glmer(score.t~1+session_comments+(1|author), data = data, family=poisson())
summary(m3_random_2)
```

```
Warning message:
In checkConv(attr(opt, "derivs"), opt$par, ctrl = control$checkConv, : Model failed to converge with max|grad| = 0.00397523 (tol = 0.001, component 1)Warning message:
In checkConv(attr(opt, "derivs"), opt$par, ctrl = control$checkConv, : Model is nearly unidentifiable: very large eigenvalue
 - Rescale variables?
```

Out[83]:

```
Generalized linear mixed model fit by maximum likelihood (Laplace
  Approximation) [glmerMod]
 Family: poisson  ( log )
Formula: score.t ~ 1 + session_comments + (1 | author)
   Data: data

     AIC      BIC   logLik deviance df.resid 
 5287060  5287093 -2643527  5287054   515193 

Scaled residuals: 
    Min      1Q  Median      3Q     Max 
-31.790  -0.094  -0.069  -0.029  90.263 

Random effects:
 Groups Name        Variance  Std.Dev.
 author (Intercept) 0.0002953 0.01718 
Number of obs: 515196, groups:  author, 288629

Fixed effects:
                   Estimate Std. Error z value Pr(>|z|)    
(Intercept)       7.149e+00  9.440e-05   75731   <2e-16 ***
session_comments -5.053e-05  1.980e-05      -3   0.0107 *  
---
Signif. codes:  0 ‘***’ 0.001 ‘**’ 0.01 ‘*’ 0.05 ‘.’ 0.1 ‘ ’ 1

Correlation of Fixed Effects:
            (Intr)
sssn_cmmnts -0.826
convergence code: 0
Model failed to converge with max|grad| = 0.00397523 (tol = 0.001, component 1)
Model is nearly unidentifiable: very large eigenvalue
 - Rescale variables?
```

In [84]:

```
m4_random_2 = glmer(score.t~1+session_index_random_2+session_comments+(1|author), data = data, family=poisson())
summary(m4_random_2)
```

```
Warning message:
In checkConv(attr(opt, "derivs"), opt$par, ctrl = control$checkConv, : Model failed to converge with max|grad| = 0.00529796 (tol = 0.001, component 1)Warning message:
In checkConv(attr(opt, "derivs"), opt$par, ctrl = control$checkConv, : Model is nearly unidentifiable: very large eigenvalue
 - Rescale variables?
```

Out[84]:

```
Generalized linear mixed model fit by maximum likelihood (Laplace
  Approximation) [glmerMod]
 Family: poisson  ( log )
Formula: score.t ~ 1 + session_index_random_2 + session_comments + (1 |  
    author)
   Data: data

     AIC      BIC   logLik deviance df.resid 
 5287061  5287106 -2643527  5287053   515192 

Scaled residuals: 
    Min      1Q  Median      3Q     Max 
-31.791  -0.094  -0.069  -0.029  90.263 

Random effects:
 Groups Name        Variance  Std.Dev.
 author (Intercept) 0.0002953 0.01718 
Number of obs: 515196, groups:  author, 288629

Fixed effects:
                         Estimate Std. Error z value Pr(>|z|)    
(Intercept)             7.149e+00  9.581e-05   74614   <2e-16 ***
session_index_random_2  2.269e-05  3.252e-05       1   0.4854    
session_comments       -6.185e-05  2.560e-05      -2   0.0157 *  
---
Signif. codes:  0 ‘***’ 0.001 ‘**’ 0.01 ‘*’ 0.05 ‘.’ 0.1 ‘ ’ 1

Correlation of Fixed Effects:
            (Intr) ss___2
sssn_ndx__2 -0.171       
sssn_cmmnts -0.521 -0.634
convergence code: 0
Model failed to converge with max|grad| = 0.00529796 (tol = 0.001, component 1)
Model is nearly unidentifiable: very large eigenvalue
 - Rescale variables?
```

Again, we see similar behavior, the incorporation of the random session index does not improve the model compared to one that already includes the session length.

Overall, the analyses presented in this notebook have demonstrated that the course of a session has a (negative) effect on the score a post receives.

In [ ]:

```

```
