# Supplementary material for: Evidence of Online Performance Deterioration in User Sessions on Reddit
Source: PLoS One. 2016 Aug 25;11(8):e0161636. doi: 10.1371/journal.pone.0161636 (PMC4999233; doi:10.1371/journal.pone.0161636)
Supplement: S8 Notebook — (HTML) [file pone.0161636.s008.html]

dynamic\_readability


In [34]:

```
library(lme4)
options(rgl.useNULL=TRUE)
library(LMERConvenienceFunctions)

library(ggplot2)
options(jupyter.plot_mimetypes = 'image/png')
library(repr)
options(repr.plot.width=6, repr.plot.height=6)

library(car)
library(MASS)

library(vcd)
```

# Number of answers¶

In this notebook, we document our various steps taken to study the effect of the course of a session on the readability of a comment. Note that we only do this on a sample of 1 mio. data points here.

In [35]:

```
data = read.csv("/home/psinger/Reddit-depletion/data/sample.csv", header=TRUE)
```

In [36]:

```
nrow(data)
```

Out[36]:

1000000

## Data inspection¶

We start by getting a better feeling of our data.

We can limit our data to those sessions that have at least two and less than 11 comments.

In [75]:

```
idx = data$session_comments > 1 & data$session_comments < 11
```

In [76]:

```
data = data[idx,]
```

In [77]:

```
nrow(data)
```

Out[77]:

515196

In [40]:

```
hist(data$flesch_kincaid_grade)
```

## Regression model¶

We use mixed-effects models where in the basic form (that we analyze in this notebook), we are interested in studying flesch\_kincaid\_grade ~ 1 + session\_index + session\_comments + (1|author).

### Linear mixed-effects regression¶

We start with a linear mixed-effects model.

In [41]:

```
m_lmer = lmer(flesch_kincaid_grade~1+session_index + session_comments+(1|author), data=data, REML=FALSE)
```

In [42]:

```
summary(m_lmer)
```

Out[42]:

```
Linear mixed model fit by maximum likelihood  ['lmerMod']
Formula: flesch_kincaid_grade ~ 1 + session_index + session_comments +  
    (1 | author)
   Data: data

     AIC      BIC   logLik deviance df.resid 
 3022180  3022235 -1511085  3022170   515191 

Scaled residuals: 
    Min      1Q  Median      3Q     Max 
-2.7563 -0.5824 -0.0425  0.5172  4.4641 

Random effects:
 Groups   Name        Variance Std.Dev.
 author   (Intercept)  2.398   1.548   
 Residual             18.483   4.299   
Number of obs: 515196, groups:  author, 288629

Fixed effects:
                  Estimate Std. Error t value
(Intercept)       5.166885   0.013473   383.5
session_index    -0.049985   0.004727   -10.6
session_comments  0.029821   0.003690     8.1

Correlation of Fixed Effects:
            (Intr) sssn_n
session_ndx -0.171       
sssn_cmmnts -0.533 -0.645
```

In [43]:

```
mcp.fnc(m_lmer)
```

While not perfect, the residuals do not look to bad for large data here. The deviations from normality might be explained by the peaks in data at both ends of the distribution (large and low readability). Let us quickly try two transformations on the outcome (sqrt and log) for which we first set the outcome positive by adding a constant.

In [44]:

```
min(data$flesch_kincaid_grade)
data$flesch_kincaid_grade.t = data$flesch_kincaid_grade + 4.4
```

Out[44]:

-3.4

### Linear mixed-effects regression (transform)¶

In [45]:

```
m_lmer_log = lmer(log(flesch_kincaid_grade.t)~1+session_index+session_comments+(1|author), data=data, REML=FALSE)
```

In [46]:

```
summary(m_lmer_log)
```

Out[46]:

```
Linear mixed model fit by maximum likelihood  ['lmerMod']
Formula: log(flesch_kincaid_grade.t) ~ 1 + session_index + session_comments +  
    (1 | author)
   Data: data

      AIC       BIC    logLik  deviance  df.resid 
 979480.2  979535.9 -489735.1  979470.2    515191 

Scaled residuals: 
    Min      1Q  Median      3Q     Max 
-3.7893 -0.2984  0.1913  0.5767  3.0365 

Random effects:
 Groups   Name        Variance Std.Dev.
 author   (Intercept) 0.04399  0.2097  
 Residual             0.35187  0.5932  
Number of obs: 515196, groups:  author, 288629

Fixed effects:
                   Estimate Std. Error t value
(Intercept)       2.1057910  0.0018552  1135.1
session_index    -0.0074840  0.0006514   -11.5
session_comments  0.0046150  0.0005083     9.1

Correlation of Fixed Effects:
            (Intr) sssn_n
session_ndx -0.171       
sssn_cmmnts -0.533 -0.645
```

In [47]:

```
mcp.fnc(m_lmer_log)
```

In [48]:

```
m_lmer_sqrt = lmer(sqrt(flesch_kincaid_grade.t)~1+session_index+session_comments+(1|author), data=data, REML=FALSE)
summary(m_lmer_sqrt)
```

Out[48]:

```
Linear mixed model fit by maximum likelihood  ['lmerMod']
Formula: sqrt(flesch_kincaid_grade.t) ~ 1 + session_index + session_comments +  
    (1 | author)
   Data: data

      AIC       BIC    logLik  deviance  df.resid 
1203487.2 1203543.0 -601738.6 1203477.2    515191 

Scaled residuals: 
    Min      1Q  Median      3Q     Max 
-3.1318 -0.4763  0.0796  0.5807  3.7529 

Random effects:
 Groups   Name        Variance Std.Dev.
 author   (Intercept) 0.07206  0.2684  
 Residual             0.54010  0.7349  
Number of obs: 515196, groups:  author, 288629

Fixed effects:
                   Estimate Std. Error t value
(Intercept)       2.9918465  0.0023068  1297.0
session_index    -0.0092526  0.0008089   -11.4
session_comments  0.0056309  0.0006316     8.9

Correlation of Fixed Effects:
            (Intr) sssn_n
session_ndx -0.171       
sssn_cmmnts -0.532 -0.645
```

In [49]:

```
mcp.fnc(m_lmer_sqrt)
```

Looks quite similar to the non-transformed.

Let us focus on a potentially more appropriate Gamma generalized linear mixed-effects model next though.

### Gamma GLMER¶

Let us model the data with a Gamma GLMER using a log link.

In [50]:

```
m_gamma = glmer(flesch_kincaid_grade.t~1+session_index+session_comments+(1|author),data=data,family=Gamma(link=log))
summary(m_gamma)
```

```
Warning message:
In checkConv(attr(opt, "derivs"), opt$par, ctrl = control$checkConv, : Model failed to converge with max|grad| = 1.23812 (tol = 0.001, component 1)Warning message:
In checkConv(attr(opt, "derivs"), opt$par, ctrl = control$checkConv, : Model is nearly unidentifiable: very large eigenvalue
 - Rescale variables?
```

Out[50]:

```
Generalized linear mixed model fit by maximum likelihood (Laplace
  Approximation) [glmerMod]
 Family: Gamma  ( log )
Formula: flesch_kincaid_grade.t ~ 1 + session_index + session_comments +  
    (1 | author)
   Data: data

     AIC      BIC   logLik deviance df.resid 
 3016252  3016308 -1508121  3016242   515191 

Scaled residuals: 
    Min      1Q  Median      3Q     Max 
-2.1083 -0.4755  0.0732  0.5848  8.0399 

Random effects:
 Groups   Name        Variance Std.Dev.
 author   (Intercept) 0.0669   0.2586  
 Residual             0.1926   0.4388  
Number of obs: 515196, groups:  author, 288629

Fixed effects:
                   Estimate Std. Error t value Pr(>|z|)    
(Intercept)       2.176e+00  5.648e-05   38524   <2e-16 ***
session_index    -5.926e-03  5.568e-05    -106   <2e-16 ***
session_comments  4.575e-03  5.476e-05      84   <2e-16 ***
---
Signif. codes:  0 ‘***’ 0.001 ‘**’ 0.01 ‘*’ 0.05 ‘.’ 0.1 ‘ ’ 1

Correlation of Fixed Effects:
            (Intr) sssn_n
session_ndx -0.007       
sssn_cmmnts -0.011 -0.038
convergence code: 0
Model failed to converge with max|grad| = 1.23812 (tol = 0.001, component 1)
Model is nearly unidentifiable: very large eigenvalue
 - Rescale variables?
```

In [51]:

```
plot(fitted(m_gamma),resid(m_gamma))
```

The convergence message shows low magnitude and for such large data this might be a false positive as emphasized in the lme4 docu.

The coefficients are quite similar to our previous analyses.

## Final model choice¶

Given our extensive analysis regarding the appropriate model for our data, we can conclude that all have very similar conclusions, namely that the session\_index has a negative effect on the Flesch-Kincaid grade meaning that readability complexity decreases. Nonetheless, we will proceed with a linear model on the flesch\_kincaid\_grade due to the reasonable diagnostic plots and the simplicity of the model. We thus take slight deviations from normality of the residuals into account, the residuals do not indicate too much heteroskedasticity.

## Significance of effects¶

Now that we have decided on a model, we want to extend our inference. First, we want to study the significance of the fixed effects as well as potential additional random effects.

There are several options for determining the significance of a fixed effect on the model. For example, a common approach is to use the ratio of the slope to its standard error and then use a t-test for determining its significance. Alternatively, one can also use F-tests for contrasting simpler to more complex models. Both methods require a specification of the degrees of freedom though which is not trivial in mixed models; methods such as the Satterthwaite approximation or Kenward-Roger approximation can be used for that task.
As stated in Baayen 2008, we can also directly use the t-statistics given to derive statistical significance. Because we have a huge dataset, a t-statistic above 2 can be regarded as significant at the 5\% level in a two-tailed test. You can derive this directly from the results above.

However, here, we follow an approach used for comparing models: the Bayesian Information Criterion (alternatively we could also work with AIC or LRT). Basically, the idea is to specify different (in this case nested) models that successively incorporate additional fixed or random effects and then compare their BIC scores. The lower the BIC, the better a model is (above a small threshold).

The baseline model we start with is:

flesch\_kincaid\_grade = 1 + (1|author)

The reason why we incorporate the random effect for author from the beginning is that this is necessary based on the design of the experiment. Also, as pointed out in http://glmm.wikidot.com/faq, you should not compare the fit of a (g)lmer with one from a (g)lm.

Okay, so let us get the BIC for the baseline model.

In [52]:

```
m1 = lmer(flesch_kincaid_grade~1+(1|author), data = data, REML=FALSE)
summary(m1)
```

Out[52]:

```
Linear mixed model fit by maximum likelihood  ['lmerMod']
Formula: flesch_kincaid_grade ~ 1 + (1 | author)
   Data: data

     AIC      BIC   logLik deviance df.resid 
 3022290  3022324 -1511142  3022284   515193 

Scaled residuals: 
    Min      1Q  Median      3Q     Max 
-2.7719 -0.5856 -0.0407  0.5173  4.4908 

Random effects:
 Groups   Name        Variance Std.Dev.
 author   (Intercept)  2.398   1.549   
 Residual             18.487   4.300   
Number of obs: 515196, groups:  author, 288629

Fixed effects:
            Estimate Std. Error t value
(Intercept) 5.161243   0.006916   746.3
```

Next, let us incorporate the index and session length individually.

In [53]:

```
m2 = lmer(flesch_kincaid_grade~1+session_index+(1|author), data = data, REML=FALSE)
summary(m2)
```

Out[53]:

```
Linear mixed model fit by maximum likelihood  ['lmerMod']
Formula: flesch_kincaid_grade ~ 1 + session_index + (1 | author)
   Data: data

     AIC      BIC   logLik deviance df.resid 
 3022243  3022288 -1511117  3022235   515192 

Scaled residuals: 
    Min      1Q  Median      3Q     Max 
-2.7674 -0.5841 -0.0427  0.5172  4.4896 

Random effects:
 Groups   Name        Variance Std.Dev.
 author   (Intercept)  2.398   1.549   
 Residual             18.485   4.299   
Number of obs: 515196, groups:  author, 288629

Fixed effects:
               Estimate Std. Error t value
(Intercept)    5.224865   0.011405   458.1
session_index -0.025352   0.003614    -7.0

Correlation of Fixed Effects:
            (Intr)
session_ndx -0.795
```

In [54]:

```
m3 = lmer(flesch_kincaid_grade~1+session_comments+(1|author), data = data, REML=FALSE)
summary(m3)
```

Out[54]:

```
Linear mixed model fit by maximum likelihood  ['lmerMod']
Formula: flesch_kincaid_grade ~ 1 + session_comments + (1 | author)
   Data: data

     AIC      BIC   logLik deviance df.resid 
 3022289  3022334 -1511141  3022281   515192 

Scaled residuals: 
    Min      1Q  Median      3Q     Max 
-2.7708 -0.5848 -0.0416  0.5172  4.4870 

Random effects:
 Groups   Name        Variance Std.Dev.
 author   (Intercept)  2.398   1.549   
 Residual             18.487   4.300   
Number of obs: 515196, groups:  author, 288629

Fixed effects:
                 Estimate Std. Error t value
(Intercept)      5.142509   0.013276   387.4
session_comments 0.004663   0.002821     1.7

Correlation of Fixed Effects:
            (Intr)
sssn_cmmnts -0.854
```

Based on the lower BIC we can see that the incorporation of the session\_index improves our model, thus, we make inference on it (also the t-value indicates high significance). The session\_comments alone are not significant; let's check incorporating both.

In [55]:

```
m4 = lmer(flesch_kincaid_grade~1+session_index+session_comments+(1|author), data = data, REML=FALSE)
summary(m4)
```

Out[55]:

```
Linear mixed model fit by maximum likelihood  ['lmerMod']
Formula: flesch_kincaid_grade ~ 1 + session_index + session_comments +  
    (1 | author)
   Data: data

     AIC      BIC   logLik deviance df.resid 
 3022180  3022235 -1511085  3022170   515191 

Scaled residuals: 
    Min      1Q  Median      3Q     Max 
-2.7563 -0.5824 -0.0425  0.5172  4.4641 

Random effects:
 Groups   Name        Variance Std.Dev.
 author   (Intercept)  2.398   1.548   
 Residual             18.483   4.299   
Number of obs: 515196, groups:  author, 288629

Fixed effects:
                  Estimate Std. Error t value
(Intercept)       5.166885   0.013473   383.5
session_index    -0.049985   0.004727   -10.6
session_comments  0.029821   0.003690     8.1

Correlation of Fixed Effects:
            (Intr) sssn_n
session_ndx -0.171       
sssn_cmmnts -0.533 -0.645
```

As expected based on our initial empirical analysis in the paper, both the session index and session length are important effects to incorporate.

We stop at this point as our scope of interest is covered. However, one can arbitrarily extend the existing model.
For example, one might also argue that it is necessary to incorporate a random effect for the intercept based on different subreddits.

In [56]:

```
m5 = lmer(flesch_kincaid_grade~1+session_index+session_comments+(1|author)+(1|subreddit), data = data, REML=FALSE)
summary(m5)
```

Out[56]:

```
Linear mixed model fit by maximum likelihood  ['lmerMod']
Formula: flesch_kincaid_grade ~ 1 + session_index + session_comments +  
    (1 | author) + (1 | subreddit)
   Data: data

     AIC      BIC   logLik deviance df.resid 
 3004837  3004904 -1502413  3004825   515190 

Scaled residuals: 
    Min      1Q  Median      3Q     Max 
-4.1007 -0.5896 -0.0455  0.5138  4.7623 

Random effects:
 Groups    Name        Variance Std.Dev.
 author    (Intercept)  1.644   1.282   
 subreddit (Intercept)  1.617   1.272   
 Residual              18.181   4.264   
Number of obs: 515196, groups:  author, 288629; subreddit, 12041

Fixed effects:
                  Estimate Std. Error t value
(Intercept)       5.098165   0.023442  217.48
session_index    -0.049415   0.004647  -10.63
session_comments  0.035441   0.003636    9.75

Correlation of Fixed Effects:
            (Intr) sssn_n
session_ndx -0.097       
sssn_cmmnts -0.278 -0.643
```

Again, the BIC shows an improvement in the model. Yet, the inference on the fixed effects does not change. For complexity reasons, we neglect the random effect for the subreddit for the complete data.

### Collinearity¶

We also want to check for multicollinearity in our model; some explanations and code for checking that in lme4 is provided in https://hlplab.wordpress.com/2011/02/24/diagnosing-collinearity-in-lme4/.

In [57]:

```
source("../mer-utils.R")
kappa.mer(m4)
max(vif.mer(m4))
```

Out[57]:

4.48954996169921

Out[57]:

1.711595643544

Both the condition number and VIF show no reasons to be concerned about collinearity in this model.

### Length as random effect¶

We have decided to incorporate the variation between different session lengths as a controlling covariate, however we might also incorporate it as a controlling random effect.

In [58]:

```
m4_control = lmer(flesch_kincaid_grade~1+session_index+(1|session_comments)+(1|author), data = data, REML=FALSE)
summary(m4_control)
```

Out[58]:

```
Linear mixed model fit by maximum likelihood  ['lmerMod']
Formula: flesch_kincaid_grade ~ 1 + session_index + (1 | session_comments) +  
    (1 | author)
   Data: data

     AIC      BIC   logLik deviance df.resid 
 3022188  3022244 -1511089  3022178   515191 

Scaled residuals: 
    Min      1Q  Median      3Q     Max 
-2.7577 -0.5825 -0.0417  0.5170  4.4812 

Random effects:
 Groups           Name        Variance  Std.Dev.
 author           (Intercept)  2.396680 1.54812 
 session_comments (Intercept)  0.003924 0.06264 
 Residual                     18.483594 4.29926 
Number of obs: 515196, groups:  author, 288629; session_comments, 9

Fixed effects:
              Estimate Std. Error t value
(Intercept)    5.32617    0.02741  194.28
session_index -0.04655    0.00456  -10.21

Correlation of Fixed Effects:
            (Intr)
session_ndx -0.565
```

The results are similar.

## Convergence problems¶

Convergence warnings (which we partly also see for experiments on the complete data) are most likely false positives and do not effect the results. The gold standard approach to investigate this is to utilize different optimizers and compare the coefficients. If they are not similar, our inference is stable which is the case here. The code is based on Ben Bolkers code presented online: http://stackoverflow.com/questions/21344555/convergence-error-for-development-version-of-lme4

In [70]:

```
g0.bobyqa <- lmer(flesch_kincaid_grade~1+session_index+session_comments+(1|author),
      data = data,
                   control=lmerControl(optimizer="bobyqa"))
g0.NM <- update(g0.bobyqa,control=lmerControl(optimizer="Nelder_Mead"))
library(optimx)
g0.nlminb <- update(g0.bobyqa,control=lmerControl(optimizer="optimx",
                              optCtrl=list(method="nlminb")))
g0.LBFGSB <- update(g0.bobyqa,control=lmerControl(optimizer="optimx",
                              optCtrl=list(method="L-BFGS-B")))
```

```
Error in control$optimizer == "none": comparison (1) is possible only for atomic and list types
```

```
Error in control$optimizer == "none": comparison (1) is possible only for atomic and list types
```

In [72]:

```
getpar <- function(x) c(getME(x,c("theta")),fixef(x))
modList <- list(bobyqa=g0.bobyqa,NM=g0.NM,nlminb=g0.nlminb,
                LBFGSB=g0.LBFGSB)
ctab <- sapply(modList,getpar)
library(reshape2)
mtab <- melt(ctab)
library(ggplot2)
theme_set(theme_bw())
ggplot(mtab,aes(x=Var2,y=value,colour=Var2))+
    geom_point()+facet_wrap(~Var1,scale="free")
```

## Randomized baseline data¶

In order to finally confirm our intuition and inference on the negative effect of the session index on the number of responses, we also study our randomized baseline data. We basically have two different randomized datasets: (i) new sessions based on randomized time differences and (ii) randomized positions inside existing sessions.

In [61]:

```
idx = data$session_comments_random > 1
data_random = data[idx,]
```

In [62]:

```
m1_random = lmer(flesch_kincaid_grade~1+(1|author), data = data_random, REML=FALSE)
summary(m1_random)
```

Out[62]:

```
Linear mixed model fit by maximum likelihood  ['lmerMod']
Formula: flesch_kincaid_grade ~ 1 + (1 | author)
   Data: data_random

     AIC      BIC   logLik deviance df.resid 
 2277452  2277484 -1138723  2277446   388335 

Scaled residuals: 
    Min      1Q  Median      3Q     Max 
-2.7391 -0.5864 -0.0407  0.5182  4.4867 

Random effects:
 Groups   Name        Variance Std.Dev.
 author   (Intercept)  2.361   1.536   
 Residual             18.486   4.300   
Number of obs: 388338, groups:  author, 216454

Fixed effects:
            Estimate Std. Error t value
(Intercept) 5.161875   0.007959   648.6
```

In [63]:

```
m2_random = lmer(flesch_kincaid_grade~1+session_index_random+(1|author), data = data_random, REML=FALSE)
summary(m2_random)
```

Out[63]:

```
Linear mixed model fit by maximum likelihood  ['lmerMod']
Formula: flesch_kincaid_grade ~ 1 + session_index_random + (1 | author)
   Data: data_random

     AIC      BIC   logLik deviance df.resid 
 2277453  2277497 -1138723  2277445   388334 

Scaled residuals: 
    Min      1Q  Median      3Q     Max 
-2.7396 -0.5862 -0.0412  0.5182  4.4862 

Random effects:
 Groups   Name        Variance Std.Dev.
 author   (Intercept)  2.36    1.536   
 Residual             18.49    4.300   
Number of obs: 388338, groups:  author, 216454

Fixed effects:
                      Estimate Std. Error t value
(Intercept)           5.165116   0.010098   511.5
session_index_random -0.001077   0.002067    -0.5

Correlation of Fixed Effects:
            (Intr)
sssn_ndx_rn -0.615
```

In [64]:

```
m3_random = lmer(flesch_kincaid_grade~1+session_comments_random+(1|author), data = data_random, REML=FALSE)
summary(m3_random)
```

Out[64]:

```
Linear mixed model fit by maximum likelihood  ['lmerMod']
Formula: flesch_kincaid_grade ~ 1 + session_comments_random + (1 | author)
   Data: data_random

     AIC      BIC   logLik deviance df.resid 
 2277445  2277488 -1138718  2277437   388334 

Scaled residuals: 
    Min      1Q  Median      3Q     Max 
-2.7372 -0.5850 -0.0421  0.5186  4.5289 

Random effects:
 Groups   Name        Variance Std.Dev.
 author   (Intercept)  2.358   1.536   
 Residual             18.488   4.300   
Number of obs: 388338, groups:  author, 216454

Fixed effects:
                         Estimate Std. Error t value
(Intercept)              5.181603   0.010388   498.8
session_comments_random -0.003921   0.001328    -3.0

Correlation of Fixed Effects:
            (Intr)
sssn_cmmnt_ -0.643
```

In [65]:

```
m4_random = lmer(flesch_kincaid_grade~1+session_index_random+session_comments_random+(1|author), data = data_random, REML=FALSE)
summary(m4_random)
```

Out[65]:

```
Linear mixed model fit by maximum likelihood  ['lmerMod']
Formula: 
flesch_kincaid_grade ~ 1 + session_index_random + session_comments_random +  
    (1 | author)
   Data: data_random

     AIC      BIC   logLik deviance df.resid 
 2277440  2277494 -1138715  2277430   388333 

Scaled residuals: 
    Min      1Q  Median      3Q     Max 
-2.7316 -0.5849 -0.0423  0.5186  4.5749 

Random effects:
 Groups   Name        Variance Std.Dev.
 author   (Intercept)  2.358   1.535   
 Residual             18.488   4.300   
Number of obs: 388338, groups:  author, 216454

Fixed effects:
                         Estimate Std. Error t value
(Intercept)              5.176937   0.010542   491.1
session_index_random     0.008179   0.003148     2.6
session_comments_random -0.007885   0.002022    -3.9

Correlation of Fixed Effects:
            (Intr) sssn_n_
sssn_ndx_rn -0.170        
sssn_cmmnt_ -0.287 -0.754
```

When comparing m3\_random and m4\_random, we can see now is that the incorporation of the session index is not improving the model in terms of the BIC score and is thus, not significant (also has a low t-value < 2).

### Randomized indexes¶

In [66]:

```
m3_random_2 = lmer(flesch_kincaid_grade~1+session_comments+(1|author), data = data, REML=FALSE)
summary(m3_random_2)
```

Out[66]:

```
Linear mixed model fit by maximum likelihood  ['lmerMod']
Formula: flesch_kincaid_grade ~ 1 + session_comments + (1 | author)
   Data: data

     AIC      BIC   logLik deviance df.resid 
 3022289  3022334 -1511141  3022281   515192 

Scaled residuals: 
    Min      1Q  Median      3Q     Max 
-2.7708 -0.5848 -0.0416  0.5172  4.4870 

Random effects:
 Groups   Name        Variance Std.Dev.
 author   (Intercept)  2.398   1.549   
 Residual             18.487   4.300   
Number of obs: 515196, groups:  author, 288629

Fixed effects:
                 Estimate Std. Error t value
(Intercept)      5.142509   0.013276   387.4
session_comments 0.004663   0.002821     1.7

Correlation of Fixed Effects:
            (Intr)
sssn_cmmnts -0.854
```

In [67]:

```
m4_random_2 = lmer(flesch_kincaid_grade~1+session_index_random_2+session_comments+(1|author), data = data, REML=FALSE)
summary(m4_random_2)
```

Out[67]:

```
Linear mixed model fit by maximum likelihood  ['lmerMod']
Formula: 
flesch_kincaid_grade ~ 1 + session_index_random_2 + session_comments +  
    (1 | author)
   Data: data

     AIC      BIC   logLik deviance df.resid 
 3022291  3022347 -1511141  3022281   515191 

Scaled residuals: 
    Min      1Q  Median      3Q     Max 
-2.7708 -0.5847 -0.0416  0.5172  4.4867 

Random effects:
 Groups   Name        Variance Std.Dev.
 author   (Intercept)  2.398   1.549   
 Residual             18.487   4.300   
Number of obs: 515196, groups:  author, 288629

Fixed effects:
                         Estimate Std. Error t value
(Intercept)             5.1428726  0.0134881   381.3
session_index_random_2 -0.0007214  0.0047330    -0.2
session_comments        0.0050235  0.0036793     1.4

Correlation of Fixed Effects:
            (Intr) ss___2
sssn_ndx__2 -0.177       
sssn_cmmnts -0.531 -0.642
```

Again, we see similar behavior, the incorporation of the random session index does not improve the model compared to one that already includes the session length.

Overall, the analyses presented in this notebook have demonstrated that the course of a session has a (negative) effect on the Flesch-Kincaid grade a comment received.
