# Supplementary material for: Evidence of Online Performance Deterioration in User Sessions on Reddit
Source: PLoS One. 2016 Aug 25;11(8):e0161636. doi: 10.1371/journal.pone.0161636 (PMC4999233; doi:10.1371/journal.pone.0161636)
Supplement: S1 Table — This table presents the detailed mixed-effects model results for studying the effect of session length on the text length of the first comment C1 in a session; i.e., data only contains the first session comments. The models at hand are linear mixed-effects models (lmer) where the outcome (text length) has been log-transformed. The baseline model excludes the fixed effect at interest for judging the significance of the effect; comparing the BIC of both models reveals a clear significance. This is confirmed by the AIC as well as the classic t-test on the coefficient. (PDF) [file pone.0161636.s009.pdf]

	Baseline Model	Effect Model
(Intercept)	4.34009***	4.29109***
	(0.00054)	(0.00059)
session_comments		0.03417***
		(0.00017)
AIC	73115854.00475	73076845.25224
BIC	73115898.90597	73076905.12054
Log Likelihood	-36557924.00237	-36538418.62612
Num. obs.	23372562	23372562
Num. groups: author	2581810	2581810
Var: author (Intercept)	0.40135	0.39952
Var: Residual	1.19954	1.19776

$^{***}p < 0.001$, $^{**}p < 0.01$, $^{*}p < 0.05$
