# Supplementary material for: Evidence of Online Performance Deterioration in User Sessions on Reddit
Source: PLoS One. 2016 Aug 25;11(8):e0161636. doi: 10.1371/journal.pone.0161636 (PMC4999233; doi:10.1371/journal.pone.0161636)
Supplement: S3 Table — This table presents the detailed mixed-effects model results for studying the effect of session length on the score of the first comment C1 in a session; i.e., data only contains the first session comments. The models at hand are generalized linear Poisson mixed-effects models (glmer) with a log link—additionally we have added a constant for making the score always positive. The baseline model excludes the fixed effect at interest for judging the significance of the effect; comparing the BIC of both models reveals a clear significance. This is confirmed by the AIC as well as the classic t-test on the coefficient. (PDF) [file pone.0161636.s011.pdf]

	Baseline Model	Effect Model
(Intercept)	7.22615***	7.22592***
	(0.00001)	(0.00001)
session_comments		0.00015***
		(0.00000)
AIC	245396210.87785	245394889.32770
BIC	245396240.81200	245394934.22892
Log Likelihood	-122698103.43893	-122697441.66385
Num. obs.	23372562	23372562
Num. groups: author	2581810	2581810
Var: author (Intercept)	0.00011	0.00011

$^{***}p < 0.001$, $^{**}p < 0.01$, $^{*}p < 0.05$
