# Supplementary material for: Evidence of Online Performance Deterioration in User Sessions on Reddit
Source: PLoS One. 2016 Aug 25;11(8):e0161636. doi: 10.1371/journal.pone.0161636 (PMC4999233; doi:10.1371/journal.pone.0161636)
Supplement: S4 Table — This table presents the detailed mixed-effects model results for studying the effect of session length on the readability of the first comment C1 in a session; i.e., data only contains the first session comments. The models at hand are linear mixed-effects models (lmer). The baseline model excludes the fixed effect at interest for judging the significance of the effect; comparing the BIC of both models reveals a clear significance. This is confirmed by the AIC as well as the classic t-test on the coefficient. (PDF) [file pone.0161636.s012.pdf]

	Baseline Model	Effect Model
(Intercept)	4.99683***	4.92595***
	(0.00165)	(0.00194)
session_comments		0.04784***
		(0.00068)
AIC	136761897.03228	136756989.22804
BIC	136761941.93350	136757049.09634
Log Likelihood	-68380945.51614	-68378490.61402
Num. obs.	23372562	23372562
Num. groups: author	2581810	2581810
Var: author (Intercept)	2.56832	2.56645
Var: Residual	19.10481	19.10121

$^{***}p < 0.001$, $^{**}p < 0.01$, $^{*}p < 0.05$
