# Supplementary material for: Evidence of Online Performance Deterioration in User Sessions on Reddit
Source: PLoS One. 2016 Aug 25;11(8):e0161636. doi: 10.1371/journal.pone.0161636 (PMC4999233; doi:10.1371/journal.pone.0161636)
Supplement: S5 Table — This table presents the detailed mixed-effects model results for studying the effect of the session index i on the text length of respective comment Ci; i.e., data includes all session comments. The models at hand are linear mixed-effects models (lmer) where the outcome (text length) has been log-transformed. The baseline model excludes the fixed effect at interest for judging the significance of the effect; comparing the BIC of both models reveals a clear significance. This is confirmed by the AIC as well as the classic t-test on the coefficient. (PDF) [file pone.0161636.s013.pdf]

	Baseline Model	Effect Model
(Intercept)	4.40404***	4.41430***
	(0.00078)	(0.00079)
session_comments	0.01033***	0.02059***
	(0.00011)	(0.00014)
session_index		-0.02051^{***}
		(0.00016)
AIC	74219375.25934	74203512.54638
BIC	74219435.29778	74203587.59443
Log Likelihood	-37109683.62967	-37101751.27319
Num. obs.	24388192	24388192
Num. groups: author	1255811	1255811
Var: author (Intercept)	0.41502	0.41515
Var: Residual	1.13942	1.13864

$^{***}p < 0.001$, $^{**}p < 0.01$, $^{*}p < 0.05$
