# Supplementary material for: Evidence of Online Performance Deterioration in User Sessions on Reddit
Source: PLoS One. 2016 Aug 25;11(8):e0161636. doi: 10.1371/journal.pone.0161636 (PMC4999233; doi:10.1371/journal.pone.0161636)
Supplement: S6 Table — This table presents the detailed mixed-effects model results for studying the effect of the session index i on the number of responses of respective comment Ci; i.e., data includes all session comments. The models at hand are generalized linear Poisson mixed-effects models (glmer) with a log link. The baseline model excludes the fixed effect at interest for judging the significance of the effect; comparing the BIC of both models reveals a clear significance. This is confirmed by the AIC as well as the classic t-test on the coefficient. (PDF) [file pone.0161636.s014.pdf]

	Baseline Model	Effect Model
(Intercept)	-0.63543^{***}	-0.59971^{***}
	(0.00074)	(0.00074)
session_comments	0.01833^{***}	0.04850^{***}
	(0.00012)	(0.00015)
session_index		-0.06396^{***}
		(0.00019)
AIC	54524738.33390	54408346.95081
BIC	54524783.36273	54408406.98924
Log Likelihood	-27262366.16695	-27204169.47540
Num. obs.	24388192	24388192
Num. groups: author	1255811	1255811
Var: author (Intercept)	0.19672	0.19670

$^{***}p < 0.001$, $^{**}p < 0.01$, $^{*}p < 0.05$
