# Supplementary material for: Evidence of Online Performance Deterioration in User Sessions on Reddit
Source: PLoS One. 2016 Aug 25;11(8):e0161636. doi: 10.1371/journal.pone.0161636 (PMC4999233; doi:10.1371/journal.pone.0161636)
Supplement: S7 Table — This table presents the detailed mixed-effects model results for studying the effect of the session index i on the score of respective comment Ci; i.e., data includes all session comments. The models at hand are generalized linear Poisson mixed-effects models (glmer) with a log link—additionally we have added a constant for making the score always positive. The baseline model excludes the fixed effect at interest for judging the significance of the effect; comparing the BIC of both models reveals a clear significance. This is confirmed by the AIC as well as the classic t-test on the coefficient. (PDF) [file pone.0161636.s015.pdf]

	Baseline Model	Effect Model
(Intercept)	7.22587^{***}	7.22601^{***}
	(0.00001)	(0.00001)
session_comments	-0.00002^{***}	0.00012^{***}
	(0.00000)	(0.00000)
session_index		-0.00028^{***}
		(0.00000)
AIC	253256197.83849	253251558.63815
BIC	253256242.86732	253251618.67659
Log Likelihood	-126628095.91924	-126625775.31908
Num. obs.	24388192	24388192
Num. groups: author	1255811	1255811
Var: author (Intercept)	0.00007	0.00007

$^{***}p < 0.001$, $^{**}p < 0.01$, $^{*}p < 0.05$
