# Supplementary material for: Evidence of Online Performance Deterioration in User Sessions on Reddit
Source: PLoS One. 2016 Aug 25;11(8):e0161636. doi: 10.1371/journal.pone.0161636 (PMC4999233; doi:10.1371/journal.pone.0161636)
Supplement: S8 Table — This table presents the detailed mixed-effects model results for studying the effect of the session index i on the readability of respective comment Ci; i.e., data includes all session comments. The models at hand are linear mixed-effects models (lmer). The baseline model excludes the fixed effect at interest for judging the significance of the effect; comparing the BIC of both models reveals a clear significance. This is confirmed by the AIC as well as the classic t-test on the coefficient. (PDF) [file pone.0161636.s016.pdf]

	Baseline Model	Effect Model
(Intercept)	5.02969***	5.05023***
	(0.00258)	(0.00260)
session_comments	0.01129***	0.03183***
	(0.00043)	(0.00054)
session_index		-0.04109***
		(0.00065)
AIC	141225001.69449	141221024.62794
BIC	141225061.73293	141221099.67598
Log Likelihood	-70612496.84724	-70610507.31397
Num. obs.	24388192	24388192
Num. groups: author	1255811	1255811
Var: author (Intercept)	2.99832	2.99867
Var: Residual	18.22184	18.21872

$^{***}p < 0.001$, $^{**}p < 0.01$, $^{*}p < 0.05$
